# Organizational culture, social capital, and emergency capacity in primary healthcare institutions: A cross-sectional structural equation modeling study comparing ordinary and older communities

**DOI:** 10.1371/journal.pone.0351875

**Published:** 2026-06-30

**Authors:** Xianhong Huang, Jiamin Tang, Jie Jia, Ling Zou, Kaidi Sun, Zhengnan Meng, Xiaoting Zhang

**Affiliations:** 1 School of Public Administration, Hangzhou Normal University, Hangzhou, China; 2 University Teaching Archives Management, Archives, Hangzhou Normal University, Hangzhou, China; Guangxi Normal University, CHINA

## Abstract

The emergency capacity of primary healthcare institutions is critical to the effectiveness of grassroots emergency management. This study examines the relationships among organizational culture, social capital, and the emergency capacity of primary healthcare institutions using a structural equation modeling approach. A questionnaire survey was conducted among healthcare professionals, yielding 983 valid responses for analysis. The results indicate that organizational culture, as well as structural, relational, and cognitive dimensions of social capital, are significantly associated with the emergency capacity of primary healthcare institutions within the model. In addition, social capital demonstrates mediating roles in the relationship between organizational culture and emergency capacity. Multi-group structural equation modeling further reveals variations across community types: relational social capital shows stronger associations with emergency capacity in ordinary communities, whereas structural social capital is more prominent in older communities. These findings provide empirical evidence on how organizational culture and social capital are linked to emergency capacity in primary healthcare settings, highlighting the importance of both internal cultural development and external social resources in different phases of emergency management.

## Introduction

Primary healthcare institutions form the cornerstone of China’s healthcare system and serve as both a “health safety net” for public health and “whistleblowers” for epidemics [1]. They are the frontline defense in mitigating health risks during crises. The health emergency capacity of primary healthcare institutions is crucial to minimize risk of losses, accelerate post-disaster recovery, and maximize the protection of people’s lives and property [2]. Effective emergency management relies on the health emergency capabilities of these institutions [3]. The health emergency capacity of primary healthcare institutions refers to their comprehensive ability to mobilize resources and implement effective responses related to emergency preparedness, monitoring, and early warning, emergency response, and post-event recovery to prevent and address sudden public health incidents. This includes capacities for emergency prevention, emergency preparedness, emergency response, and post-disaster recovery [4]. However, in recent years, the decentralization of emergency management in China has slowed, rendering the disaster prevention, mitigation, and relief efforts of primary healthcare institutions relatively passive [5]. The extensive damage caused by large-scale disasters and public health emergencies highlights numerous deficiencies in the emergency health response capacity of primary healthcare institutions [6]. These deficiencies include limited risk-assessment capabilities, inadequate emergency supplies and supply chain management, and insufficient internal and external organizational coordination. Weak health emergency capacity in primary healthcare institutions can lead to interruptions in medical services, ineffective epidemic control, and a decline in the quality of medical services, thereby exacerbating social burden and triggering instability [7]. Consequently, there is an urgent need to investigate the factors influencing the health emergency capacity of primary healthcare institutions and explore strategies for their enhancement.

Numerous scholars have extensively explored the factors influencing health emergency capacity, typically following a “problem-cause-strategy” framework. These studies often focus on organizational management [8], emergency resource allocation [9], personnel capabilities [10], infrastructure [11], and social forces [12]. However, existing research largely remains at the “management level,” emphasizing resource allocation and professional training for healthcare professionals while paying relatively little attention to “awareness level” factors, particularly cultural influences. Studies of largescale disasters and public health emergencies have identified organizational culture as a critical strategic element for enhancing health emergency capacity [13]. “Organizational culture” refers to the collective consciousness, shared values, ethical principles, and normative expectations that organizational members develop and uphold through organizational practices under specific socio-economic conditions [14,15]. A constructive organizational culture can subtly motivate members to engage proactively in emergency-related tasks and can strengthen organizational cohesion and response capability. Building on this understanding, organizational culture theory emphasizes that cultural attributes within organizations shape members’ behaviors and decision-making processes [16]. Although organizational culture is an intangible and enduring attribute of organizations, it is often examined as an isolated factor, with limited empirical research situating it within broader relational frameworks. Furthermore, studies show that elements of social networks such as participation, interaction, and trust are closely related to the improvement of primary healthcare emergency capacity [17]. Therefore, it is essential to consider the dynamic interrelations and synergistic effects of these factors. In this context, social capital provides a more comprehensive research perspective. According to social capital theory, social capital comprises resources embedded in social networks, which enhance social efficiency and generate added value by facilitating coordination and action among participants [18]. Social networks, participation, trust, reciprocity, and norms are widely recognized as key elements of social capital. High levels of social capital can promote the flow of information, resource sharing, and collaborative cooperation within and between primary healthcare institutions, thereby enhancing the efficiency and rapid response of emergency rescue actions and improving overall emergency capacity [19]. However, existing research has generally treated organizational culture and social capital as separate explanatory variables, paying limited attention to their underlying interrelationship and the pathways through which they operate within a unified analytical framework.

Notably, the health emergency capacity of primary healthcare institutions may be influenced by community conditions. Significant differences exist between ordinary and older communities in terms of economic and cultural environments, stock of social capital, and availability of emergency facilities [20,21]. Compared to primary healthcare institutions in ordinary communities, those in older communities face more constraints in performing health emergency work. In China, older communities primarily consist of residential areas built before 2000, funded by the government, or organizations with aging infrastructure and surrounding environments that lack long-term management mechanisms [22]. Ordinary communities are relatively new, consisting of residential areas built after 2000 with better infrastructure and property management, including affordable housing and commercial residential areas [23]. Compared to ordinary communities, older communities face multiple structural challenges, such as high population density, outdated facilities, and a disproportionately aging population. These structural disparities hinder the ability of primary healthcare institutions to effectively integrate resources, transmit information, and respond to public health emergencies. However, research on how differences in community types affect the emergency response capabilities of primary healthcare institutions is limited. Current studies on the health emergency capacity of primary healthcare institutions are qualitative, and few examine the mechanisms of how organizational culture and social capital affect this capacity in relation to differences between community types. Additionally, these studies lack corresponding multi-group analyses. Multi-group analysis allows specification of structural equation models (SEMs). Unlike traditional methods, such as factor and path analyses, multi-group analysis not only elucidates the complex network of relationships among a set of variables but also tests the differences in the measurement model across different groups.

Therefore, focusing on primary healthcare institutions, this study draws on organizational culture theory and social capital theory to examine: (1) assess the impact of organizational culture and social capital on the health emergency capacity of primary healthcare institutions; (2) examine the mediating role of social capital in the relationship between organizational culture and health emergency capacity; and (3) investigate whether the influence of organizational culture and social capital on health emergency capacity varies across different types of communities where these primary healthcare institutions are located. By addressing these research objectives, the study sought to offer innovative insights and strategic recommendations for the development and enhancement of emergency response capabilities in primary healthcare institutions.

## Literature review and hypotheses

### The impact of organizational culture on health emergency capacity

The functions of organizational culture include fostering internal integration and coordination by sustaining organizational stability and cultivating a sense of belonging and altruism, all of which guide and shape employees’ behaviors. Theory of organizational culture further emphasizes that shared beliefs and cultural values are embedded in strategic goals and organizational ideology, becoming internalized in members’ cognitions and thereby influencing their attitudes, motivation, and collaborative behaviors [24]. Previous research has demonstrated that positive organizational climates and shared value orientations are linked to more coherent collective responses in crisis situations [25,26]. An analysis of tertiary hospital organizational culture by Reem et al. revealed a positive correlation between cultural norms, organizational goals, and emergency response capacity [27]. For example, a study conducted in China revealed that a culture emphasizing moral compliance encouraged rule-following behavior and stabilized communities, thereby facilitating the rapid mobilization and effective implementation of grassroots pandemic control efforts, ultimately enhancing emergency capacity [28]. Further empirical evidence supports the notion that the level of cultural competence within healthcare institutions is significantly and positively correlated with emergency preparedness and response [29]. Primary healthcare institutions with strong organizational culture are thus better positioned to influence coordinated responses to emergencies. Accordingly, we propose the following hypothesis:

H1: Organizational culture positively affects the health emergency capacity of primary healthcare institutions.

### The impact of social capital on health emergency capacity

Nahapiet and Ghoshal [30] developed a framework for social capital dimensions, describing it across three key dimensions: structural, relational, and cognitive. Structural social capital refers to social interactions such as social relationships, networks, and participation in social activities [31]. Relational social capital pertains to reciprocity and support, particularly perceptions and assessments of trust [32]. Cognitive social capital reflects social norms and institutional practices [31,32]. These three dimensions collectively serve as vital protective resources for organizations or communities when facing external threats, enhancing their adaptability and collaborative capabilities.

Previous research suggest that social capital is associated with the performance of primary healthcare institutions in disaster prevention and emergency management, although different dimensions of social capital may operate differently across emergency contexts. Structural social capital, embedded in social networks, enables individuals and organizations to access resources, information, and collaborative opportunities, and may be associated with improved preparedness and response efficiency [33]. Relational social capital, particularly trust, is widely regarded as an important factor influencing public willingness to participate in emergency prevention and control efforts. Individuals’ adoption of protective behaviors depends, to some extent, on their expectations of others’ actions as well as their level of trust in healthcare institutions. Studies by Wang et al. [34] and Puro and Kelly [35] indicate that trust-based relational social capital supports collaboration between healthcare workers and volunteers and is associated with more effective resource allocation and service delivery. Cognitive social capital contributes to the development of shared goals and behavioral norms. Jones and Faas [36] highlight its relevance in post-disaster recovery processes. Taken together, these findings suggest that different forms of social capital may be positively associated with the health emergency capacity of primary healthcare institutions.

Therefore, we propose the following hypotheses:

H2: Social capital positively affects the health emergency capacity of primary healthcare institutions.

H2a: Structural social capital positively affects the health emergency capacity of primary healthcare institutions.

H2b: Relational social capital positively affects the health emergency capacity of primary healthcare institutions.

H2c: Cognitive social capital positively affects the health emergency capacity of primary healthcare institutions.

### The impact of organizational culture on social capital

Cultural norms, as well as organizational goals and values, are likely to influence the form and function of social capital. Research by Malone and Kinnear [37] and Uekusa et al. [38] has shown that hared goals, stable norms, and a clear service orientation may encourage more frequent interaction, stronger trust, and more consistent expectations for collaboration both within the institution and with community actors. A thematic analysis of organizational culture within emergency management agencies suggests that when employees strongly identify with organizational goals and adhere to a unified set of values, both the internal network density and normative social relations are significantly strengthened [39]. Healthcare institutions that provide high-quality services while conveying a clear and consistent vision and ethical guidelines can cultivate trust in public health strategies at the community level, thereby fostering the development of cognitive social capital [40]. Moreover, by delivering high-quality services while articulating clear value orientations and behavioral norms, healthcare institutions can help build trust at the community level, which is associated with higher levels of cognitive social capital [41]. We propose the following hypotheses:

H3: Organizational culture positively affects social capital.

H3a: Organizational culture positively affects structural social capital.

H3b: Organizational culture positively affects relational social capital.

H3c: Organizational culture positively affects cognitive social capital.

### The mediating role of social capital between organizational culture and health emergency capacity

Social capital, as a fundamental resource trusted by individuals and society in emergency situations, plays an irreplaceable role in emergency management. Organizational culture enhances the cohesion and sense of public purpose within communities, thereby enriching and activating the social capital of communities where primary healthcare institutions are situated. Previous research has shown that, under the influence of cultural orientation and supportive policies, structural and relational social capital networks in remote and vulnerable areas have been significantly strengthened, contributing to a notable increase in the “adaptive resilience” of healthcare institutions in the aftermath of natural disasters [42].

By reinforcing shared values and behavioral norms, organizational culture is associated with the extent of an organization’s engagement at the community level and its cross-actor collaboration, thereby facilitating the development of social network connections and relating to emergency capacity [28]. Moreover, as important nodes within the community, primary healthcare institutions are embedded in trust-based relationships and normative constraints, which facilitate information flow and interactional coordination and are associated with improved emergency response outcomes [43]. Furthermore, several empirical studies provide additional support, indicating that structural and cognitive forms of social capital mediate the relationship between organizational culture and emergency performance outcomes [44]. Thus, it is evident that social capital acts as a conduit between organizational culture and emergency capabilities of primary healthcare institutions. Therefore, we propose the following hypotheses:

H4: Social capital mediates the relationship between organizational culture and the emergency capabilities of primary healthcare institutions.

H4a: Structural social capital mediates the relationship between organizational culture and the emergency capabilities of primary healthcare institutions.

H4b: Cognitive social capital mediates the relationship between organizational culture and the emergency capabilities of primary healthcare institutions.

H4c: Relational social capital mediates the relationship between organizational culture and the emergency capabilities of primary healthcare institutions.

**Fig 1:**
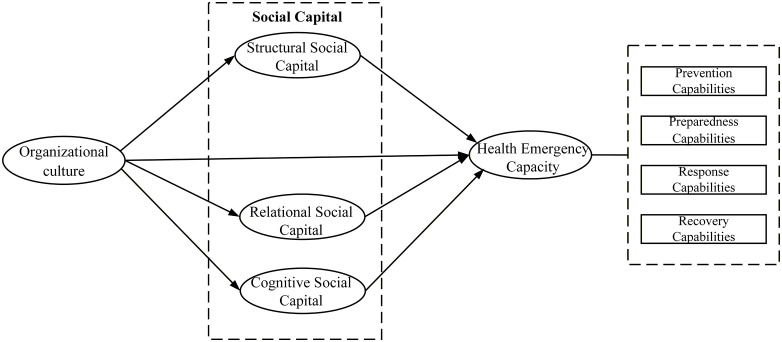
Research framework.

In conclusion, this study constructed a theoretical hypothesis model (Fig 1) with organizational culture of primary healthcare institutions as the independent variable, social capital as the mediating variable, and health emergency capacity as the dependent variable.

## Methods

### Participants and data collection

This study is based on the latest gross domestic product (GDP) rankings of 11 cities in Zhejiang Province, China, in 2022. The cities were categorized into four levels according to their economic status, considering regional economic indicators and public finance data. The first level includes Hangzhou and Ningbo, the two cities with a total GDP exceeding one trillion RMB. The second level comprises Wenzhou, Shaoxing, Jiaxing, and Taizhou, which have a GDP exceeding 500 billion RMB. The third level includes Jinhua and Huzhou with a GDP between 300 billion and 500 billion RMB. The fourth level comprises Quzhou, Lishui, and Zhoushan, which have a GDP not exceeding 200 billion RMB. Random sampling was used to select one city from each level: Hangzhou, Jiaxing, Huzhou, and Lishui. These cities exhibit considerable differences in the development of old neighborhoods and can provide a comprehensive reflection of the overall situation in Zhejiang Province. As the most economically developed city in Zhejiang Province, Hangzhou has relatively well-developed infrastructure in its old neighborhoods, ample medical resources, and strong emergency-management capabilities within primary healthcare institutions. Since the launch of the old neighborhood renovation program in 2019, Hangzhou has been ranked first for three consecutive years. The city focuses on integrated planning of public service facilities and community healthcare service networks, significantly enhancing the emergency response capabilities of grassroots communities and healthcare institutions. Jiaxing and Huzhou, while economically more developed, still encounter some deficiencies in infrastructure and public service resource allocation compared to Hangzhou. As of September 2022, Jiaxing had completed the renovation of 491 old neighborhoods, constructed 651,200 square meters of urban community service centers, and established 30 community health service centers [45]. By November 2022, Huzhou had included 89 neighborhoods in its renovation plan [46]. Lishui faces significant challenges in its old neighborhoods, including aging infrastructure and insufficient medical resources. The progress of its old neighborhood renovation has been relatively slow compared to the other three cities.

We randomly selected five primary healthcare institutions from the four cities chosen. Each primary healthcare institution employed simple random sampling to include approximately 55 healthcare professionals. Eligibility criteria for participating healthcare professionals included a minimum of six months of service at the survey unit, possession of professional qualifications, and voluntary participation in the study. Exclusion criteria encompassed current graduate students, visiting scholars, and doctors lacking understanding of or willingness to participate in the study objectives.

Sample size determination was guided by SEM principles. Thompson suggests a minimum ratio of 10 participants per measurement variable in SEM with ratios of 15:1 or 20:1 being preferable [47]. Given the inclusion of 15 measurement variables in this study, a theoretical minimum of 300 respondents was required. From March 1 to May 20, 2023, 1100 questionnaires were distributed to healthcare professionals. Of these, 983 were deemed valid, resulting in an effective response rate of 89.4%.

Over half the participants were under the age of 40, and three-quarters held an associate degree or higher. The primary professional groups represented were physicians (41.1%) and nurses (32.0%). Approximately 75% of the respondents held a professional title, and 72.5% had more than six years of work experience. Regarding institutional context, 72.6% of the respondents were employed at primary healthcare institutions located in ordinary communities, while 27.4% worked in older communities. Statistical analyses revealed significant differences in the public health emergency response capacity of primary healthcare institutions based on profession, community type, presence of hazard identification and risk assessment systems, implementation of a public health emergency accountability system, and perceived usefulness of intelligent tools. (See S1 Table).

### Measures

#### Measurement of organizational culture.

We defined organizational culture according to the framework proposed by Huang [48], who uses three items, such as “There is a common goal and mission among the members of your organization”.All items were rated on a 5-point Likert scale ranging from 1 (strongly disagree) to 5 (strongly agree), with higher scores indicating a better state of organizational culture in the primary healthcare institution. In this study, the overall Cronbach’s α coefficient for the scale was 0.969. The composite reliability (CR) value was 0.970. Convergent validity was assessed using the Average Variance Extracted (AVE), while discriminant validity was evaluated based on the Fornell-Larcker criterion, which compares the AVE of each construct with the squared correlations between constructs. The AVE for the latent variable construct was 0.915, and the square root of the AVE was 0.957. Item total correlation coefficients ranged from 0.971 to 0.982, indicating high internal reliability and satisfactory construct validity of the scale (See S2 and S3 Tables).

#### Measurement of social capital.

Social capital was measured based on the scale developed by Luo [49]. The scale comprises three dimensions of structural, relational, and cognitive social capital totaling 18 items. Structural social capital includes network interaction, network size, and community participation. Relational social capital encompasses community trust and reciprocity. Cognitive social capital is measured by three items, such as “Your organization has established a comprehensive volunteer management system.” All items were rated on a 5-point Likert scale ranging from 1 (strongly disagree) to 5 (strongly agree), with higher scores indicating greater social capital in the primary healthcare institution. In this study, Cronbach’s α coefficient for the scale was 0.960, and Cronbach’s α for the seven dimensions ranged from 0.890 to 0.976. The CR values were all greater than 0.7, and the AVE for the latent variables ranged from 0.811 to 0.976. The square root of the AVE was between 0.868 and 0.917. The correlation coefficients between each item and the total score of the scale ranged from 0.777 to 0.869, indicating that the scale has good reliability and validity (S2 and S3 Tables).

#### Measurement of health emergency capacity.

The measurement of health emergency capacity was based on the disaster emergency capacity evaluation scale developed by Lin [8]. This scale includes four dimensions: emergency prevention, preparedness, response, and post-disaster recovery. Items were rated on a 5-point Likert scale ranging from 1 (strongly disagree) to 5 (strongly agree), with higher scores indicating stronger health emergency capacity of the primary healthcare institution. In this study, the overall Cronbach’s α coefficient for the scale was 0.983. Cronbach’s α for the four dimensions ranged from 0.960 to 0.985. The CR values were all greater than 0.7, and the AVE for the latent variables ranged from 0.836 to 0.930. The square root of the AVE ranged from 0.931 to 0.965. The correlation coefficients between each item and the total score of the scale ranged from 0.846 to 0.953, indicating that the scale has good reliability and validity (See S2 and S3 Tables).

#### General information survey.

The first section covered demographic variables, including sex (male and female), age (18–30 years, 31–40 years, 41–50 years, 51–60 years, 61 years and above), political affiliation (the masses, Communist Youth League member, party activist, preparatory party members, Communist Party member), educational level (junior high school or below, high school, associate degree, bachelor’s degree or above), marital status (single, married, divorced, other), position (doctor, nurse, medical technician, prevention, administration, other), professional title (none, junior, intermediate, senior), years of service (less than 1 year, 1–3 years, 4–6 years, more than 6 years), and type of community they work in (ordinary community, old community). The second section covered community-management variables, including the presence of hazard identification and risk assessment management systems (yes, no, don’t know), accountability systems for public health emergencies (yes, no, don’t know), and the extent of the usefulness of smart tools (very helpful, helpful, average).

Before data collection, a pilot survey was conducted to gather preliminary data and identify potential issues in the questionnaire design. Based on the results of the pilot survey, we revised the questionnaire and finalized its content. The questionnaires were distributed by graduate students with substantial experience in field surveys. Prior to the formal survey, the graduate students underwent intensive training sessions covering survey standards, methodologies, and data collection and processing procedures to ensure consistency and reliability throughout the survey process. After the survey was completed, all questionnaires were carefully reviewed, and any missing information was promptly addressed to ensure data completeness. Finally, each questionnaire was assigned a unique identifier and double-entered by the researchers to ensure accuracy.

### Ethical considerations

This study was approved by the Ethics Review Committee of Hangzhou Normal University (approval number 2022−1121). Before the questionnaire surveys, we briefly described the purpose of the study to the participants and obtained their written informed consent. All procedures were performed in accordance with the ethical principles of the Committee on Human Experimentation and Declaration of Helsinki.

### Statistical analyses

Statistical analyses were conducted using SPSS 26.0 and AMOS 26.0. We used SPSS 26.0 to conduct frequency and proportion analyses on the data. By conducting a two-tailed test of Pearson correlation analysis, we could examine the correlation coefficients between organizational culture, social capital, and healthcare emergency capabilities. SEM was conducted using AMOS 26.0 to test the impact of organizational culture and social capital on health emergency capability, and the bootstrap method was applied to test the mediating effect of social capital. Finally, multi-group SEM analysis was performed using community type as the grouping variable to evaluate the measurement invariance of the proposed model across ordinary and older community groups, facilitating cross-group comparisons. SEM is a multivariate statistical technique that utilizes the covariance matrix of variables for both factor and path analysis. It is designed to investigate the latent relationships among multiple variables and to provide a comprehensive analysis of their interactive mechanisms. SEM is extensively used in disciplines such as psychology and sociology, making it an appropriate choice for the present study.

## Results

### Correlation analysis among key variables

The organizational culture of primary healthcare institutions was positively correlated with structural, relational, and cognitive social capital and with health emergency capability with correlation coefficients ranging from 0.676 to 0.833 (all *P* < 0.01). The highest correlation was observed between organizational culture and cognitive social capital (r = 0.833, *P* < 0.01). Additionally, structural, relational, and cognitive social capital were all positively correlated with health emergency capability with correlation coefficients ranging from 0.667 to 0.769 (all *P* < 0.01). Among these, the strongest correlation was observed between relational social capital and health emergency capability (r = 0.769, *P* < 0.01) (Table 1).

**Table 1:** Means, standard deviations, correlation coefficients of the variables.

Variable	Mean	SD	1	2	3	4	5
1 Organizational culture	4.24	0.56	1				
2 Structural social capital	4.13	0.56	0.676**	1			
3 Relational social capital	4.22	0.52	0.779**	0.742**	1		
4 Cognitive social capital	4.19	0.60	0.833**	0.643**	0.746**	1	
5 Health emergency capability	4.32	0.51	0.818**	0.667**	0.769**	0.766**	1

Note: *** means *P* < 0.001; **means *P* < 0.01; *means *P* < 0.05. SD = standard deviation.

### Comprehensive model analysis

#### Model construction and fit.

**Fig 2:**
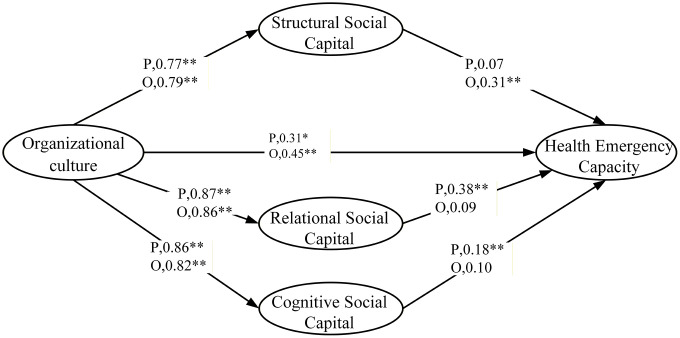
Model of health emergency capacity of primary medical institutions.

In this study, an SEM was constructed with organizational culture as the independent variable; health emergency capability as the dependent variable; and structural, cognitive, and relational social capital as the mediating variables (Fig 2). The model fitting results indicate that all paths were significant, yet the fit indices did not reach ideal values. An excessively large sample size may lead to poor overall model fit; therefore, this study utilized the Bollen-Stine p-value correction method to refine the model [50,51]. After 2000 bootstrap samples, the corrected Bollen-Stine bootstrap p-value was found to be 0.000 with a χ2/df ratio of 1.683 falling within the [1,3] interval. Additionally, goodness of fit indices (goodness of fit index, adjusted goodness of fit index, normal fit index, comparative fit index, Tucker Lewis index) exceeded the recommended threshold of 0.90, and the root mean square of approximation and its 90% confidence interval were less than 0.08, suggesting an acceptable level of approximation error, indicating that the model exhibits good overall fit (Table 2).

**Table 2:** Fitting results of structural equation model.

Fitting index	Fitting standard	Initial model	Modified model
*χ*^2^/*df*	1 < *χ*^2^/*df* < 3 Good	9.032	2.800
RMSEA (90%CI)	<0.08 Good	0.090	0.043
GFI	>0.9 Good	0.908	0.988
AGFI	>0.9 Good	0.866	0.978
NFI	>0.9 Good	0.962	0.988
CFI	>0.9 Good	0.966	0.992
TLI	>0.9 Good	0.956	0.990

Note: RMSEA = root mean square error of approximation; GFI = goodness-of-fit index; AGFI = adjusted goodness of fit index; NFI = normed fit index; CFI = comparative fit index; TLI = Tucker Lewis index; CI = confidence interval.

#### Path analysis.

Based on the standardized path coefficients, it is evident that organizational culture, structural social capital, relational social capital, and cognitive social capital have positive effects on the health emergency capabilities of primary healthcare institutions. The standardized path coefficients were 0.365, 0.131, 0.297, and 0.156, respectively (p < 0.001), supporting hypotheses H1, H2a, H2b, and H2c. Furthermore, organizational culture positively influenced structural social capital, relational social capital, and cognitive social capital, with standardized path coefficients of 0.776, 0.866, and 0.850, respectively; thus, hypotheses H3a, H3b, and H3c were supported (Table 3).

**Table 3:** The influence path coefficients and verification hypothesis of health emergency capacity.

Path	Unstandardized coefficient	Standardized coefficient	SE	*P*	Supported hypotheses
Organizational culture → Health emergency capabilities	0.330	0.365	0.056	<0.001	H1
Structural social capital → Health emergency capabilities	0.142	0.131	0.037	<0.001	H2a
Relational social capital → Health emergency capabilities	0.316	0.297	0.049	<0.001	H2b
Cognitive social capital → Health emergency capabilities	0.131	0.156	0.029	<0.001	H2c
Organizational culture → Structural social capital	0.647	0.776	0.029	<0.001	H3a
Organizational culture → Relational social capital	0.736	0.866	0.023	<0.001	H3b
Organizational culture → Cognitive social capital	0.916	0.850	0.023	<0.001	H3c

Note: SE = standard error; H = hypothesis.

#### Mediating effect.

The mediating effects of health emergency capabilities in primary healthcare institutions were examined using the bootstrap method with a confidence interval set at 95%. Analysis was conducted through maximum likelihood estimation over 2000 samples. Organizational culture had a significant direct effect on health emergency capabilities with an effect size of 0.365, and the confidence interval analysis excluded 0. Moreover, organizational culture exerted significant indirect effects on health emergency capabilities through structural social capital, cognitive social capital, and relational social capital, with indirect effect sizes of 0.101, 0.257, and 0.133, respectively. These findings suggest that these forms of social capital partially mediate the relationship in the model, supporting hypotheses H4a, H4b, and H4c (Table 4).

**Table 4:** Examination of the mediating effects with the bootstrap method (standardized coefficients).

Variable relationship	Effect type	Effect value	Bias-corrected 95%CI		Percentile 95%CI	
			Lower	Upper	Lower	Upper
Organizational culture → Structural social capital → Health emergency capabilities	Total effect	0.466	0.281	0.642	0.267	0.633
	Direct effect	0.365	0.173	0.558	0.162	0.539
	Indirect effect	0.101	0.029	0.201	0.024	0.195
Organizational culture → Relational social capital → Health emergency capabilities	Total effect	0.622	0.488	0.757	0.488	0.755
	Direct effect	0.365	0.173	0.558	0.162	0.539
	Indirect effect	0.257	0.115	0.439	0.115	0.439
Organizational culture → Cognitive social capital → Health emergency capabilities	Total effect	0.497	0.305	0.658	0.299	0.653
	Direct effect	0.365	0.173	0.558	0.162	0.539
	Indirect effect	0.133	0.036	0.228	0.035	0.227

Note: CI = confidence interval.

### Multi-group SEM analysis

Using community type as a grouping variable, this study conducted multi-group path analysis, testing from the most lenient model to the most stringent restricted model. The first step involved developing an unconstrained model, which imposes no equality constraints on the parameters between the ordinary and old community groups. In the second step, equality constraints were sequentially applied to the parameters of the measurement models for the two groups, resulting in the following models: the measurement-weighted model (equal factor loadings), the structural-weighted model (equal path coefficients), the structural covariance model (equal structural covariances), the structural error covariance model (equal error term covariances), and the measurement residual model (equal measurement error variances). The non-significant changes in chi-square values across the constrained models indicate that imposing equality constraints on the corresponding parameters between the two groups is justifiable, supporting the model’s invariance. The third step involved constructing the final model, incorporating the equality constraints identified in the second step into the unconstrained model. The bias-corrected bootstrap method was then employed to estimate the standardized direct, indirect, and total effects for both ordinary and old community groups.

Results indicated that the fit indices for the unconstrained model M1, measurement weights model M2, structural weights model M3, structural covariances model M4, structural residuals model M5, and measurement residuals model M6 were generally acceptable. Based on fitness and model invariance tests through multi-group comparisons, the p-values for the invariance tests of structural weights, structural residuals, and measurement residuals models were all less than 0.05. However, according to Little, comparing partially constrained and unconstrained models yielded p-values less than 0.05, and if △CFI is less than or equal to 0.01 and △TLI is less than 0.05, the hypothesis of no difference in models can be accepted [52]. In this study, the absolute values of changes in model fit indices were less than 0.01, indicating measurement model equivalence across both community types (results are shown in S4a, S4b in S4 and S5 Tables).

**Fig 3:**
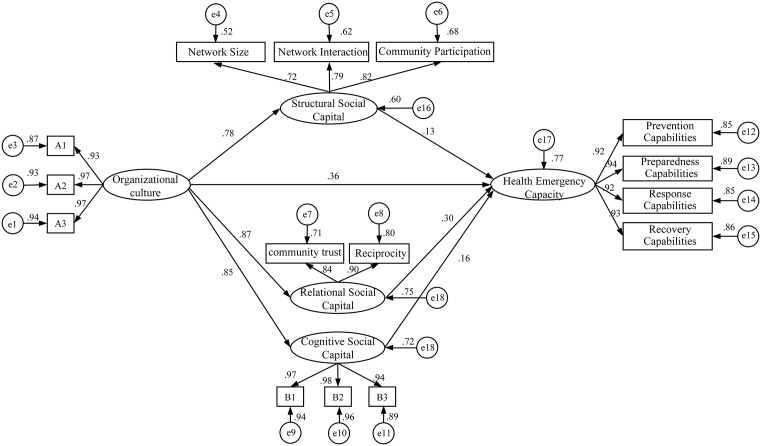
Multi-group structural equation model of primary medical institutions’ health emergency capacity in ordinary and older communities. Note: Ordinary community group: P; old community group: O.

As shown in Table 5, employing bias-corrected bootstrap tests, this study compared the impacts of organizational culture and social capital on the health emergency capacity of primary healthcare institutions between ordinary and older communities. The results indicated that organizational culture had significant direct effects on health emergency capacity in both community types with standardized path coefficients of 0.314 and 0.453. Organizational culture indirectly affected the health emergency capacity of primary healthcare institutions in ordinary communities through relational social capital and cognitive social capital, with indirect effect values of 0.428 and 0.159, respectively. In the old community group, organizational culture affected the health emergency capacity of primary healthcare institutions through structural social capital with an indirect effect value of 0.349 (Fig 3). Therefore, in ordinary communities, the direct impact of organizational culture on health emergency capacity was smaller than its indirect impact, whereas, in older communities, the direct impact was greater than the indirect impact. Furthermore, utilizing the CR index to compare the differences in structural path coefficients between different community categories revealed significant disparities. The coefficient from structural social capital to health emergency capacity was significantly higher in the old community group (CR = 3.081, *P* < 0.05). Conversely, the coefficient from relational social capital to health emergency capacity was significantly higher in the ordinary community group (CR = −2.833, *P* < 0.05). The path coefficients from organizational culture to health emergency capacity did not differ significantly between different community groups (CR = 1.075, *P* > 0.05). These findings partially confirmed hypotheses H3 and H4 (Fig 3).

**Table 5:** Comparison of the impact of each action path of health emergency capacity of primary medical institutions in ordinary communities and older communities.

Path	Ordinary community						Old community						CR
	Direct effect		Indirect effect		Total effect		Direct effect		Indirect effect		Total effect		
	B	SE	B	SE	B	SE	B	SE	B	SE	B	SE	
CA → HEC	0.314*	0.121					0.453**	0.145					1.075
CA → SSA	0.771**	0.043					0.788**	0.051					−1.158
CA → RSC	0.866**	0.031					0.854**	0.041					−1.111
CA → CSC	0.862**	0.019					0.817**	0.036					1.318
SSA → HEC	0.067	0.061					0.311**	0.101					3.081
RSC → HEC	0.383**	0.123					0.086	0.130					−2.833
CSC → HEC	0.184**	0.07					0.096	0.095					−1.433
CA → SSA → HEC			0.052	0.049					0.245*	0.085			
CA → RSC → HEC			0.331**	0.113					0.074	0.112			
CA → CSC → HEC			0.158**	0.061					0.078	0.079			
Ind (CA → HEC)			0.541**	0.114					0.397**	0.138			
Tot (CA → HEC)					0.855**	0.019					0.85**	0.035	

Note: CA = organizational culture; HEC = Health emergency capabilities; SSA = Structural social capital; RSC = Relational social capital; CSC = Cognitive social capital; Ind = total indirect effect; Tot = total effect; SE = standard error; CR describes the degree of difference in the path coefficients of different groups; if the absolute value of CR is greater than 1.96, it means that the path is significantly different.

## Discussion

### Impact of organizational culture and social capital on the health emergency capacity of primary healthcare institutions

The findings of this study indicate that organizational culture in primary healthcare institutions has a positive effect on health emergency capacity, with this influence manifested in particular through concrete practices such as culture walls and the implementation of Socialist Core Values. This result is consistent with the findings reported by Wolf-Fordham [53]. Specifically, higher levels of organizational culture strengthen ideological guidance, cultivate collective agency, and ultimately enhance institutions’ emergency response capacity. As a tangible expression of organizational culture, culture walls can deepen staff members’ identification with shared values, thereby reinforcing collective action and facilitating a more efficient emergency response [54]. Furthermore, in high-stress environments, a climate of solidarity and mutual support fostered by organizational culture can help alleviate and buffer healthcare workers’ emotional strain, sustain stable professional conduct and willingness to collaborate, and thereby enhance team coordination and decision-making efficiency during emergency situations [55].

Furthermore, this study indicates that social capital also positively influences the health emergency capacity of primary healthcare institutions, in line with the conclusions of Derose and Varda [56]. Greater stockpiles of social capital enhance the emergency capabilities of service-providing organizations such as hospitals. Among the different types of social capital, relational social capital exhibits the strongest effect, consistent with Yang et al. [57]. This finding partially supports the “strength of strong ties” hypothesis, suggesting that in the Chinese context, strong ties characterized by trust and obligation are often more influential than network size, particularly in accessing high-value and high-cost social resources [58]. Residents’ trust and high compliance significantly improve emergency response efficiency. In addition, Hall et al. [59] further confirmed the positive role of cognitive social capital in enhancing health emergency capacity. Standardized emergency protocols and volunteer management systems are associated with enhanced community governance practices and higher levels of institutional emergency preparedness. Interestingly, structural social capital exerts the least significant effect on health emergency capacity. This may be because multi-stakeholder networks can increase decision-making complexity, potentially reducing the efficiency of emergency responses. Based on these findings, this study empirically supports the strategy of enhancing health emergency capacity through the optimization of social capital structures.

### Mediating role of social capital

The results of this study indicate that social capital exhibits an indirect pathway between organizational culture and health emergency capacity, which is consistent with prior findings [60]. Among the different dimensions, relational social capital shows the strongest indirect effect, a pattern also reported by Abdullah et al. [61]. Specifically, in contexts characterized by increased uncertainty and risk, clearer value orientations and organizational goals embedded in organizational culture are positively associated with higher levels of trust and cohesion among community residents, and are further related to improved coordination among emergency-related stakeholders. Cognitive social capital also demonstrates a relatively notable indirect effect along this pathway. Standardized emergency protocols and volunteer management systems are associated with greater normative consistency and may contribute to more consistent policy implementation, which is reflected in the precision and effectiveness of emergency responses in primary healthcare institutions. In contrast, structural social capital shows the weakest indirect effect between organizational culture and health emergency preparedness, which differs from some previous studies [62]. This discrepancy may be related to changes in social structures and the commercialization of residential spaces, which may have increased the homogeneity and closure of intra-community social networks; however, this interpretation requires further empirical validation.

### Analysis of differences in health emergency capability of primary healthcare institutions among different community types

This study reveals significant differences between ordinary and older communities in terms of organizational culture, social capital, and health emergency capacity. In ordinary communities, the positive impact of relational social capital on health emergency capacity was stronger than in older communities. This finding is consistent with the conclusions of Lee [63], who noted that ordinary communities typically have higher socioeconomic status and well-established community organizational structures, which facilitate the accumulation of relational social capital, such as trust and reciprocal networks among community members. In such contexts, residents in general communities actively participate in public health initiatives, enhancing the efficiency of prevention and control efforts. Equally important, in older communities, structural social capital plays a more significant role in enhancing health emergency capacity. This may be because, in emergency situations, structural social capital often manifests through connections and collaborations between the community, primary healthcare institutions, and other entities such as government agencies and non-government organizations. Resource-scarce older communities tend to rely more on these bridging or vertical networks to secure disaster relief supplies and technical support. Previous studies have indicated that structural social capital is a critical mechanism for mobilizing external resources to the community [64]. Notably, our findings indicate that the impact of organizational culture on health emergency capacity does not reveal community-type differences. This can be explained by the sense of shared destiny in emergency situations, which drives both residents and healthcare professionals to transcend traditional community boundaries, forming a transcendent collective identity centered around public health goals. Consequently, the value-driven effect of organizational culture remains consistent across different community types [65]. This finding suggests that, although differences exist across communities in terms of social capital composition and resource distribution, collective actions shaped by organizational culture may extend beyond physical community boundaries and are associated with enhanced coordination and emergency response capacity during public health crises. Therefore, understanding these differences is important for developing context-sensitive emergency strategies and for improving the health emergency capacity of primary healthcare institutions across diverse community settings.

## Summarize

### Conclusion

This study focuses on primary healthcare institutions and differs from much of the existing literature that emphasizes large hospitals or macro-level emergency systems. From the perspective of community-based, grassroots healthcare service settings, it examines the path relationships among variables associated with health emergency capacity. This perspective provides additional empirical evidence that contributes to the literature on emergency preparedness at the primary healthcare level. The results indicate that organizational culture in primary healthcare institutions is positively associated with health emergency capacity both directly and indirectly through different forms of social capital, with the pathway via relational social capital exhibiting the strongest effect. Multi-group analysis further reveals heterogeneity across community contexts: in ordinary communities, relational and cognitive social capital show significant associations with health emergency capacity, whereas in resource-constrained older communities, the mediating role of structural social capital is more pronounced. These findings suggest that improvements in the emergency capacity of primary healthcare institutions are not only related to resource inputs but are also associated with organizational culture and social capital. From a practical perspective, the results provide a complementary “soft-capacity” lens for health authorities and institutional managers, highlighting the potential value of fostering shared values and internal cohesion, alongside the targeted development of social capital, to support the effective utilization of emergency resources and improve emergency capacity at the primary healthcare level.

### Theoretical contribution

This study demonstrated the significant influence of organizational culture and social capital on health emergency capacity, offering several theoretical contributions. By incorporating organizational culture into the analytical framework of emergency preparedness in primary healthcare institutions, this study examines its relationship with health emergency capacity, thereby extending existing perspectives that have predominantly focused on institutional arrangements, resources, and technical capabilities. Moreover, by comparing the differential roles of various types of social capital within the pathways linking organizational culture and emergency capacity, this study further extends the empirical research on the relationship between organizational culture and social capital. A further innovation lies in accounting for heterogeneity at the community level: we systematically tested how organizational culture and the three forms of social capital differentially affect health emergency capacity across community contexts. This community-type perspective—seldom employed in previous research—offers a novel lens for cultivating organizational culture and social capital in primary healthcare institutions according to local community characteristics.

### Managerial implications

(1) The findings suggest that primary healthcare institutions may benefit from strengthening shared mission orientation, value alignment, and clear role expectations as part of emergency preparedness work. Rather than treating organizational culture as a symbolic activity alone, institutional managers could integrate value communication with emergency drills, staff training, and cross-departmental coordination procedures. Such activities may help ensure that cultural messaging is connected to practical preparedness routines.
(2) Primary healthcare institutions can enhance emergency capacity by optimizing different types of social capital. Regarding structural social capital, regional and grassroots social organizations should be encouraged to develop in coordination with primary healthcare institutions. By promoting community network coordination mechanisms and building resource-sharing platforms, multiple actors can achieve information symmetry and task collaboration, improving the integration efficiency of structural social capital and, consequently, enhancing emergency capacity. Regarding relational social capital, institutions should strengthen interaction mechanisms with residents, improve online feedback channels, enhance institutional credibility, and consolidate the foundation of trust. The primary beneficiaries of this work are residents within the service catchment area; by building robust clinician–patient trust, it helps ensure smooth public cooperation and community mobilization during emergencies. Regarding cognitive social capital, community platforms can be used to establish standardized systems for volunteer recruitment, training, certification, and incentives. Clear emergency-duty manuals and cross-departmental collaboration procedures should be developed to reinforce normative consensus and institutional compliance. These measures directly respond to the national call to strengthen an emergency response system that integrates specialized and routine capacities, and they contribute to more standardized and efficient emergency responses at the primary level.
(3) Communities can strategically strengthen social capital according to their characteristics. In ordinary communities, their well-developed social networks and resource advantages should be fully utilized to tightly integrate organizational culture with relational social capital, thereby building trust networks and volunteer service systems. In older communities, structural social capital should be reinforced, coordination mechanisms with government agencies and social organizations should be improved to ensure timely provision of emergency supplies and technical support, and, by leveraging the community’s historical culture and acquaintance-based social structures, cultural incentive strategies should be organically embedded into community governance to maximize the direct mobilizing effect of organizational culture. This should be treated as a priority in emergency-capacity development programs for aged communities. In addition, the distinctive local heritage and acquaintance-based social structure often found in general communities create favorable conditions for implementing culture-based motivational strategies. By leveraging cultural resources to strengthen residents’ cohesion and willingness to cooperate, such strategies can effectively promote the co-development of emergency management and community governance.

### Research limitations and prospects

This study presents the following limitations. First, this study adopts a cross-sectional design, which captures data at a single point in time and limits the ability to establish temporal ordering or infer causal pathways among variables. Therefore, although structural equation modeling was employed to examine the path relationships among variables, the results should be interpreted as reflecting statistical associations and model-implied pathways rather than definitive causal relationships. Future research could employ longitudinal or intervention-based designs to further investigate temporal dynamics and potential causal pathways among these variables. Second, the sample in this study was primarily drawn from four cities in Zhejiang Province, a relatively developed region in eastern China, which limits its representativeness. The findings mainly reflect conditions in economically developed areas and do not cover less-developed regions, thereby constraining the generalizability of the results to other underdeveloped areas or countries. Future studies should expand the sample to include less-developed regions in central and western China, and even internationally comparable samples, to enhance the representativeness and generalizability of the findings. Furthermore, this study assesses health emergency capacity by surveying healthcare providers’ perceptions of their organizations’ capabilities, which relies on subjective perceptions rather than objective measurement. Although such perceptions can reflect providers’ understanding of institutional readiness, they may also be influenced by factors such as organizational culture and social capital. Accordingly, future research could incorporate a broad set of objective indicators—for example, actual performance in emergency drills, allocation of emergency resources, and outcomes of responses to public health incidents—to provide a more comprehensive assessment of health emergency capacity. Finally, although this study employed a multi-group structural equation modeling approach to analyze the effects of different community types on health emergency capacity, it did not consider the potential roles of healthcare workers’ gender, age, and other demographic factors. Future research could introduce interaction terms or conduct multi-group analyses based on age, gender, or professional groupings to more accurately estimate the independent effects of profession and other core variables, reduce confounding bias, and improve both the internal validity and generalizability of the results.

## Supporting information

S1 Table Descriptive statistics of the sample (N = 983). (DOCX)

S2 Table Variable measurement, factor analysis results, and reliability coefficient. (DOCX)

S3 Table Distinguishing the validity of the organizational culture, social capital, and health emergency response competency measurement scales. (DOCX)

S4 Table S4a and S4b Tables. Multi-group comparison of adaptation, and multi-group comparison of adaptation. (DOCX)

S5 Table Invariance test. (DOCX)

S1 Data Raw data. (XLSX)
