# Supplementary material for: Organizational culture, social capital, and emergency capacity in primary healthcare institutions: A cross-sectional structural equation modeling study comparing ordinary and older communities
Source: PLoS One. 2026 Jun 30;21(6):e0351875. doi: 10.1371/journal.pone.0351875 (PMC13318035; doi:10.1371/journal.pone.0351875)
Supplement: S1 Table — (DOCX) [file pone.0351875.s001.docx]

**Table S1. Descriptive Statistics of the Sample (N = 983).**

| **Variable** | **Categorization** | **Frequency (N)** | **Composition (%)** | **F/t** | ***P*** |
| --- | --- | --- | --- | --- | --- |
| Sex | Male | 216 | 22 | 2.241 | 0.349 |
|  | Female | 767 | 78 |  |  |
| Age | 18–30 years | 304 | 30.9 | 0.721 | 0.577 |
|  | 31–40 years | 369 | 37.5 |  |  |
|  | 18–30 years | 225 | 22.9 |  |  |
|  | 42–50 years | 73 | 7.4 |  |  |
|  | 51–60 years | 12 | 1.2 |  |  |
|  | 61 years and above | 304 | 30.9 |  |  |
| Educational level | Junior high school or below | 17 | 1.7 | 0.527 | 0.716 |
|  | High school | 47 | 4.8 |  |  |
|  | Associate degree | 251 | 25.5 |  |  |
|  | Bachelor’s degree or above | 668 | 68 |  |  |
| Marital status | Single | 181 | 18.4 | 0.050 | 0.985 |
|  | Married | 784 | 79.8 |  |  |
|  | Divorced and other | 18 | 1.8 |  |  |
| Position | Doctor | 404 | 41.1 | 3.222 | 0.009 |
|  | Nurse | 315 | 32 |  |  |
|  | Medical technician | 102 | 10.4 |  |  |
|  | Prevention | 88 | 9 |  |  |
|  | Administration | 30 | 3.1 |  |  |
|  | Other | 44 | 4.5 |  |  |
| Professional title | None | 125 | 12.7 | 0.165 | 0.920 |
|  | Junior | 484 | 49.2 |  |  |
|  | Intermediate | 286 | 29.1 |  |  |
|  | Senior | 88 | 9 |  |  |
| Years of service | Less than 1 year | 28 | 2.8 | 0.540 | 0.655 |
|  | 1–3 years | 117 | 11.9 |  |  |
|  | 4–6 years | 125 | 12.7 |  |  |
|  | More than 6 years | 713 | 72.5 |  |  |
| The type of community you work in | Ordinary community | 714 | 72.6 | 0.789 | 0.015 |
|  | Older community | 269 | 27.4 |  |  |
| The presence of hazard identification and risk assessment management systems | Yes | 788 | 80.2 | 24.334 | <0.001 |
|  | No | 74 | 7.5 |  |  |
|  | Don’t know | 121 | 12.3 |  |  |
| Accountability systems for public health emergencies | Yes | 865 | 88 | 30.239 | <0.001 |
|  | No | 47 | 4.8 |  |  |
|  | Don’t know | 71 | 7.2 |  |  |
| The extent of the usefulness of smart tools | Very helpful | 763 | 77.6 | 43.787 | <0.001 |
|  | Helpful | 199 | 20.2 |  |  |
|  | Average | 19 | 1.9 |  |  |
