# Supplementary material for: Organizational culture, social capital, and emergency capacity in primary healthcare institutions: A cross-sectional structural equation modeling study comparing ordinary and older communities
Source: PLoS One. 2026 Jun 30;21(6):e0351875. doi: 10.1371/journal.pone.0351875 (PMC13318035; doi:10.1371/journal.pone.0351875)
Supplement: S2 Table — (DOCX) [file pone.0351875.s002.docx]

**S2 Table.**

| **Construct** | **Dimension** | **Measurement items** | **Load** | **Cronbach’s α** | **CR** | **AVE** | **Correlation coefficient** |
| --- | --- | --- | --- | --- | --- | --- | --- |
| Organizational culture | | There is a common goal and mission among the members of your organization. | 0.927 | 0.969 | 0.970 | 0.915 | 0.971 |
|  |  | Your organization has a corresponding culture wall to reflect the organization’s vision and cultural characteristic. | 0.970 |  |  |  | 0.981 |
|  |  | Your organization deeply implements socialist core values ​​in organizational activities under daily affairs. | 0.972 |  |  |  | 0.982 |
| Structural social capital | Network interaction | Strong ties between members of your organization. | 0.891 | 0.940 | 0.941 | 0.841 | 0.777 |
|  |  | Harmonious interpersonal relationships among members of your organization. | 0.946 |  |  |  | 0.781 |
|  |  | Communication between members of your organization is smooth. | 0.913 |  |  |  | 0.777 |
|  | Network size | Higher number of members (15 and more) in a collaborative relationship between your organization and other organizations. | 0.964 | 0.927 | 0.933 | 0.825 | 0.814 |
|  |  | High number of members with close ties between your organization and other organizations (15 and more). | 0.969 |  |  |  | 0.808 |
|  |  | Your organization has a high total number of volunteers (15 or more). | 0.778 |  |  |  | 0.769 |
|  | Community participation | Your organization and community residents are able to work together to prevent and control outbreaks or other health emergency activities. | 0.926 | 0.934 | 0.937 | 0.834 | 0.834 |
|  |  | In public health emergencies, the majority of Party members play a pioneering and exemplary role, becoming an important force in organizing and mobilizing the masses and enhancing the social participation network. | 0.939 |  |  |  | 0.827 |
|  |  | You believe that cooperation in the community is very important when public health emergencies occur. | 0.868 |  |  |  | 0.835 |
| Relational social capital | Community trust | You choose to believe other members of the community when they tell you something unexpected will happen. | 0.849 | 0.931 | 0.928 | 0.811 | 0.821 |
|  |  | When things get too busy in primary care, you choose to ask other organization members for help. | 0.924 |  |  |  | 0.843 |
|  |  | Seek advice from other organization members on matters that cannot be resolved. | 0.947 |  |  |  | 0.869 |
|  | Reciprocity | During public health emergencies, you often help other organization members and residents in the community. | 0.893 | 0.890 | 0.901 | 0.754 | 0.802 |
|  |  | During public health emergencies, other organizations in the community often provide you with help and guidance. | 0.927 |  |  |  | 0.835 |
|  |  | You believe that emergency mutual aid in the community is very important during a public health emergency outbreak. | 0.777 |  |  |  | 0.855 |
| Cognitive social capital | | Your organization has robust oversight mechanisms and accountability systems for failures to act. | 0.969 | 0.976 | 0.976 | 0.931 | 0.844 |
|  |  | Your organization has a well-established emergency assessment system in place. | 0.983 |  |  |  | 0.847 |
|  |  | Your organization has established a comprehensive volunteer management system. | 0.943 |  |  |  | 0.838 |
| Health emergency capabilities | Prevention capabilities | The organization has well-established rules and regulations for health emergencies. | 0.949 | 0.976 | 0.976 | 0.892 | 0.928 |
|  |  | The organization is engaged in explaining and disseminating knowledge of epidemic prevention policies to residents through online and offline media. | 0.957 |  |  |  | 0.928 |
|  |  | The organization has a well-established public health emergency information system portal. | 0.958 |  |  |  | 0.922 |
|  |  | The organization has taken the initiative to give full play to the advantages of Chinese medicine in preventing and treating future diseases and has promoted Chinese medicine health care methods among the residents. | 0.939 |  |  |  | 0.930 |
|  |  | The organization has a well-established monitoring and early warning system. | 0.918 |  |  |  | 0.911 |
|  | Preparedness  capabilities | The organization conducts regular health emergency drills. | 0.903 | 0.960 | 0.962 | 0.836 | 0.916 |
|  |  | The organization has an adequate public health talent pool. | 0.816 |  |  |  | 0.846 |
|  |  | The organization has established an outpatient department epidemic prevention and control team to improve the organizational structure. | 0.963 |  |  |  | 0.953 |
|  |  | The organization has sufficient health emergency supplies. | 0.953 |  |  |  | 0.950 |
|  |  | The organization focuses on strengthening the training of family doctors on emergency knowledge such as infectious diseases and psychological intervention. | 0.929 |  |  |  | 0.939 |
|  | Response capabilities | The organization actively implements pre-screening and triage and finds suspected patients and makes timely referrals. | 0.957 | 0.968 | 0.970 | 0.867 | 0.941 |
|  |  | The organization is responsible for disinfecting and disinfesting suspected or confirmed cases in the community by visiting people’s homes in person. | 0.931 |  |  |  | 0.914 |
|  |  | The host organization uses big data to locate residents and international students returning from high-risk areas and arranges for them to be admitted to a designated isolation site for quarantine observation. | 0.959 |  |  |  | 0.931 |
|  |  | The organization is able to implement “Internet +” medical services, through the family doctor to the contracted residents of the epidemic information conveyed and online order to guide the medical order. | 0.942 |  |  |  | 0.929 |
|  |  | The host organization has a robust volunteer system that ensures the supply and distribution of human resources and reduces the pressure on medical staff. | 0.863 |  |  |  | 0.898 |
|  | Recovery capabilities | The organization will carry out a thematic evaluation of the effectiveness of the handling of public health emergencies and develop a program to improve the system. | 0.957 | 0.985 | 0.985 | 0.930 | 0.945 |
|  |  | The organization is able to guide the normal operation of the order of medical care after a public health emergency. | 0.981 |  |  |  | 0.955 |
|  |  | The organization will formulate a targeted post-disaster recovery and reconstruction plan based on the damage assessment and actively implement it. | 0.981 |  |  |  | 0.952 |
|  |  | The organization focuses on strengthening psychological support and guidance for patients and families to avoid excessive anxiety and panic. | 0.967 |  |  |  | 0.941 |
|  |  | The host organization will provide appropriate psychological care and career development incentives to primary care staff. | 0.936 |  |  |  | 0.916 |

Note: AVE: average variance extracted; CR = composite reliability
