# Supplementary material for: Organizational culture, social capital, and emergency capacity in primary healthcare institutions: A cross-sectional structural equation modeling study comparing ordinary and older communities
Source: PLoS One. 2026 Jun 30;21(6):e0351875. doi: 10.1371/journal.pone.0351875 (PMC13318035; doi:10.1371/journal.pone.0351875)
Supplement: S3 Table — (DOCX) [file pone.0351875.s003.docx]

**S3 Table.**

|  | **AVE** | **1** | **2** | **3** | **4** | **5** | **6** | **7** | **8** | **9** | **10** | **11** |
| --- | --- | --- | --- | --- | --- | --- | --- | --- | --- | --- | --- | --- |
| 1 Organizational culture | 0.915 | **0.957** |  |  |  |  |  |  |  |  |  |  |
| 2 Network interaction | 0.841 | 0.564 | **0.917** |  |  |  |  |  |  |  |  |  |
| 3 Network size | 0.825 | 0.571 | 0.626 | **0.908** |  |  |  |  |  |  |  |  |
| 4 Community participation | 0.834 | 0.656 | 0.589 | 0.651 | **0.913** |  |  |  |  |  |  |  |
| 5 Community trust | 0.811 | 0.751 | 0.617 | 0.586 | 0.697 | **0.901** |  |  |  |  |  |  |
| 6 Reciprocity | 0.754 | 0.776 | 0.572 | 0.587 | 0.673 | 0.787 | **0.868** |  |  |  |  |  |
| 7 Cognitive social capital | 0.931 | 0.841 | 0.532 | 0.562 | 0.603 | 0.681 | 0.772 | **0.965** |  |  |  |  |
| 8 Prevention capabilities | 0.892 | 0.838 | 0.551 | 0.554 | 0.632 | 0.717 | 0.716 | 0.771 | **0.944** |  |  |  |
| 9 Preparedness capabilities | 0.836 | 0.8 | 0.53 | 0.512 | 0.653 | 0.702 | 0.693 | 0.74 | 0.907 | **0.914** |  |  |
| 10 Response capabilities | 0.867 | 0.755 | 0.489 | 0.467 | 0.621 | 0.675 | 0.679 | 0.687 | 0.853 | 0.918 | **0.931** |  |
| 11 Recovery capabilities | 0.93 | 0.778 | 0.512 | 0.493 | 0.623 | 0.727 | 0.716 | 0.73 | 0.858 | 0.899 | 0.894 | **0.964** |

Note: The diagonal represents the square root of average variance extracted (AVE)
