# Supplementary material for: Organizational culture, social capital, and emergency capacity in primary healthcare institutions: A cross-sectional structural equation modeling study comparing ordinary and older communities
Source: PLoS One. 2026 Jun 30;21(6):e0351875. doi: 10.1371/journal.pone.0351875 (PMC13318035; doi:10.1371/journal.pone.0351875)
Supplement: S4 Table — (DOCX) [file pone.0351875.s004.docx]

**S4a Table.**

| **Model** | **CMIN** | **DF** | ***P*** | **CMIN/DF** |
| --- | --- | --- | --- | --- |
| Unconstrained | 1057.357 | 166 | <0.001 | 6.37 |
| Measurement weights | 1067.074 | 176 | <0.001 | 6.063 |
| Structural weights | 1082.872 | 183 | <0.001 | 5.917 |
| Structural covariances | 1083.347 | 184 | <0.001 | 5.888 |
| Structural residuals | 1111.898 | 188 | <0.001 | 5.914 |
| Measurement residuals | 1268.794 | 203 | <0.001 | 6.25 |

**S4b Table*.***

| **Model** | **NFI** | **RFI** | **IFI** | **TLI** | **CFI** | **GFI** | **AGFI** | **RMSEA** | **AIC** | **ECVI** |
| --- | --- | --- | --- | --- | --- | --- | --- | --- | --- | --- |
| Unconstrained | 0.947 | 0.933 | 0.955 | 0.943 | 0.955 | 0.874 | 0.818 | 0.074 | 1205.357 | 1.229 |
| Measurement weights | 0.946 | 0.936 | 0.955 | 0.946 | 0.955 | 0.873 | 0.827 | 0.072 | 1195.074 | 1.218 |
| Structural weights | 0.946 | 0.937 | 0.954 | 0.947 | 0.954 | 0.872 | 0.832 | 0.071 | 1196.872 | 1.22 |
| Structural covariances | 0.945 | 0.938 | 0.954 | 0.948 | 0.954 | 0.872 | 0.833 | 0.071 | 1195.347 | 1.218 |
| Structural residuals | 0.944 | 0.937 | 0.953 | 0.948 | 0.953 | 0.869 | 0.833 | 0.071 | 1215.898 | 1.239 |
| Measurement residuals | 0.936 | 0.934 | 0.946 | 0.944 | 0.946 | 0.851 | 0.824 | 0.073 | 1342.794 | 1.369 |

Note: RMSEA = root mean square error of approximation; GFI = goodness-of-fit index; AGFI = adjusted goodness of fit index; NFI = normed fit index; CFI = comparative fit index; TLI = Tucker Lewis index; RFI = Robust Fitting Index; IFI = Incremental Fit Index; AIC: Akaike information criterion; ECVI = Expected cross-validation index.
