# Supplementary material for: Organizational culture, social capital, and emergency capacity in primary healthcare institutions: A cross-sectional structural equation modeling study comparing ordinary and older communities
Source: PLoS One. 2026 Jun 30;21(6):e0351875. doi: 10.1371/journal.pone.0351875 (PMC13318035; doi:10.1371/journal.pone.0351875)
Supplement: S5 Table — (DOCX) [file pone.0351875.s005.docx]

**S5 Table.**

| **Model** | **△DF** | **△CMIN** | ***P*** | **△NFI** | **△IFI** | **△RFI** | **△TLI** | **△CFI** |
| --- | --- | --- | --- | --- | --- | --- | --- | --- |
| Measurement weights | 10 | 9.717 | 0.466 | -0.001 | 0.000 | 0.003 | 0.003 | 0.000 |
| Structural weights | 7 | 15.798 | 0.027 | 0.000 | -0.001 | 0.001 | 0.001 | -0.001 |
| Structural covariances | 1 | 0.475 | 0.491 | -0.001 | 0.000 | 0.001 | 0.001 | 0.000 |
| Structural residuals | 4 | 28.551 | <0.001 | -0.001 | -0.001 | -0.001 | 0.000 | -0.001 |
| Measurement residuals | 15 | 156.896 | <0.001 | -0.008 | -0.007 | -0.003 | -0.004 | -0.007 |
