# Supplementary material for: Organizational culture, social capital, and emergency capacity in primary healthcare institutions: A cross-sectional structural equation modeling study comparing ordinary and older communities
Source: PLoS One. 2026 Jun 30;21(6):e0351875. doi: 10.1371/journal.pone.0351875 (PMC13318035; doi:10.1371/journal.pone.0351875)
Supplement: Attachment — Submitted filename: suggestions on revision.pdf [file pone.0351875.s011.pdf]

In response to the previous comments, the authors have introduced new English-language references in this revision, attempting to support the theoretical foundation of the core variable "Cultural Appeal" from an international perspective. This demonstrates the authors' willingness to improve the quality of the manuscript.

However, a detailed forensic review and content verification of the newly cited key literature—specifically Gevorgyan & Manucharova (2015) and Michaels & Rettig (2025)—reveals significant issues regarding Disciplinary Mismatch and Construct Validity. The current method of citation fails to strengthen the theoretical persuasiveness; instead, it exposes logical flaws in the theoretical construction, thereby weakening the academic rigor of the study.

1. Irrelevance of Gevorgyan & Manucharova (2015): This reference is a study within the fields of marketing and advertising. Its core independent variable, "Cultural Appeal," refers to rhetorical strategies (e.g., collectivism vs. individualism) used in advertising copy, and its dependent variables are consumer "brand recall" and "purchase attitudes." Equating a consumer's willingness to click on an advertisement in a low-stakes environment with a medical professional's emergency response behavior in a life-or-death situation presents a fundamental logical fallacy. Gevorgyan's study focuses on Persuasion, whereas your study focuses on Mobilization. Advertising appeal primarily influences transient psychological preferences, whereas organizational cultural appeal operates on enduring professional responsibility. Citing an advertising paper to support a healthcare management theory is far-fetched and conceptually inappropriate.
2. Misapplication of Michaels & Rettig (2025): This article belongs to the fields of environmental policy and political communication. The "culturally appealing climate frames" discussed in the text refer to how politicians utilize grand narratives (e.g., "Start-up Nation") to garner public support. You have cited this work to prove that cultural appeal acts as a positive driver for collective action. However, Michaels' research actually discusses the limitations and fragility of such drivers. Consequently, you have cited literature that may fundamentally contradict your own hypothesis.
3. Issues with the "Cultural Appeal" Construct: "Cultural Appeal" is not a standard construct in international management literature. In the context of Western management science, the activities described in your manuscript—such as establishing "culture walls" and practicing "Socialist Core Values"—would more accurately map to concepts such as Organizational Culture, Safety Climate, or Institutional Logic. Persisting in the use of the non-standard term "Cultural Appeal" has forced you to cite irrelevant literature from advertising and political science, resulting in the aforementioned theoretical inconsistencies.
4. Critique of Terror Management Theory (TMT): The core premise of TMT is that "Mortality Salience" triggers "Worldview Defense." This defense often manifests as cognitive rigidity, exclusion, and irrational behavior, rather than the rational and efficient collaboration required in emergencies. Furthermore, since this study did not measure "death anxiety" or "defense mechanisms," applying TMT appears to be an over-interpretation unsupported by your data. It would be more appropriate to frame major public health events as a "Mortality Salience Context," where the psychological defense mechanisms of medical staff—as the group closest to death—are critical to maintaining their operational capacity.
