# Supplementary material for: Organizational culture, social capital, and emergency capacity in primary healthcare institutions: A cross-sectional structural equation modeling study comparing ordinary and older communities
Source: PLoS One. 2026 Jun 30;21(6):e0351875. doi: 10.1371/journal.pone.0351875 (PMC13318035; doi:10.1371/journal.pone.0351875)
Supplement: Attachment — Submitted filename: Response to Reviewers(8.23).docx [file pone.0351875.s010.docx]

August 23, 2025

Chunyu Zhang

Academic Editor

PLOS ONE

**RE: “How do cultural appeal and social capital differ in influencing primary healthcare emergency capabilities in ordinary versus older communities?” Manuscript ID: PONE-D-24-36223**

Dear Editors:

Thank you for the opportunity to further revise our manuscript “**How do cultural appeal and social capital differ in influencing primary healthcare emergency capabilities in ordinary versus older communities**?”

We are grateful to the editor and the reviewers for the thoughtful suggestions and insights. The reviewers’ and editors’ comments were useful and helped to clarify and improve several aspects of the manuscript. The manuscript has been rechecked and the necessary changes have been made in accordance with the reviewers’ suggestions.

Please find below our a point-by-point response to the issues raised by the reviewers and editors. We are also submitting the revised version of the manuscript. For your convenience, all changes made are indicated using red font.

We hope that this version is suitable for publication in *PLOS ONE*. Thank you for your consideration, we look forward to hearing from you.

Xiaoting Zhang

School of Public Administration, Hangzhou Normal University

Hangzhou 311121, China

Email: zxtbing@hznu.edu.cn

**Point-by-point responses to the Reviewers’ comments**

**Reviewer #7**

**The research topic is relevant to scientists and public health organisations. Emergency response capacity in primary health care is essential for the resilience of health systems and for the safety of populations in critical contexts.**

**1. Methodological limitations and representativeness**

**The article is based on a sample of four cities in Zhejiang province, China, one of the most developed in China. This limits the generalisation of the results to less favourable regions or other countries, Therefore, these factors should be considered in the limitations of the study.**

**Response：**Thank you for raising this issue. In response to your concern about geographical sampling bias­­—specifically, the concentration of samples in relatively developed areas of Zhejiang Province—we have revised the “Research Limitations and Prospects” section to address this issue, as follows:

1. We explicitly acknowledge the geographical limitation of our sample—namely, its concentration in the developed eastern region of China—and note that this socioeconomic context represents a key methodological constraint.

2. We clearly state that, due to the absence of samples from less-developed regions, our findings may not be directly generalizable to underdeveloped areas such as central and western China.

3. We recommend that future research incorporate samples from regions across different levels of development in China and include cross-regional comparative analyses to explore the potential influence of socioeconomic differences on the study’s conclusions. We also suggest conducting cross-cultural comparative studies with samples from similar research contexts internationally to validate the robustness and generalizability of the findings.

**For details of the specific revisions, please see the revised manuscript [Research limitations and prospects] (pages 35-36, lines 715–736).**

**2. The impact of individual variables such as age and gender is not measured, focusing only on profession.**

**The literature indicates that these dimensions can affect perceptions about social capital and response capacity. It is suggested that these variables could be part of the suggestions for future research.**

**Response：**We thank the reviewer for their insightful comments and for highlighting an important methodological limitation in our study’s design. As noted, demographic factors—such as gender and age—may act as potential confounders in estimating the relationships between occupation, social capital, and healthcare emergency preparedness, which could affect the internal validity and inferential strength of our findings.

To address this methodological limitation more thoroughly, we have added a discussion in the **[Research Limitations and Prospects, lines 715–736]** section regarding the limitations of the demographic variables (e.g., occupation, age, gender). Specifically, we acknowledge that future research should consider gender, age, and similar variables either as primary explanatory variables or as moderating factors, and utilize interaction models or multi-group structural equation modeling to examine their potential impact on the relationship between social capital and healthcare emergency preparedness.

Although our preliminary analyses indicated that demographic characteristics such as occupation and community type significantly influenced emergency preparedness, gender and age did not reach statistical significance, this does not eliminate the possibility that these factors could influence outcomes under different societal or cultural contexts (Table1). Accordingly, we recommend that future studies validate these demographic influences across diverse regions and populations. Moreover, using more precise model specifications to estimate the independent effects of occupation and other demographic factors can help control for potential confounding, thereby enhancing both the internal and external validity—and generalizability—of the results.

Table 1. Univariate Analysis of Health Emergency Capacity(The table is only used to reply to the reviewer 's suggestion, not as the text or annex content.)

| **Variable** | **Categorization** | **Frequency (N)** | **Composition（%）** | **t/F** | ***P*** |
| --- | --- | --- | --- | --- | --- |
| Gender | Male | 216 | 22 | 2.241 | 0.349 |
|  | Female | 767 | 78 |  |  |
| Age | 18-30 years | 304 | 30.9 | 0.721 | 0.577 |
|  | 31-40 years | 369 | 37.5 |  |  |
|  | 18-30 years | 225 | 22.9 |  |  |
|  | 42-50 years | 73 | 7.4 |  |  |
|  | 51-60 years | 12 | 1.2 |  |  |
|  | 61 years and above | 304 | 30.9 |  |  |
| Educational level | Junior high school or below | 17 | 1.7 | 0.527 | 0.716 |
|  | High school | 47 | 4.8 |  |  |
|  | Associate degree | 251 | 25.5 |  |  |
|  | Bachelor’s degree or above | 668 | 68 |  |  |
| Marital status | Single | 181 | 18.4 | 0.050 | 0.985 |
|  | Married | 784 | 79.8 |  |  |
|  | Divorced and other | 18 | 1.8 |  |  |
| Position | Doctor | 404 | 41.1 | 3.111 | 0.009 |
|  | Nurse | 315 | 32 |  |  |
|  | Medical technician | 102 | 10.4 |  |  |
|  | Prevention | 88 | 9 |  |  |
|  | Administration | 30 | 3.1 |  |  |
|  | Other | 44 | 4.5 |  |  |
| Professional title | None | 125 | 12.7 | 0.165 | 0.920 |
|  | Junior | 484 | 49.2 |  |  |
|  | Intermediate | 286 | 29.1 |  |  |
|  | Senior | 88 | 9 |  |  |
| Years of service | Less than 1 year | 28 | 2.8 | 0.540 | 0.655 |
|  | 1-3 years | 117 | 11.9 |  |  |
|  | 4-6 years | 125 | 12.7 |  |  |
|  | More than 6 years | 713 | 72.5 |  |  |
| The type of community you work in | Ordinary community | 714 | 72.6 | 0.789 | 0.013 |
|  | old community | 269 | 27.4 |  |  |
| The presence of hazard identification and risk assessment management systems | Yes | 788 | 80.2 | 24.334 | <0.001 |
|  | No | 74 | 7.5 |  |  |
|  | Don't know | 121 | 12.3 |  |  |
| Accountability systems for public health emergencies | Yes | 865 | 88 | 30.239 | <0.001 |
|  | No | 47 | 4.8 |  |  |
|  | Don't know | 71 | 7.2 |  |  |
| The extent of the usefulness of smart tools | very helpful | 763 | 77.6 | 43787 | <0.001 |
|  | Helpful | 199 | 20.2 |  |  |
|  | Average | 19 | 1.9 |  |  |

**3. Measurements and validation of instruments**

**The authors should provide more explicit sources for the scales since they are not obtained from the entire international literature. The authors should explain the translation processes and the rationale behind their selection.**

**Response：**Thank you for your suggestion. It was correctly noted that the primary measurement instrument for cultural cohesion—or “cultural appeal”—used in our study is based on Chinese-language literature. We provide the following clarifications:

The measurement scale for cultural cohesion was principally adapted from the theoretical framework and definitions presented by Chen Li in the Chinese core journal Journalism Lover. Our research team then contextualized and refined these items to fit the realities of primary healthcare emergency responses in China xwahz.com.

It should be noted that there is presently no internationally established scale explicitly measuring “cultural cohesion of Chinese grassroots healthcare organizations during emergency conditions.” Thus, Chen Li’s work offers essential theoretical grounding and strong contextual relevance for constructing a culturally appropriate instrument.

Accordingly, we localized and streamlined the scale to align closely with our study context, ensuring the measurement items are highly relevant and accurate for our research setting. Since Chen Li’s study is available only in Chinese and is not indexed in major international databases, we will include the full Chinese PDF version along with an English translation of the key content in the revised submission for the reviewer’s convenience. The questionnaire was administered in Chinese to the local sample.

Reliability and validity metrics for our adapted instrument in our sample are strong: Cronbach’s α = 0.969, Composite Reliability (CR) = 0.970, and Average Variance Extracted (AVE) = 0.915, which attest to the measure’s excellent internal consistency and construct validity.

To ensure conceptual clarity and semantic equivalence in English, we followed a standard cross-cultural translation procedure: two bilingual experts with public health management backgrounds independently translated the Chinese questionnaire into English; discrepancies were resolved through discussion between the translators and the lead researcher to produce a unified English draft; a third bilingual expert then back-translated this into Chinese, and item-by-item comparisons with the original confirmed high conceptual equivalence between Chinese and English versions.

**The Chinese version and English translation of Chen Li’s study are selected**

国际传播能力建设的“五力”，“确定的是新时代国际传播能力建设的效能指标，隐含着全媒体传播、文化传播、形象传播、知识传播与舆论引导五个方面的内容，涵盖了传统意义上国际传播的各个领域，展现了对国际传播效能全方位提升的总体要求”[3]。“五力”建设是一项系统工程，“五力”互为表里，相互促进。经过近几年的努力，我们在国际传播能力建设上已经初具规模，但国际传播能力在内涵上还需进一步深化。“传播中华文化，讲好中国故事”是国际传播的核心内涵和重要内容。意大利共产党创始人之一的葛兰西曾提出“文化领导权”理论，认为优秀文化在多元文化竞争中会居于主导地位，自然就会获得“知识”和“道德领导权”。而在约瑟夫·奈那里，文化是一个国家最为重要的“软实力”，软实力靠的是自身的吸引力，而不是强迫别人去信服。[4]中华文化吸引力就是中华文化的吸引力、感染力、影响力、引领力，它既是国际传播能力建设的核心构成，是国际传播能力建设的灵魂和基础，也是最能表达和突出中国特色的一部分。因此，提升中华文化吸引力是我国国际传播能力建设的关键和重要内容，也是提高国际传播效能的重要内容。我们在文化自信的基础上，不断提升中华文化感召力，在文化的内涵挖掘、表达方式创新、传播方式提升等方面不断努力，将会显著推进我国国际传播能力的转型升级，增强中华文化的全球影响力，彰显中华文化的深刻内涵、独特魅力和优秀品质，进而取得良好的国际传播效果。

二、中华文化感召力的思想内涵与特征

（一）中华文化感召力的思想内涵

何谓文化？我国文化学家梁漱溟先生给文化下了一个无所不包的定义：文化，就是吾人生活所依靠之一切……盖人生诸于世界，无非做三件事：活着、动（工作）着、觉（思考并言说或者书写）着。人做这三件事又必须依靠三种方式：生活方式、行为方式、思维方式。人因三件事和一种方式而生活起来就是文化。[5]所以中华文化就是几千年来的文明进程中，中国人形成的生活方式、行为方式和思维方式。英国文化学者雷蒙德·威廉斯在《文化与社会》中提出，文化即“全部的生活方式，包括物质的、知识的和精神的”[6]。而马克思认为文化是“自然的人化”。文化是人类改造世界中一种巨大力量，可以称之为“文化力”或“文化生产力”。[7]因此可以说，文化感召力就是文化的一种内在表现。

中华文化感召力是文化软实力社会生活的本质，要具有吸引力、召唤力、感染力、凝聚力，能够教育人、鼓舞人、激励人、凝聚人、团结人。[8]文化感召力是文化软实力的一个重要体现，文化软实力的核心是文化所具有的感召力，正如孔子所认为的“近者悦，远者来”，中华文化感召力是中国人的生活方式、思维方式和行为方式所具有的感染力、号召力、引导力。

The "Five Powers" framework for international communication capacity building establishes performance indicators for the new era, encompassing five dimensions: omni-media communication, cultural dissemination, image cultivation, knowledge sharing, and public opinion guidance. It covers all aspects of traditional international communication and cultural exchanges, presenting comprehensive requirements for holistic improvement in communication effectiveness [2]. As a systematic project, the Five Powers complement each other and reinforce one another. While initial progress has been made in recent years, deeper development is needed in the substantive aspects of international communication capabilities. "Promoting China's outstanding culture and showcasing its evolving development" form the core essence of international communication. As Gramsci, co-founder of the Italian Communist Party, proposed the theory of "cultural hegemony," where superior cultures naturally gain "knowledge and moral leadership" in multicultural competition [3]. Joseph Nye further emphasized that culture constitutes a nation's most vital "soft power"—a strength derived from intrinsic appeal rather than coercion [4]. The cultural magnetism of Chinese heritage—its attractiveness, influence, and guiding role—serves as the cornerstone of international communication capacity building. This element embodies Chinese characteristics most vividly. Therefore, enhancing this cultural magnetism is both the key focus and essential component of international communication capacity development, significantly improving global communication effectiveness. Building on cultural confidence, we are continuously enhancing the appeal of Chinese culture through in-depth exploration of its essence, innovative approaches to expression, and improved dissemination effectiveness. These efforts will significantly advance the transformation and upgrading of China's international communication capabilities, amplify the global influence of Chinese culture, and highlight its profound depth, unique charm, and outstanding qualities—ultimately achieving remarkable international communication outcomes.

**The Ideological Content and Characteristics of the Appeal to Chinese Culture**

**(1) The Ideological Connotation of the Appeal of Chinese Culture**

What is culture? Chinese cultural scholar Liang Shuming provided an all-encompassing definition: Culture is everything we rely on for living... As humans exist in the world, we essentially engage in three fundamental activities: living, working, and thinking (expressing thoughts through speech or writing). To perform these activities, we must adopt three modes of existence: lifestyle, behavior, and thought. The combination of these three aspects and their corresponding modes constitutes culture. [5] Thus, Chinese culture represents the lifestyle, behavioral patterns, and cognitive frameworks developed by the Chinese people over millennia of civilizational development. British cultural scholar Raymond W. Kwan proposed in "Culture and Society" that culture encompasses "the totality of life—material, intellectual, and spiritual" [6]. Marx viewed culture as "the humanization of nature." Culture serves as humanity's immense power to transform the world, often termed "cultural force" or "cultural productivity." [7] Therefore, cultural appeal can be seen as an inherent manifestation of this cultural force.

The cultural appeal of Chinese civilization embodies the essence of culture reflecting real social life. It should possess attractiveness, appeal, influence, and cohesion, capable of educating, inspiring, motivating, urging, and uniting people. [8] Cultural appeal serves as a crucial manifestation of cultural soft power, whose core lies in the inherent appeal of culture. As Confucius observed, "Those nearby are pleased, while those afar come." The cultural appeal of Chinese civilization refers to the infectiousness, rallying power, and guiding influence inherent in the lifestyle, thinking patterns, and behavioral norms of the Chinese people.

**4. Discussion of innovation and marginal contributions**

**The authors should refer to recent international literature on social capital, cultural appeal and emergency management, to specify the advances of this study in relation to existing approaches.**

**Response：**We reviewed recent international literature and, based on both the literature review and a comparative analysis, supplemented the Conclusion and Theoretical Implications sections to emphasize the threefold innovation of this study’s research perspective and methodology:

(1) Regarding the research focus, we selected primary healthcare institutions as the core analytic unit, unlike most international studies that concentrate on large hospitals or macro-level emergency systems. Primary healthcare institutions act as the “nerve endings” of the health emergency system and play a direct role in community-level crisis response. However, the international literature places limited emphasis on this domain. Our study elucidates the mechanisms shaping emergency preparedness in grassroots settings, thereby offering empirical, context-specific contributions to the global discourse on health emergency management.

(2) In terms of theoretical variable integration, this study systematically incorporates cultural appeal as a key antecedent variable within the theoretical framework of emergency preparedness. This extends beyond existing models, which largely hinge on structural or cognitive social capital and technological or resource-based factors. Our empirical analysis confirms a positive effect of cultural appeal on grassroots emergency preparedness, thereby offering a theoretical anchor for understanding the critical influence of informal institutions and cultural soft power in crisis response.

(3) On methodology and contextualized comparison, we innovatively employ multi-group structural equation modeling based on community types—specifically, old communities versus ordinary community—to systematically test for contextual differences in causal paths. This method reveals both universal and unique mechanisms driving emergency capability and provides concrete process evidence aligning with Shi et al., who propose that “resilience of healthcare organizations is influenced more broadly by community and healthcare systems.” Our findings clarify how community type shapes the influence and relative importance of key driving factors.

The source referenced is [Shi W, Chen R, Wang K, Wang Y, Gui L. Exploring hospital resilience protective or risk factors: lessons for future disaster response efforts. Front Public Health. 2024 Mar 27;12:1378257. doi: 10.3389/fpubh.2024.1378257.]

For details of the specific revisions, please see the revised manuscript **[Conclusion] (pages 32-33, lines 642–662)**, **[Theoretical contribution] (pages 33-34, lines 670–680)** .

**5. Argumentative redundancy and structuring**

**I suggest simplifying the text in the discussion of results and conclusions. The authors should rewrite these sections by prioritising a structure that includes contributions to theory, management of organisations, limitations, and suggestions for future research. Currently, the text exhibits a degree of repetition, leading the reader to become disoriented among numerous sections.**

**Response：**We sincerely appreciate your valuable suggestions. Based on your feedback, we have revised the Discussion and Conclusion sections, as follows:

In the conclusion section, we have reorganized the content into four distinct subsections: Conclusion, Theoretical Contributions, Managerial Implications, and Limitations and Directions for Future Research. We have removed several instances of repetitive discussion and, within the Managerial Implications subsection, proposed actionable recommendations for enhancing cultural appeal and optimizing social capital allocation in primary healthcare institutions, based on the study’s key findings, thereby improving clarity and coherence.

In the discussion section, we have streamlined and refined the content, restructuring the original subsections 5.1–5.5 into three more thematically focused discussion blocks: a. The direct effects and interpretations of the main variables (cultural appeal and three types of social capital) on health emergency capabilities; b. The mediating mechanisms of social capital—particularly across different types—in the relationship between cultural appeal and health emergency preparedness, along with comparative analyses; c. The multi-group effects of community type on the above relationships **[Discussion] (pages 29-31, lines 558–639) , [Summarize] (pages 32-36, lines 642–736)**.

**6. The authors should ensure that cited references are accessible internationally. Some references are difficult to locate, especially Chinese articles that do not have a translation or complete information. It is recommended to include more up-to-date and relevant international references.**

**Response：**We thank the reviewer for their valuable comments. We fully understand and appreciate the importance of ensuring the international accessibility of cited literature. In response to the concerns regarding references, we have made the following revisions:

We conducted a comprehensive review and updated the reference list, adding two recent international publications that are closely related to the topic of this study, thereby ensuring that the cited literature is accessible within the international academic community.

The only Chinese-language reference retained from the original manuscript represents a key primary source of original findings specific to the study region, for which no equivalent international alternative currently exists. To enhance its international accessibility and verifiability, in the reference list, we have provided a complete English translation of the title, the journal’s standardized English name, volume, issue, page numbers, and DOI, strictly following the citation format requirements of PLOS ONE. In addition, to facilitate review by the editors and reviewers, we will include the full PDF of this Chinese reference as supplementary material upon submission of the revised manuscript.

**Reviewer #8**

**The authors have provided a detailed and appropriate response to the previous round of review comments and revised the manuscript accordingly. However, the following issues remain:**

**1.The reference list requires a thorough and careful revision to fully comply with the journal's formatting and citation style guidelines. There are systematic inconsistencies in formatting, punctuation, and author names. Some Chinese references are not retrievable; please provide the correct citation information. Such lack of attention to detail diminishes the academic quality of the paper.**

**Response：**Thank you for this valuable feedback. We fully understand your concern regarding the potentially ambiguous meaning of “cultural appeal,” which stems from the direct translation and interpretation of the Chinese concept Wénhuà Gǎnzhàolì.

Our literature review indicates that this concept is rooted in the Chinese context of management and public policy, emphasizing the capacity of mainstream values and collective spirit to inspire, unite, and mobilize individuals. Many scholars use the term “appeal or inspirational power” in combination with “culture” to describe the cultural force that generates recognition and identification among others. In the public health emergency practices in China, the cultural appeal—stemming from Chinese traditional culture, core values and organizational culture—significantly enhances healthcare workers’ sense of mission and initiative in learning emergency response knowledge, thereby strengthening overall emergency capacity. Therefore, in this study, we adopt the construct and, as defined in the Research Hypotheses section, conceptualize it as the intrinsic capacity—embedded in shared values, cultural norms, and collective goals within organizations and social networks during a public health crisis—to evoke members’ sense of responsibility and organizational identification.

To strengthen the conceptual foundation, we have supplemented the revised manuscript with relevant theoretical underpinnings. Drawing on Edgar Schein’s organizational culture theory, which distinguishes between artifacts, espoused values and beliefs, and basic assumptions, we note that an organization’s shared beliefs and values—manifested in its strategic objectives and ideology—become more cohesive once internalized as “basic assumptions.” This process aligns closely with the mechanism of cultural appeal as defined in our study. Accordingly, we have expanded the Research Hypotheses section to elaborate on the conceptual connection between cultural appeal and organizational culture theory. **For details of the specific revisions, please see the revised manuscript [****Literature review and hypotheses] (pages 6-8, lines 137–146, lines164-169).**

Regarding the reviewer’s concern regarding the measurement source, we have provided a detailed explanation in our response to Reviewer 1 (comment 3). Briefly, the cultural appeal scale was self-developed, primarily informed by the theoretical framework and definitions proposed by Chen Li (2023) in the Chinese core journal Journalism Lover, and tailored by our research team to reflect the specific context of emergency health responses in Chinese grassroots medical institutions.

The sources referenced are [Schein, E. H. (1985). Organizational Culture and Leadership. San Francisco, CA: Jossey‑Bass Publishers; Abdelrahman, M., Papamichail, K., Hammady, R. et al. Organisational Culture and Knowledge Management Systems Adoption: Examining Employee Usage in Multinational Corporations. Glob J Flex Syst Manag (2025). doi.org/10.1007/s40171-025-00448-w].
