# Supplementary material for: Organizational culture, social capital, and emergency capacity in primary healthcare institutions: A cross-sectional structural equation modeling study comparing ordinary and older communities
Source: PLoS One. 2026 Jun 30;21(6):e0351875. doi: 10.1371/journal.pone.0351875 (PMC13318035; doi:10.1371/journal.pone.0351875)
Supplement: Attachment — Submitted filename: Response to Reviewers.docx [file pone.0351875.s012.docx]

November 1, 2025

Chunyu Zhang

Academic Editor

PLOS ONE

**RE: “How do cultural appeal and social capital differ in influencing primary healthcare emergency capabilities in ordinary versus older communities?” Manuscript ID: PONE-D-24-36223**

Dear Editor:

Thank you for the opportunity to further revise our manuscript. We are grateful to you and the reviewers for the thoughtful suggestions and insights. The comments were useful and helped to clarify and improve several aspects of the manuscript. The manuscript has been rechecked and the necessary changes have been made in accordance with the reviewers’ suggestions.

Please find below a point-by-point response to the issues raised. We are also submitting the revised version of the manuscript. For your convenience, the revised sections are indicated using red font.

We hope that this version is suitable for publication in *PLOS ONE*. Thank you for your consideration, we look forward to hearing from you.

Xiaoting Zhang

School of Public Administration, Hangzhou Normal University

Hangzhou 311121, China

Email: zxtbing@hznu.edu.cn

**Point-by-point responses to the Reviewers’ comments**

**Academic Editor**

While the manuscript addresses an important topic concerning social capital and emergency capacities in primary healthcare institutions, it still falls short of PLOS ONE’s publication standards in several aspects. In particular, (1) the presence of Chinese-language content in both the response letter and supplementary sections is unacceptable for an international English journal, and (2) the inclusion of Chinese-language sources translated into English does not meet the requirement for internationally retrievable references. In addition, the theoretical construct of “cultural appeal” remains conceptually vague and lacks a clear grounding in established international literature. The manuscript would benefit from reframing this construct within recognized theories of organizational culture or cultural competence, replacing all non-English citations with recent international studies, and ensuring full linguistic and stylistic consistency across the text. A thorough language revision by a professional English editor is strongly recommended before resubmission.

**Response（1）:** We sincerely thank the reviewer for their insightful comments. We fully understand PLOS ONE’s requirements regarding the use of English for international readership. We have carefully reviewed the entire manuscript and have removed all Chinese-language content from both the response letter and supplementary sections.

**Response（2）:** We appreciate the reviewer’s suggestion to replace non-English references with internationally retrievable sources. In compliance with this, we have replaced all Chinese-language references with internationally recognized English-language literature. We consulted the latest international studies in the relevant fields and updated the reference list to ensure traceability and international relevance. We have also engaged a professional English editor to perform a thorough language revision, ensuring stylistic consistency and academic quality.

Regarding the theoretical construct of “cultural appeal,” we recognize that this concept may be less familiar in international literature. In this study, we framed “cultural appeal” within the well-established theories of organizational culture and cultural capital. Specifically, cultural appeal refers to the capacity of cultural values and beliefs to elicit emotional resonance and collective identification within a group, which in turn shapes individual behavioral choices in contexts such as emergency management and crisis response.

To further clarify this construct, we draw on Schein’s theory of organizational culture, which emphasizes that shared values and beliefs, embedded in an organization’s strategic goals and ideology, profoundly influence members’ attitudes, motivations, and behaviors. Additionally, we integrate Bourdieu’s theory of cultural capital, which underscores the role of intangible cultural resources (such as values, beliefs, and traditions) in mobilizing collective action. In the context of emergency management, we argue that cultural appeal functions as a form of cultural capital that fosters collective identification and cooperation among members, thus enhancing the emergency response capacity of primary healthcare institutions.

For international readers, this theoretical framing connects the concept of cultural appeal with both organizational culture and cultural capital theories, which have been widely applied in studies of emergency response, crisis management, and organizational behavior. We have revised the manuscript to reflect these theoretical foundations and incorporated relevant international research to enhance its scholarly value.

Cultural appeal is a concept drawn from the Chinese managerial context, referring to the capacity of cultural frames to elicit affective resonance and collective identification within a group, thereby shaping individual behavioral choices. This concept is grounded in Schein’s theory of organizational culture, which posits that shared values and beliefs embedded in an organization’s culture influence members’ attitudes and behaviors. In this study, cultural appeal is framed as a form of intangible cultural capital, drawing from Bourdieu’s theory of cultural capital. Bourdieu emphasizes that cultural capital—particularly intangible forms like values, beliefs, and traditions—plays a crucial role in mobilizing collective action. In the context of emergency management, cultural appeal can enhance cooperation and rapid response, fostering a strong sense of collective identity and shared purpose that improves institutional emergency capacity.

The sources referenced are

Gevorgyan G, & Manucharova, N. The effects of cultural appeal, product involvement, and ethnic identity on attitudes and recall in online advertising. Chinese Journal of Communication, 2015,8(2):196-220.

Michaels L, & Rettig E. A renewable light unto the nations? Modelling the limits of culturally appealing climate frames: a case study from Israel. Journal of Environmental Policy & Planning,2025,27(4):376-389.

Schein, E. H. (2010). Organizational Culture and Leadership. Jossey-Bass.

Bourdieu, P. (1986). The Forms of Capital. In J. G. Richardson (Ed.), Handbook of Theory and Research for the Sociology of Education. Greenwood Press.

**[Literature review and hypotheses] (pages 6-7, lines 138–153):** “The concept of *cultural appeal* originates in the Chinese managerial context and refers to the transmission of cultural forces that elicit affective resonance and collective identification within a group, thereby shaping individual behavioral choices. Schein’s organizational culture theory provides theoretical support for this construct: shared beliefs and cultural values are reflected in an organization’s strategic goals and ideology, become embedded in members’ cognitions, and consequently influence their attitudes, motivation, and collaborative behavior [24]. In this sense, cultural appeal strengthens members’ cultural identification and facilitates behavioral mobilization and collective responses. The core idea of cultural appeal also aligns with Bourdieu’s theory of cultural capital. Bourdieu emphasizes that the distribution of cultural capital—especially intangible forms such as traditions, values, and beliefs—can drive collective mobilization and social change [25]. Within this theoretical framework, cultural appeal can be viewed as a form of intangible cultural capital that, by fostering collective identification, promotes active participation and cooperation among organizational members, thereby enhancing overall emergency response capacity.”

**Reviewer #10**

**This paper explores the impacts of cultural appeal and social capital on health care’s emergency capacities.**

（1）I don’t understand why the authors introduce the term ‘cultural appeal,’ and I cannot find any relevant theories or articles on cultural appeal. I am not sure how such research focusing on cultural appeal would benefit the international audience of PLOS One.

**Response:** Thank you for your thoughtful review and valuable feedback. We appreciate the opportunity to clarify the introduction of the term “cultural appeal” and its relevance to our study.

First, *cultural appeal* is a concept we propose to describe the influence of cultural values and organizational goals in evoking emotional resonance and motivating behavioral change. Drawing from organizational culture theory, we explore how cultural norms and shared values within organizations, particularly in emergency management, can strengthen cooperation and enhance response capacity. While the concept may not be widely established in emergency management, we believe it offers a novel perspective for improving the operational efficiency of primary care institutions, especially during crises. By reinforcing cultural appeal, organizations can foster a sense of responsibility and engagement among their members, leading to more effective emergency responses.

Regarding the absence of relevant theories or studies, we have cited related research that draws from established international theories such as organizational culture and cultural capital. While “cultural appeal” may be more common in Chinese discourse, its core principles align with internationally recognized concepts. For example, research by Gennadi and Naira on cultural identification shows how collective values influence individual behaviors, especially in culturally congruent contexts. Similarly, Michaels and Rettig’s work on culturally appealing climate frames demonstrates how cultural values can dominate public discourse and drive action.

Additionally, Bourdieu’s cultural capital theory provides further support, particularly regarding intangible cultural assets like values and traditions that help mobilize collective action. In our study, we view cultural appeal as a form of cultural capital that enhances emergency response by fostering collective identification and shared organizational values. This framing aligns with Bourdieu’s ideas, where cultural capital can generate emotional energy and social mobilization within organizations.

As for the relevance of this work to the international readership of PLOS ONE, we highlight that the influence of culture on emergency management is gaining global attention, especially in the wake of public health emergencies. By focusing on cultural appeal within Chinese primary healthcare institutions, our study provides insights that are applicable to multicultural contexts worldwide. Understanding how cultural forces impact emergency management can offer valuable lessons for improving public health response capacities across different national and regional settings.

In the revised manuscript, we have further elaborated on the theoretical foundations of cultural appeal and integrated additional international literature to better contextualize the concept and strengthen its relevance for global readers. We believe this conceptual framework can enrich the understanding of emergency management in diverse cultural environments. We greatly appreciate your insightful comments and look forward to your feedback. **[Literature review and hypotheses] (pages 6-7, lines 138–153)**

The sources referenced are

Gevorgyan G, & Manucharova, N. The effects of cultural appeal, product involvement, and ethnic identity on attitudes and recall in online advertising. Chinese Journal of Communication, 2015,8(2):196-220.

Michaels L, & Rettig E. A renewable light unto the nations? Modelling the limits of culturally appealing climate frames: a case study from Israel. Journal of Environmental Policy & Planning, 2025,27(4):376-389.

（2）I highly suggest that authors present their survey questions in the main text so that the readers can understand how they constructed variables.

**Response:** Thank you for your valuable suggestion. We agree that presenting the survey questions directly in the main text would help readers better understand the construction of variables. However, due to space limitations, we have opted to include a brief description of the instrument design and variable operationalization in the **Methods** section (**pages 15-17, lines 344-345, 358-359, 361-362,373-376**). These descriptions include the number of items, variable dimensions, and representative items, which help clarify the construction of each variable. The full wording of all survey items is provided in the Appendix for those who wish to examine the complete instrument. We believe this approach balances clarity and space constraints while maintaining transparency in our research methodology. We appreciate your thoughtful suggestion, and we trust that these revisions enhance the manuscript’s comprehensiveness and readability.

（3）The survey data mainly came from healthcare providers; therefore, lack of an objective measure of health institutions’ real emergency capacity. Based on Table S2, it seems the dependent variable, health emergency capabilities (e.g., prevention, preparedness, response, and recovery), is health providers’ perceptions of their organizations’ emergency capacities. In considering the wider social impacts of a health emergency, the authors should use more objective measures of emergency capacity.

**Response:** We understand your point and agree that objective indicators are ideal. In the revision, we explicitly acknowledge this limitation and discuss how future research can incorporate a broader set of objective measures of emergency capacity—particularly response data from actual events and hard preparedness indicators (e.g., stockpiles of emergency supplies, training frequency). Comparative analyses using such data would enable a more comprehensive evaluation.

Regarding our choice to measure primary healthcare institutions’ emergency capacity via healthcare providers’ perceptions of their organizations—rather than purely objective indicators—we believe this approach also has merit. Providers’ assessments can effectively reflect day-to-day readiness and response capacity in routine operations. While objective indicators such as response times, and materials reserves offer direct measures, gathering such data can be hindered by availability, collection burden, and timing. Moreover, objective data that can be compared across regions and institutions may be lacking. Using providers’ subjective evaluations not only captures institutions’ practical emergency capacity but also illuminates cultural and social factors that influence emergency management—factors that are difficult to gauge using traditional objective metrics.

For measurement, we adopted the four widely used dimensions of prevention, preparedness, response, and recovery. This framework has been validated in multiple international studies as an effective standard for assessing health emergency capacity. Specifically, these four dimensions reflect the comprehensive capabilities of health institutions when facing public health emergencies, covering the full cycle from pre-event planning to post-event recovery and thereby ensuring a holistic assessment (Smith).

The reference sources are

Gevorgyan G, & Manucharova N .The effects of cultural appeal, product involvement, and ethnic identity on attitudes and recall in online advertising. Chinese Journal of Communication, 2015,8(2):196-220.

Michaels L ,Rettig E .A renewable light unto the nations? Modelling the limits of culturally appealing climate frames: a case study from Israel. Journal of Environmental Policy & Planning, 2025, 27(4):376-389.

**[Research limitations and prospects] (page 37, lines 758–766):** “Furthermore, this study assesses health emergency capacity by surveying healthcare providers’ perceptions of their organizations’ capabilities, which relies on subjective perceptions rather than objective measurement. Although such perceptions can reflect providers’ understanding of institutional readiness, they may also be influenced by factors such as cultural appeal and social capital. Accordingly, future research could incorporate a broad set of objective indicators—for example, actual performance in emergency drills, allocation of emergency resources, and outcomes of responses to public health incidents—to provide a more comprehensive assessment of health emergency capacity.”

（4）The authors found that cultural appeal has positive impacts on emergency management. Based on the S2 Table, I wonder how specific measurement items of cultural appeal, including “having a corresponding culture wall to reflect the organization’s vision and cultural characteristics”, and “implementing socialist core values,” would impact emergency capacities. The authors should explicitly discuss and explain the intrinsic relationship between cultural appeals (e.g., cultural walls, socialist core values) on emergency capacities and their implications to the international audience.

**Response:** Thank you for your valuable comments. In response to your question about the relationship between cultural appeal and emergency management, we have revised the Discussion and Managerial implications sections.

1) Intrinsic relationship between cultural appeal and emergency response capacity.

In line with Schein’s model, we now conceptualize a “culture wall” as a visible cultural artifact that renders shared goals and norms salient at the point of work. Such artifacts function as cues that signal priorities to staff (e.g., “safety/response first”). This cueing can strengthen the unit’s safety climate—shared perceptions that safety and preparedness are prioritized—thereby increasing coordination, protocol adherence, and timely action during drills and incidents. Likewise, “implementing the Socialist Core Values” is treated analytically as espoused values or ethical climate (i.e., the formal articulation of prosocial and duty-of-care norms). When staff perceive value congruence with these espoused norms, research shows stronger organizational commitment and cooperative behavior, which, together with social identity and collective efficacy, support rapid, role-consistent responses under pressure. We describe these pathways in the revised text and add citations to the organizational culture/climate, commitment/fit, social identity, and collective efficacy literatures.

2) Implications for international readers.
We additionally elaborate on the potential applicability of cultural appeal in global—particularly multicultural—settings. We emphasize the importance of cultural identity and cultural climate in fostering social solidarity and enhancing emergency responsiveness, with the expectation that this framework can inform strategies to improve the efficiency of primary-level emergency management during public health emergencies. We are grateful for your thoughtful comments; these revisions have strengthened both the theoretical depth and the practical significance of our manuscript.

**[Discussion] (page 29, lines 568–587):** “The findings of this study suggest that primary healthcare institutions’ cultural appeal may exert a positive influence on their health emergency capacity, particularly as captured by specific measurement items such as the culture wall and the implementation of the Socialist Core Values. This result is consistent with Wolf-Fordham [53]. Specifically, the findings suggest that higher levels of cultural appeal strengthen ideological guidance, shape collective agency, and enhance the emergency response capabilities of healthcare institutions. As a concrete embodiment of organizational culture, the culture wall deepens staff identification with shared values, thereby increasing the collective capacity for coordinated action and facilitating efficient emergency response. Likewise, implementing the Socialist Core Values signals alignment with mainstream societal values, heightening staff members’ sense of social responsibility and collectivism, and further improving team coordination and decision-making efficiency under time pressure. Terror Management Theory suggest that individuals respond positively to elements that uphold their cultural values, and such values function as a buffer to anxiety, enabling proactive responses during crises [54]. In this study, both the culture wall and the Socialist Core Values help healthcare workers remain composed and respond actively in the face of crises, thereby strengthening health emergency capacity. Endorsing appropriate values also encourages healthcare workers to assume responsibility and confront challenges, ensuring high-quality emergency performance and, ultimately, improved institutional capacity [55].”

**[Discussion] (page 32, lines 655–662):** “From an international perspective, this finding has important implications. Despite differences across communities in the types of social capital and the distribution of resources, collective action galvanized by cultural appeal can transcend physical community boundaries, consolidate social forces, and enhance emergency responsiveness during public health emergencies. These insights offer valuable guidance for health emergency management worldwide. In particular, when confronting crises across diverse social environments and cultural contexts, cultural factors continue to play a critical role.”

**[Managerial implications] (page 35, lines 705–716):** “Cultural appeal as reflected in concrete practices such as the culture wall and the implementation of the Socialist Core Values, has a significant positive impact on the emergency capacity of primary healthcare institutions strengthening emergency capacity and catalyzing collective action. Accordingly, primary healthcare institutions should regularly organize cultural training and curate/maintain a culture wall to help staff understand and embody the organization’s core values. By collaborating with community organizations and other stakeholders to co-develop shared spatial layouts and infrastructure—and by conducting cultural engagement activities alongside emergency drills—institutions can further reinforce cultural identification and enhance the overall cultural climate, strengthen community network ties and normative consensus, and provide an actionable, closed-loop approach to enhancing health emergency capacity.”
