# Supplementary material for: Organizational culture, social capital, and emergency capacity in primary healthcare institutions: A cross-sectional structural equation modeling study comparing ordinary and older communities
Source: PLoS One. 2026 Jun 30;21(6):e0351875. doi: 10.1371/journal.pone.0351875 (PMC13318035; doi:10.1371/journal.pone.0351875)
Supplement: Attachment — Submitted filename: Response to Reviewers.doc [file pone.0351875.s013.doc]

**February 26, 2026**

Chunyu Zhang

Academic Editor

PLOS ONE

**RE: “How do cultural appeal and social capital differ in influencing primary healthcare emergency capabilities in ordinary versus older communities?” Manuscript ID: PONE-D-24-36223**

Dear Editors:

Thank you for the opportunity to further revise our manuscript “**How do cultural appeal and social capital differ in influencing primary healthcare emergency capabilities in ordinary versus older communities**?”

We are grateful to the editor and the reviewers for the thoughtful suggestions and insights. The reviewers’ and editors’ comments were useful and helped to clarify and improve several aspects of the manuscript. The manuscript has been rechecked and the necessary changes have been made in accordance with the reviewers’ suggestions.

Please find below our a point-by-point response to the issues raised by the reviewers and editors. We are also submitting the revised version of the manuscript. For your convenience, all changes made are indicated using red font.

We hope that this version is suitable for publication in *PLOS ONE*. Thank you for your consideration, we look forward to hearing from you.

Xiaoting Zhang

School of Public Administration, Hangzhou Normal University

Hangzhou 311121, China

Email: zxtbing@hznu.edu.cn

**Point-by-point responses to the Reviewers’ comments**

**Academic Editor**

After carefully considering the revised manuscript and the reviewers’ latest comments, I find that the paper has improved in presentation and responsiveness. However, substantial concerns remain regarding the conceptualization and theoretical grounding of the core construct, “Cultural Appeal.”

Multiple reviewers independently note that the current theoretical justification relies on literature drawn from marketing, advertising, and political communication, which raises issues of disciplinary mismatch and construct validity. In particular, the way “cultural appeal” is operationalized and theorized does not yet align convincingly with established frameworks in organizational studies, healthcare management, or emergency governance. As a result, the added citations do not sufficiently strengthen the theoretical argument and, in some cases, introduce internal inconsistencies.

Relatedly, the use of Terror Management Theory in the discussion is viewed as insufficiently supported by the study design, as key psychological mechanisms central to the theory were not directly measured. This creates a risk of theoretical overextension.

In addition, as noted by Reviewer 11, the term “culture” may be misleading for an international readership, as the study focuses on organizational or work-related culture rather than ethnic or national culture. The manuscript would benefit from clearer early-stage clarification and a more explicit articulation of why this study matters, including clearer policy and practical implications and identification of intended beneficiaries.

Taken together, these concerns indicate that the manuscript requires substantial theoretical revision, particularly with respect to construct definition, theoretical anchoring, and disciplinary coherence. Further revision would need to go beyond adding or substituting references and instead involve a careful reconsideration of the core conceptual framework.

**Response(1):** Thank you very much for your valuable suggestions. In the original manuscript, the construct labeled “Cultural Appeal” was drawn from a China-specific context and is not a standard term in international management or organizational research. Moreover, because its conceptual boundaries were insufficiently specified, our theoretical argument relied on interdisciplinary literature grounded in divergent construct systems, which resulted in dispersed theoretical anchoring and internal inconsistencies. Following the editor’s and reviewers’ recommendations, we therefore corrected this variable and renamed the core construct throughout the manuscript as “Organizational Culture.” This revision is not a mere terminological substitution but a conceptual re-grounding of the focal construct. Specifically, we have consistently replaced the original label with “Organizational Culture,” and, on this basis, re-specified its definitional scope, theoretical positioning, and measurement interpretation to align with established frameworks in organizational studies, healthcare management, and emergency governance. This re-grounding addresses the "construct validity" issues by ensuring that our independent variable is mapped to internationally recognized concept systems. Specifically, we now theorize that the organizational culture in primary healthcare institutions—manifested through artifacts like "culture walls" and espoused "core values"—is internalized by staff, thereby directly influencing their emergency response capabilities.

**Response(2):**The reviewers noted that the added citations in the previous revision introduced internal inconsistencies. We acknowledge that attempting to "bridge" marketing literature with healthcare management created a dispersed theoretical focus. In the current revision, we have removed all references to advertising and political communication. Instead, we have integrated literature that explores the role of organizational culture in "health system resilience"—defined as the system's ability to prepare, manage, and learn from shocks. We now argue that organizational culture serves as the "social glue" that impacts communication patterns, decision-making, and employee engagement during public health crises.

We rebuilt the theoretical rationale and hypothesis development within organizational studies/healthcare management and used Schein’s organizational culture perspective as a guiding theoretical lens (not as a claim of fully operationalizing all cultural layers). This lens supports the mechanism that shared values and mission consensus can facilitate coordination and collective action in high-stakes response settings. We adopted Edgar Schein’s organizational culture theory as the central theoretical anchor and rebuilt the logic of hypothesis development and discussion accordingly. The specific changes can be found in the revised manuscript: Introduction (page 4, lines 77–88), Literature review and hypotheses (pages 6-7, lines 145–156; page 11, lines 240–246)).

**Response(3):** We agree with the reviewers that because we did not directly measure death anxiety or specific psychological defense mechanisms, using TMT constituted a "theoretical overextension". Attempting to infer "mortality salience" without direct measurement is speculative and outside the scope of our cross-sectional SEM design. We also specify the deletion locations and the substituted explanations in our point-by-point response to Reviewer 11. The specific changes can be found in the revised manuscript: Discussion (page 29, lines 565–580).

**[****Introduction] (page 4, lines 77–88) :** “Organizational culture” refers to the collective consciousness, shared values, ethical principles, and normative expectations that organizational members develop and uphold through organizational practices under specific socio-economic conditions [14,15]. A constructive organizational culture can subtly motivate members to engage proactively in emergency-related tasks and can strengthen organizational cohesion and response capability. Building on this understanding, organizational culture theory emphasizes that cultural attributes within organizations shape members’ behaviors and decision-making processes[16]. Although organizational culture is largely tacit and intangible, it constitutes one of the most enduring and foundational characteristics of an organization. To date, however, empirical research that treats organizational culture as a key explanatory variable for enhancing public health emergency capacity in primary healthcare institutions remains limited.

**[Literature review and hypotheses] (****pages 6-7, lines 145–156):** The functions of organizational culture include fostering internal integration and coordination by sustaining organizational stability and cultivating a sense of belonging and altruism, all of which guide and shape employees’ behaviors . Schein’s theory of organizational culture further emphasizes that shared beliefs and cultural values are embedded in strategic goals and organizational ideology, becoming internalized in members’ cognitions and thereby influencing their attitudes, motivation, and collaborative behaviors [24].Previous research has demonstrated that shared cultural identity and a positive organizational climate can strengthen organizational cohesion and willingness to cooperate, thereby enhancing risk response and crisis management capabilities [25,26]. An analysis of tertiary hospital organizational culture by Reem et al. revealed a positive correlation between cultural norms, organizational goals, and emergency response capacity [27].

**[Literature review and hypotheses] (****page 11, lines 240–246):** Organizational culture fosters internal cohesion and a shared public mission, which in turn catalyzes and mobilizes the social capital embedded within the community ecosystems of primary healthcare institutions (PHIs). Extant literature (e.g., [42]) suggests that the synergy between cultural orientation and institutional support fortifies both structural and relational social capital in marginalized regions. This reinforcement significantly enhances the "adaptive resilience" of PHIs, enabling more robust recovery and response mechanisms in the aftermath of natural disasters.

**Reviewer #11**

I am looking at the revision already, seems to have undergone multiple rounds. Just a few comments

The word culture and cultural appeal seem a bit misleading. I first thought the paper is about culture in a sense of ethnicity ect., but itis about organization's or work culture. maybe worth to clarify what culture means here early on.

there is no real justification for the study. maybe better highlight that. i was thinking a bit So what? why was it really done. that could also be better reflected at the end in the section about practical considerations. There is not enough about policies and who could benefit from this study.

**Response(1):** We are deeply grateful for the constructive feedback provided in the previous rounds. In this final revision, we have meticulously refined the theoretical grounding and practical implications of our study to ensure disciplinary coherence and clarity for an international readership. We have consistently revised the focal construct throughout the manuscript to “Organizational Culture” and removed the term “cultural appeal” to prevent international readers from associating it with ethnic or national culture. Our study focuses exclusively on the “Organizational Culture”. While national culture changes slowly over generations, organizational culture is dynamic and can be shifted by leadership and management over shorter periods to meet strategic goals. By defining organizational culture as "how members relate to each other, to their work, and to the outside world," we clarify that our research is about institutional governance rather than ethnic characteristics. This distinction is critical for the generalizability of the findings to international contexts where different national cultures might still share similar organizational management challenges.

In terms of theoretical grounding, we introduced Edgar Schein’s organizational culture theory as the primary framework and incorporated seminal studies on healthcare organizational culture and emergency preparedness/response, thereby situating our theoretical development firmly within the domains of organizational behavior and health services management. The specific changes can be found in the revised manuscript: Introduction (page 4, lines 77–88).

**Response(2):** Regarding the study’s purpose, we rewrote the research motivation in the Introduction. We note that prior work on health emergency preparedness has predominantly emphasized the “hard” determinants of capacity—such as large hospitals, material infrastructure, and formalized procedures. In contrast, primary healthcare institutions function as frontline and terminal implementation units within the emergency system, yet they frequently operate under conditions of constrained resources, workforce shortages, and incomplete routines. Under such constraints, variation in emergency capacity may depend more heavily on organizational “soft infrastructure.” Accordingly, we argue that organizational culture constitutes a critical intangible resource in grassroots settings, shaping coordination efficiency, rule compliance, and mobilization capability when material resources are insufficient. Building on this premise, our study examines how organizational culture can partially compensate for limited infrastructure and thereby strengthen emergency capacity in resource-constrained primary healthcare contexts. The specific changes can be found in the revised manuscript: Introduction (page 6, lines 131–141).

**Response(3):** In response to the reviewer’s “So what?” concern, we revised the concluding synthesis based on our multi-group analysis and articulated the study’s central practical contribution more explicitly. Specifically, we advance an integrated perspective of “cultural empowerment and social-capital synergy” to guide the development of emergency capacity in primary healthcare institutions, directly addressing why the study matters. We also expanded the “Managerial Implications” section to clarify policy relevance and intended beneficiaries. We highlight that strengthening organizational culture provides a concrete pathway for translating the Healthy China strategy and professional ethos into grassroots practice. A "culture wall" in a primary healthcare center is a physical manifestation of "espoused beliefs" that fosters internal integration and a sense of shared purpose. Similarly, "Socialist Core Values" serve as a "soft constraint" and a carrier of specific cultural values that promote collaboration and discipline. By framing these through the lens of informal institutions and organizational behavior, we show how they contribute to a "safety-conscious work environment" and enhance the "disciplined atmosphere" necessary for effective emergency response. Then, for resource-constrained, aged communities, reinforcing structural social capital represents a key leverage point for addressing emergency-capacity shortfalls; for general communities, priority should be placed on cultivating relational ties. These measures are expected to benefit healthcare workers directly and, ultimately, improve outcomes for community residents by supporting evidence-informed “culture–emergency” integration strategies for primary healthcare institutions. The specific changes can be found in the revised manuscript: [Conclusion] (pages 32-33, lines 660–671) and [Managerial implications] (pages 35-37, lines 697–712, lines 722–732, lines 742–749).

**[Introduction] (page 6, lines 131–141) :** Consequently, focusing on primary healthcare institutions (PHIs), this study integrates organizational culture theory and social capital theory to achieve a threefold objective: (1) to evaluate the direct impacts of organizational culture and social capital on the health emergency capacity of PHIs; (2) to elucidate the mediating mechanism of social capital in the nexus between organizational culture and emergency capacity; and (3) to scrutinize whether these relationships exhibit heterogeneity across diverse community contexts. By addressing these objectives, this research contributes to the extant literature by offering theoretical insights and evidence-based strategies for augmenting the systemic resilience and emergency responsiveness of primary healthcare systems.

**[Conclusion] (pages 32-33, lines 669–681) :** Multi-group analyses further reveal community-level heterogeneity in these pathways: in general communities, relational and cognitive social capital exert significant effects, whereas in resource-constrained, older communities, structural social capital emerges as the pivotal mediator. The central practical contribution of this study is to demonstrate that strengthening primary healthcare institutions’ emergency capacity is not merely a matter of increasing resource inputs; rather, it is a systemic endeavor that hinges on the synergy between cultural enablement and social-capital mobilization. This perspective offers health authorities and institutional managers an alternative to conventional “hard-capacity” approaches by emphasizing the cultivation of shared values and internal cohesion, together with the strategically targeted development of external community networks. Such efforts can activate and amplify limited emergency resources, thereby translating them into substantive improvements in emergency response effectiveness.

**Reviewer #12**

1.Irrelevance of Gevorgyan & Manucharova (2015): This reference is a study within the fields of marketing and advertising. Its core independent variable, "Cultural Appeal," refers to rhetorical strategies (e.g., collectivism vs. individualism) used in advertising copy, and its dependent variables are consumer "brand recall" and "purchase attitudes." Equating a consumer's willingness to click on an advertisement in a low-stakes environment with a medical professional's emergency response behavior in a life-or-death situation presents a fundamental logical fallacy. Gevorgyan’s study focuses on Persuasion, whereas your study focuses on Mobilization. Advertising appeal primarily influences transient psychological preferences, whereas organizational cultural appeal operates on enduring professional responsibility. Citing an advertising paper to support a healthcare management theory is far-fetched and conceptually inappropriate.

**Response:** Thank you for this comment. We agree that citing Gevorgyan & Manucharova—which examines advertising persuasion mechanisms and consumer outcomes—is not conceptually aligned with our study of organizational mobilization and healthcare professionals’ emergency response in high-stakes settings. Accordingly, we have removed this citation and revised the surrounding text to avoid any implication that low-stakes advertising responses are comparable to emergency mobilization behaviors.

To strengthen disciplinary coherence, we revised the Introduction and the hypothesis development sections to ground our arguments in organizational culture theory, using Schein’s organizational culture perspective as an anchoring theoretical lens for explaining how shared values, norms, and underlying assumptions shape members’ motivation, coordination, and collective action during emergency response.

2.Misapplication of Michaels & Rettig (2025): This article belongs to the fields of environmental policy and political communication. The "culturally appealing climate frames" discussed in the text refer to how politicians utilize grand narratives (e.g., "Start-up Nation") to garner public support. You have cited this work to prove that cultural appeal acts as a positive driver for collective action. However, Michaels' research actually discusses the limitations and fragility of such drivers. Consequently, you have cited literature that may fundamentally contradict your own hypothesis.

**Response:** Thank you for this important comment. We agree that Michaels & Rettig , situated in environmental policy and political communication, is not conceptually appropriate to support our argument about healthcare emergency mobilization. Importantly, we also acknowledge that the paper discusses the limitations and fragility of culturally appealing frames, and therefore it should not be used as evidence that “cultural appeal” is a robust positive driver of collective action in our context. Accordingly, we have removed the citation and revised the surrounding text to eliminate the misplaced support for our hypothesis. We also revisited and expanded the literature review and strengthened Section 2.1 (“The impact of organizational culture on health emergency capacity”) by grounding the argument in organizational culture and healthcare emergency management scholarship that directly addresses high-stakes coordination, professional responsibility, and emergency preparedness. Where necessary, we clarified the wording and rationale of the hypotheses to ensure they are fully consistent with the healthcare emergency management context.

3. Issues with the "Cultural Appeal" Construct: "Cultural Appeal" is not a standard construct in international management literature. In the context of Western management science, the activities described in your manuscript—such as establishing "culture walls" and practicing "Socialist Core Values"—would more accurately map to concepts such as Organizational Culture, Safety Climate, or Institutional Logic. Persisting in the use of the non-standard term "Cultural Appeal" has forced you to cite irrelevant literature from advertising and political science, resulting in the aforementioned theoretical inconsistencies.

**Response:** In line with the editor’s and reviewers’ recommendations, we revised the focal construct and renamed it throughout the manuscript as “organizational culture.” This term more precisely captures what we measure—namely, the extent to which healthcare workers internalize and identify with the organizational culture cultivated by primary healthcare institutions through tangible artifacts (e.g., culture walls) and endorsed value systems (e.g., core values). We used this terminology consistently across the abstract, theoretical background, research model, measurement section, and discussion to prevent conceptual drift. Building on this clarified construct, we systematically revised the Introduction and hypothesis development, and removed prior citations drawn from non-aligned fields (e.g., advertising and political science) that were introduced under the earlier framing.

4.Critique of Terror Management Theory (TMT): The core premise of TMT is that "Mortality Salience" triggers "Worldview Defense." This defense often manifests as cognitive rigidity, exclusion, and irrational behavior, rather than the rational and efficient collaboration required in emergencies. Furthermore, since this study did not measure "death anxiety" or "defense mechanisms," applying TMT appears to be an over-interpretation unsupported by your data. It would be more appropriate to frame major public health events as a "Mortality Salience Context," where the psychological defense mechanisms of medical staff—as the group closest to death—are critical to maintaining their operational capacity.

**Response:** Thank you for this insightful comment. We agree that applying Terror Management Theory (TMT) in our Discussion was an over-interpretation because our study did not measure core TMT constructs (e.g., death anxiety, mortality-salience manipulation, or worldview-defense mechanisms). Accordingly, we have removed TMT as a theoretical explanation and deleted the related statements and citations. Following your recommendation, we now frame major public health emergencies as a mortality-salience context for healthcare workers, and we use this only as a contextual lens to interpret why organizational culture may matter under high-stakes conditions—without claiming to test TMT mechanisms. In the revised Discussion, we describe how shared organizational values and norms (e.g., responsibility, dedication, and collectivism) may support coordinated action during emergencies, and we have revised the wording to avoid unsupported causal or psychological-mechanism claims. We also replaced any remaining “cultural appeal” terminology with “organizational culture” to ensure conceptual consistency across the manuscript.

**[Discussion] (page 29, lines 564–580):** “This study demonstrates that organizational culture within primary healthcare institutions significantly enhances health emergency capacity. This influence is operationalized through a shared understanding of institutional missions and values, which function as internalized behavioral norms. Aligning with Wolf-Fordham, our results suggest that a robust, shared value orientation provides critical normative guidance and bolsters collective agency, thereby augmenting emergency response efficacy[53]. When healthcare personnel perceive organizational expectations as coherent and pervasive, it fosters heightened organizational identification and facilitates synchronized action—two precursors to efficient crisis management. Furthermore, major public health emergencies create a profound context of mortality salience, placing healthcare workers at the nexus of clinical risk and existential stress[54]. In such high-stakes environments, a shared mission serves as a psychological buffer and a mechanism for meaning-making. By mitigating negative affect and sustaining professional commitment, these cultural constructs ensure stable team coordination and optimize decision-making efficiency under pressure. Consequently, organizational culture acts not merely as a background variable but as a functional pillar of institutional resilience during health crises[55].”
