# Supplementary material for: Organizational culture, social capital, and emergency capacity in primary healthcare institutions: A cross-sectional structural equation modeling study comparing ordinary and older communities
Source: PLoS One. 2026 Jun 30;21(6):e0351875. doi: 10.1371/journal.pone.0351875 (PMC13318035; doi:10.1371/journal.pone.0351875)
Supplement: Attachment — Submitted filename: Response to Reviewers(1).doc [file pone.0351875.s014.doc]

**Point-by-point responses to the Reviewers’ comments**

**Academic Editor**

**1.Refines and streamlines the theoretical positioning, avoiding overstated claims of mechanism or contribution**

**Response:** Thank you for this important comment. We agree that the manuscript should not be positioned as establishing a definitive mechanism or making a broad theoretical breakthrough. In the revised manuscript, we have further reframed the study as a context-specific empirical examination of associations among organizational culture, social capital, and perceived health emergency capacity in primary healthcare institutions.

In the abstract and introduction, we have reformulated the research objective by shifting the emphasis away from “mechanism explanation” and toward testing the path relationships among organizational culture, social capital, and public health emergency capacity using a structural equation modeling approach. The revised manuscript now makes it clear that the focus of this study is to examine the structural relationships among variables and their statistical significance, rather than to offer a definitive explanation of causal mechanisms. The specific changes can be found in the revised manuscript: Abstract (page 2, lines 24-40), Introduction (pages 4-6, lines 83-86; page 11, lines 98-101).

In the hypothesis development sections, we have condensed and integrated the relevant content, removed some repetitive or overly extended theoretical descriptions, and strengthened the direct logical link between organizational culture and social capital. As a result, the theoretical framework is now more focused on the core variables and their relational pathways, thereby improving the overall conciseness and coherence of the manuscript. The specific changes can be found in the revised manuscript: Literature review and hypotheses (page 7, lines 144-152; page 8, lines 174-191；page 9, lines 203-207; page10, lines 214-217; page11, lines 233-240).

At the same time, we have toned down the wording of the theoretical contribution, revising stronger expressions such as “overcoming” and “innovation” to more measured formulations such as “complement existing literature” and “extend prior research.” Overall, the study is now positioned as providing empirical evidence rather than establishing a definitive mechanism. We hope these revisions enhance the rigor of the theoretical presentation and ensure better alignment with the research design. The specific changes can be found in the revised manuscript: Theoretical contribution (page 33, lines 660-667), Managerial implications (page 33, lines 674-680).

**[****Introduction] (page 2, lines 24-40) :** The emergency capacity of primary healthcare institutions is critical to the effectiveness of grassroots emergency management. This study examines the relationships among organizational culture, social capital, and the emergency capacity of primary healthcare institutions using a structural equation modeling approach. A questionnaire survey was conducted among healthcare professionals, yielding 983 valid responses for analysis. The results indicate that organizational culture, as well as structural, relational, and cognitive dimensions of social capital, are significantly associated with the emergency capacity of primary healthcare institutions within the model. In addition, social capital demonstrates mediating roles in the relationship between organizational culture and emergency capacity. Multi-group structural equation modeling further reveals variations across community types: relational social capital shows stronger associations with emergency capacity in ordinary communities, whereas structural social capital is more prominent in older communities. These findings provide empirical evidence on how organizational culture and social capital are linked to emergency capacity in primary healthcare settings, highlighting the importance of both internal cultural development and external social resources in different phases of emergency management.

**[Theoretical contribution] (page33, lines 658-673) :**This study demonstrated the significant influence of organizational culture and social capital on health emergency capacity, offering several theoretical contributions. By incorporating organizational culture into the analytical framework of emergency preparedness in primary healthcare institutions, this study examines its relationship with health emergency capacity, thereby extending existing perspectives that have predominantly focused on institutional arrangements, resources, and technical capabilities. Moreover, by comparing the differential roles of various types of social capital within the pathways linking organizational culture and emergency capacity, this study further extends the empirical research on the relationship between organizational culture and social capital. A further innovation lies in accounting for heterogeneity at the community level: we systematically tested how organizational culture and the three forms of social capital differentially affect health emergency capacity across community contexts. This community-type perspective—seldom employed in previous research—offers a novel lens for cultivating organizational culture and social capital in primary healthcare institutions according to local community characteristics.

**2. Revises the discussion to ensure that interpretations remain proportionate to the data**

**Response:** Thank you for the editor’s valuable suggestion. We have reorganized the structure of the Discussion section to ensure that each subsection is strictly aligned with the results of the structural equation modeling, thereby avoiding speculative interpretations that go beyond the empirical findings.

Specifically, Section 5.1 focuses on interpreting the results of the direct effects, including the path coefficients of organizational culture and different dimensions of social capital on public health emergency capacity. Section 5.2 discusses the differences in indirect effects among various forms of social capital based on the Bootstrap mediation analysis results. Section 5.3 provides a comparative analysis of the differences in path coefficients between general communities and aging communities, based on the multigroup SEM results.

During the revision process, we removed statements related to psychological mechanisms (e.g., mortality salience context and emotional buffering), as these constructs were not directly measured in this study and could otherwise lead to over-interpretation. In addition, we revised the interpretation of the multigroup SEM results by shifting from mechanism-oriented explanations to a more data-driven description of path differences based on Bootstrap results, emphasizing the varying strengths of indirect effects of social capital across different community types. We also refined the discussion of the implications derived from the multigroup analysis to avoid excessive inference beyond the empirical evidence. The specific changes can be found in the revised manuscript: Discussion (page 28, lines 555-559; page29, lines 571-576,580-598; page 31, lines 626-633).

**3. Clearly acknowledges design limitations and avoids causal language**

**Response:** Thank you for the editor’s valuable comments. We have further strengthened the description of the study’s design limitations and made consistent revisions throughout the manuscript to avoid presenting path results derived from cross-sectional structural equation modeling (SEM) as strict causal relationships. Specifically, the revisions are as follows:

(1) In the limitations section, we explicitly acknowledge the constraints of the cross-sectional design. Structural equation modeling is primarily used to test theoretical path relationships and assess model fit; therefore, it can provide evidence of associations between variables but does not support strict causal inference. The specific changes can be found in the revised manuscript: Research limitations and prospects (page 36, lines 727-734).

(2) In the discussion section, we have toned down expressions that may imply causality. Stronger terms such as “influence” “effect” and “promote” have been revised to more appropriate formulations consistent with cross-sectional SEM, such as “path relationship” “statistical association” “model-implied relationship” or “significant path relationship within the model”. The specific changes can be found in the revised manuscript: Discussion (page 28, lines 555-559; page29, lines 571-576,580-598; page 31, lines 626-633).

(3) In the conclusion section, we further avoided causal language that exceeds the scope of the research design. We emphasize that the findings primarily reflect the path relationships and variations specified in the theoretical model within the sample, rather than providing definitive confirmation of causal effects. The specific changes can be found in the revised manuscript: Research limitations and prospects (page 32, lines 636-656).

**[Research limitations and prospects] (page 36, lines 727-734) :** This study presents the following limitations. First, this study adopts a cross-sectional design, which captures data at a single point in time and limits the ability to establish temporal ordering or infer causal pathways among variables. Therefore, although structural equation modeling was employed to examine the path relationships among variables, the results should be interpreted as reflecting statistical associations and model-implied pathways rather than definitive causal relationships. Future research could employ longitudinal or intervention-based designs to further investigate temporal dynamics and potential causal pathways among these variables.

**[Conclusion] (page 32, lines 636-656) :** This study focuses on primary healthcare institutions and differs from much of the existing literature that emphasizes large hospitals or macro-level emergency systems. From the perspective of community-based, grassroots healthcare service settings, it examines the path relationships among variables associated with health emergency capacity. This perspective provides additional empirical evidence that contributes to the literature on emergency preparedness at the primary healthcare level. The results indicate that organizational culture in primary healthcare institutions is positively associated with health emergency capacity both directly and indirectly through different forms of social capital, with the pathway via relational social capital exhibiting the strongest effect. Multi-group analysis further reveals heterogeneity across community contexts: in ordinary communities, relational and cognitive social capital show significant associations with health emergency capacity, whereas in resource-constrained older communities, the mediating role of structural social capital is more pronounced. These findings suggest that improvements in the emergency capacity of primary healthcare institutions are not only related to resource inputs but are also associated with organizational culture and social capital. From a practical perspective, the results provide a complementary “soft-capacity” lens for health authorities and institutional managers, highlighting the potential value of fostering shared values and internal cohesion, alongside the targeted development of social capital, to support the effective utilization of emergency resources and improve emergency capacity at the primary healthcare level.

**Reviewer #13:**

The manuscript presents a study examining the relationship between organizational culture, social capital, and emergency response performance using SEM on a relatively strong sample size (N=983), and while the title and abstract are reasonably clear and informative, they remain somewhat overloaded with variables and read more like a technical summary than a compelling research narrative; the introduction has improved compared to earlier versions by clarifying the motivation and focusing on healthcare systems, yet it still lacks a strong and naturally convincing research gap, giving the impression of a constructed rather than inherent contribution; the literature review and theoretical framework represent the weakest component of the study, as the initial conceptual confusion (particularly the use of “cultural appeal”) and inconsistent theoretical grounding were only partially resolved by shifting to organizational culture, resulting in a framework that feels retrofitted rather than conceptually robust; the methodology is generally acceptable, supported by an adequate sample size, appropriate use of SEM, and clear ethical considerations, but it is limited by its cross-sectional design, reliance on self-reported data, and insufficient depth in capturing underlying psychological or organizational mechanisms; the results are statistically sound and clearly presented, with some added value from multi-group analysis, yet they remain predictable and offer limited theoretical insight; the discussion has improved by removing inappropriate theoretical applications such as Terror Management Theory and aligning interpretations more closely with the data, though some degree of over-interpretation persists; the conclusion and implications provide practical relevance for healthcare management and policy, but tend to overstate the contribution given the modest originality of the findings; overall, despite strengths in data size, methodological execution, and applied relevance, the manuscript suffers from fundamental conceptual instability, limited novelty, and a theoretical foundation that appears reactive rather than well-developed, leading to the final recommendation of rejection (borderline major revision), as the core issues are too significant to justify further rounds of revision at this stage.

**Response:** Thank you for this careful and critical assessment. We have taken your concerns seriously and have revised the manuscript to better match the actual strength and scope of the study. We agree that the manuscript should not be presented as a theory-building paper or as establishing a deep causal mechanism. Accordingly, we have repositioned the manuscript as a context-specific empirical study of associations among organizational culture, social capital, and perceived emergency capacity in primary healthcare institutions. Our revisions address your concerns in the following ways:

(1) We revised the title and abstract to reduce variable overload and to make the study design clearer. The revised title now explicitly identifies the study as a cross-sectional SEM study comparing ordinary and older communities. The abstract now emphasizes the empirical setting, the cross-sectional design, and the interpretation of the results as model-implied associations. The specific changes can be found in the revised manuscript: Abstract (page 2, lines 24-40).

(2) In the introduction, we refined the presentation of the research gap to make it more natural and focused. The revised text now emphasizes that prior research on PHI emergency capacity has primarily concentrated on formal resources and managerial arrangements, while paying less attention to internal organizational culture and community-embedded social capital, and rarely examining their roles within a unified analytical framework. The specific changes can be found in the revised manuscript: Introduction (pages 4-6, lines 83-86; page 11, lines 98-101).

(3) Regarding the literature review, we agree that this section required a stronger conceptual logic. We therefore simplified the hypothesis development section and concentrated on a narrower and more coherent set of arguments. Overly broad or weakly connected theoretical claims were removed, and the transition to the hypotheses was rewritten to clarify that the framework serves as a basis for examining associative relationships, rather than claiming to validate deep causal mechanisms. The specific changes can be found in the revised manuscript: Literature review and hypotheses (page 7, lines 144-152; page 8, lines 174-191；page 9, lines 203-207; page10, lines 214-217; page11, lines 233-240).

(4) In response to your concern regarding conceptual ambiguity, we further simplified the conceptualization of organizational culture. We removed language that could imply conceptual drift or overextension. Organizational culture is now more directly defined in terms of shared values, norms, mission orientation, and role expectations within PHI. We also eliminated elements that could be interpreted as conceptually redundant or insufficiently grounded in this study.

(5) In the theoretical contribution section, we no longer claim major theoretical innovation. Instead, we emphasize three more constrained contributions: introducing organizational culture into this empirical context, distinguishing different dimensions of social capital, and identifying community-type heterogeneity in associative patterns. We also toned down the wording throughout the manuscript by replacing stronger expressions such as “innovation” and “mechanism identification” with more appropriate terms such as “providing empirical evidence” and “testing theoretical path relationships”. The study is now positioned as a complement and extension of existing literature rather than a theoretical breakthrough, with greater emphasis on empirical value and contextual relevance rather than theoretical novelty. The specific changes can be found in the revised manuscript: Theoretical contribution (page 33, lines 660-667).

(6) In the discussion and limitations sections, all interpretations are strictly aligned with the SEM results (direct effects, indirect effects, and multigroup analysis). Mechanism-oriented interpretations have been replaced with explanations grounded in observed path results to avoid inferences beyond the data. Furthermore, we have strengthened the discussion of study limitations by explicitly noting that the analysis is based on cross-sectional data and that SEM is primarily used to test theoretical path relationships and model structure. We emphasize that the findings reflect path associations within the model rather than definitive confirmation of causal mechanisms, and we further acknowledge limitations related to data sources and measurement methods. The specific changes can be found in the revised manuscript: Discussion (page 28, lines 555-559; page29, lines 571-576,580-598; page 31, lines 626-633), and Research limitations and prospects (page 36, lines 727-734).

**Reviewer #14:**

Thank you for submitting the updated version of the manuscript. I think that the paper is well-written and addresses a valuable and relevant gap. You have also addressed the comments that you were given in a proper manner. Minor issue: Please make sure to fix the referencing error in line 336.

**Response:** Thank you for pointing this out. We have carefully checked and corrected the relevant referencing error and also conducted an additional review of the in-text citations and reference list to improve consistency and accuracy throughout the manuscript.

**Reviewer #14:**

After several rounds of revisions, the authors have effectively addressed the shortcomings pointed out by the reviewers.

**Response:** Thank you very much for your encouraging evaluation. We appreciate your positive assessment of the revisions and are grateful for your guidance throughout the review process.
