# Supplementary material for: Organizational culture, social capital, and emergency capacity in primary healthcare institutions: A cross-sectional structural equation modeling study comparing ordinary and older communities
Source: PLoS One. 2026 Jun 30;21(6):e0351875. doi: 10.1371/journal.pone.0351875 (PMC13318035; doi:10.1371/journal.pone.0351875)
Supplement: Peer review history

# Peer review: Decision Letter 0

**DOI:** 10.1371/journal.pone.0351875.r001

**Authors:** Weicong Li

28 Jan 2025

PONE-D-24-36223

How do cultural appeal and social capital differ in influencing primary health care emergency capabilities in ordinary versus older communities?

-->PLOS ONE

Dear Dr. Zhang,

Thank you for submitting your manuscript to PLOS ONE. After careful consideration, we feel that it has merit but does not fully meet PLOS ONE’s publication criteria as it currently stands. Therefore, we invite you to submit a revised version of the manuscript that addresses the points raised during the review process.

Please note that I have acted as a reviewer for this manuscript, and you will find my comments below, under **Reviewer 3** .

**Comments from the editorial office:**  Upon internal evaluation of the reviews provided, we kindly request you to disregard the reviewer report provided by Reviewer 1. No amendments are required in response to reviewer 1’s comments

Please submit your revised manuscript by Mar 09 2025 11:59PM. If you will need more time than this to complete your revisions, please reply to this message or contact the journal office at plosone@plos.org. When you're ready to submit your revision, log on to https://www.editorialmanager.com/pone/ and select the 'Submissions Needing Revision' folder to locate your manuscript file.

Please include the following items when submitting your revised manuscript:

- A rebuttal letter that responds to each point raised by the academic editor and reviewer(s). You should upload this letter as a separate file labeled 'Response to Reviewers'.
- A marked-up copy of your manuscript that highlights changes made to the original version. You should upload this as a separate file labeled 'Revised Manuscript with Track Changes'.
- An unmarked version of your revised paper without tracked changes. You should upload this as a separate file labeled 'Manuscript'.

If you would like to make changes to your financial disclosure, please include your updated statement in your cover letter. Guidelines for resubmitting your figure files are available below the reviewer comments at the end of this letter.

If applicable, we recommend that you deposit your laboratory protocols in protocols.io to enhance the reproducibility of your results. Protocols.io assigns your protocol its own identifier (DOI) so that it can be cited independently in the future. For instructions see: https://journals.plos.org/plosone/s/submission-guidelines#loc-laboratory-protocols. Additionally, PLOS ONE offers an option for publishing peer-reviewed Lab Protocol articles, which describe protocols hosted on protocols.io. Read more information on sharing protocols at https://plos.org/protocols?utm_medium=editorial-email&utm_source=authorletters&utm_campaign=protocols.

We look forward to receiving your revised manuscript.

Kind regards,

Weicong Li, P.hD

Academic Editor

PLOS ONE

Journal Requirements:

When submitting your revision, we need you to address these additional requirements.

1. Please ensure that your manuscript meets PLOS ONE's style requirements, including those for file naming. The PLOS ONE style templates can be found at

https://journals.plos.org/plosone/s/file?id=wjVg/PLOSOne_formatting_sample_main_body.pdf and

https://journals.plos.org/plosone/s/file?id=ba62/PLOSOne_formatting_sample_title_authors_affiliations.pdf

2. We suggest you thoroughly copyedit your manuscript for language usage, spelling, and grammar. If you do not know anyone who can help you do this, you may wish to consider employing a professional scientific editing service.

The American Journal Experts (AJE) (https://www.aje.com/) is one such service that has extensive experience helping authors meet PLOS guidelines and can provide language editing, translation, manuscript formatting, and figure formatting to ensure your manuscript meets our submission guidelines. Please note that having the manuscript copyedited by AJE or any other editing services does not guarantee selection for peer review or acceptance for publication.

Upon resubmission, please provide the following:

The name of the colleague or the details of the professional service that edited your manuscript

A copy of your manuscript showing your changes by either highlighting them or using track changes (uploaded as a *supporting information* file)

A clean copy of the edited manuscript (uploaded as the new *manuscript* file)

3. We note that the grant information you provided in the ‘Funding Information’ and ‘Financial Disclosure’ sections do not match.

When you resubmit, please ensure that you provide the correct grant numbers for the awards you received for your study in the ‘Funding Information’ section.

4. Thank you for stating the following financial disclosure:

"This research was funded  the Philosophy and Social Science Project of Zhejiang Province [grant number 23YJRC11ZD-3YB]."

Please state what role the funders took in the study.  If the funders had no role, please state: ""The funders had no role in study design, data collection and analysis, decision to publish, or preparation of the manuscript.""

If this statement is not correct you must amend it as needed.

Please include this amended Role of Funder statement in your cover letter; we will change the online submission form on your behalf.

5. Thank you for stating the following in your Competing Interests section:

"NO authors have competing interests."

Please complete your Competing Interests on the online submission form to state any Competing Interests. If you have no competing interests, please state ""The authors have declared that no competing interests exist."", as detailed online in our guide for authors at http://journals.plos.org/plosone/s/submit-now

This information should be included in your cover letter; we will change the online submission form on your behalf.

6. In the online submission form, you indicated that [The raw data supporting the conclusions of this article will be made available by the corresponding author upon request.].

All PLOS journals now require all data underlying the findings described in their manuscript to be freely available to other researchers, either 1. In a public repository, 2. Within the manuscript itself, or 3. Uploaded as supplementary information.

This policy applies to all data except where public deposition would breach compliance with the protocol approved by your research ethics board. If your data cannot be made publicly available for ethical or legal reasons (e.g., public availability would compromise patient privacy), please explain your reasons on resubmission and your exemption request will be escalated for approval.

7. PLOS requires an ORCID iD for the corresponding author in Editorial Manager on papers submitted after December 6th, 2016. Please ensure that you have an ORCID iD and that it is validated in Editorial Manager. To do this, go to ‘Update my Information’ (in the upper left-hand corner of the main menu), and click on the Fetch/Validate link next to the ORCID field. This will take you to the ORCID site and allow you to create a new iD or authenticate a pre-existing iD in Editorial Manager.

8. We notice that your supplementary files are uploaded with the file type 'Other'. Please amend the file type to 'Supporting Information'. Please ensure that each Supporting Information file has a legend listed in the manuscript after the references list.

Additional Editor Comments (if provided):

In addition to addressing the reviewers' comments, the authors need to resolve the following issues:

1.The abstract is too long and contains repetitive content, failing to effectively summarize the research objectives and key findings.

2.There is insufficient justification explaining the selection of the four cities as samples, the rationale behind this choice, and whether these cities adequately 3.represent the development level of elderly communities in the region.

3.Please provide definitions of the terms in the title as per the reviewers' suggestions.

4.Add a section to present the research hypotheses and explain the relationships between the variables.

5.Clarify the validity of the structural equation modeling.

6.In the author information section, identical institutional affiliations should appear only once.

[Note: HTML markup is below. Please do not edit.]

Reviewers' comments:

Reviewer's Responses to Questions

**Comments to the Author**

1. Is the manuscript technically sound, and do the data support the conclusions?

The manuscript must describe a technically sound piece of scientific research with data that supports the conclusions. Experiments must have been conducted rigorously, with appropriate controls, replication, and sample sizes. The conclusions must be drawn appropriately based on the data presented.

Reviewer #1: Partly

Reviewer #2: Partly

Reviewer #3: Partly

Reviewer #4: Yes

Reviewer #5: Yes

**********

2. Has the statistical analysis been performed appropriately and rigorously?

Reviewer #1: N/A

Reviewer #2: I Don't Know

Reviewer #3: N/A

Reviewer #4: Yes

Reviewer #5: Yes

**********

3. Have the authors made all data underlying the findings in their manuscript fully available?

The PLOS Data policy requires authors to make all data underlying the findings described in their manuscript fully available without restriction, with rare exception (please refer to the Data Availability Statement in the manuscript PDF file). The data should be provided as part of the manuscript or its supporting information, or deposited to a public repository. For example, in addition to summary statistics, the data points behind means, medians and variance measures should be available. If there are restrictions on publicly sharing data—e.g. participant privacy or use of data from a third party—those must be specified.

Reviewer #1: No

Reviewer #2: Yes

Reviewer #3: No

Reviewer #4: No

Reviewer #5: Yes

**********

4. Is the manuscript presented in an intelligible fashion and written in standard English?

PLOS ONE does not copyedit accepted manuscripts, so the language in submitted articles must be clear, correct, and unambiguous. Any typographical or grammatical errors should be corrected at revision, so please note any specific errors here.

Reviewer #1: Yes

Reviewer #2: No

Reviewer #3: Yes

Reviewer #4: No

Reviewer #5: Yes

**********

5. Review Comments to the Author

Please use the space provided to explain your answers to the questions above. You may also include additional comments for the author, including concerns about dual publication, research ethics, or publication ethics. (Please upload your review as an attachment if it exceeds 20,000 characters)

Reviewer #1: 1. Abstract lacks conciseness and has repetitive wording.The abstract is lengthy and contains redundant expressions, failing to concisely summarize the study and key findings. It is recommended to streamline the language and reduce repetition to improve readability.

2. Unclear sampling rationale.The study selected four cities in Zhejiang Province as random samples, but the rationale and representativeness of this selection are not clearly explained. The authors should provide a detailed explanation of the logic behind the selection and clarify whether these cities adequately represent the development level of elderly communities in the region.

3. Institutional affiliations are not properly standardized.The affiliations of the authors are listed multiple times for the same institution. It is recommended to merge relevant information to ensure the institutional listings are properly standardized.

4. Unclear hypothesis and variable relationships.Before the "General Information Survey" section, an additional section should be added to clearly present the study's hypotheses and explain the relationships between variables, such as identifying independent, dependent, and qualitative variables, to enhance the clarity of the research framework.

Reviewer #2: .

Reviewer #3: 1. Please define the concepts of 'cultural appeal' and 'social capital' at the beginning of the 'Introduction' section. Please define the concepts of 'cultural appeal' and 'social capital' at the beginning of the 'Introduction' section to narrow the scope. Introduce what they consist of.

2.Similarly, what is meant by 'primary health care emergency capabilities'? This should also be described in detail here.

3. An additional expert is needed to test the structural equations and give a professional opinion.

Reviewer #4: Please pay special attention to the research ethics issues we have mentioned. The approval of the Institutional Review Committee (approval number is provided) and the written informed consent process of the participants described in this study have strictly followed the relevant ethical guidelines. We ensured that the rights of the participants in the study were protected, including their right to withdraw at any time and the confidentiality of their information. We kindly ask you to verify the implementation of these ethical compliance measures during the review process to ensure the ethical and legal rigor of this study. Thank you for your careful review.

Reviewer #5: 1. The article does not include a statement regarding conflicts of interest.

2. It is recommended to specify how this study addresses gaps in existing literature, such as the unique contribution of quantitatively analyzing the role of "cultural appeal" and "social capital" in health emergency capacity.

3.Some citations in the text are incorrectly formatted, and some references are incomplete.

4. The article mentions "ordinary" and "older" communities, but lacks a clear definition of the criteria used to classify these communities.

5. The data analysis section mentions using SPSS and AMOS for statistical analysis, but lacks more detailed explanation of the specific methods employed. It is suggested to improve the transparency of data analysis.

6. In the discussion of results, a deeper discussion of the effect sizes would be beneficial. It is recommended to evaluate which variables have more significant effects, which have weaker effects, and the practical significance of these effects.

7. The discussion of community type differences and their underlying causes should be more detailed, especially in terms of how socio-economic backgrounds relate to the formation of cultural appeal and social capital. For example, older communities may face challenges such as inadequate infrastructure and population aging—how do these factors influence the interaction between cultural appeal and social capital?

**********

6. PLOS authors have the option to publish the peer review history of their article (what does this mean?). If published, this will include your full peer review and any attached files.

If you choose “no”, your identity will remain anonymous but your review may still be made public.

**Do you want your identity to be public for this peer review?**  For information about this choice, including consent withdrawal, please see our Privacy Policy.

Reviewer #1: No

Reviewer #2: No

Reviewer #3: No

Reviewer #4: No

Reviewer #5: **Yes:**  Yi-Tong Cui

**********

-->[NOTE: If reviewer comments were submitted as an attachment file, they will be attached to this email and accessible via the submission site. Please log into your account, locate the manuscript record, and check for the action link "View Attachments". If this link does not appear, there are no attachment files.]

While revising your submission, please upload your figure files to the Preflight Analysis and Conversion Engine (PACE) digital diagnostic tool, https://pacev2.apexcovantage.com/. PACE helps ensure that figures meet PLOS requirements. To use PACE, you must first register as a user. Registration is free. Then, login and navigate to the UPLOAD tab, where you will find detailed instructions on how to use the tool. If you encounter any issues or have any questions when using PACE, please email PLOS at figures@plos.org. Please note that Supporting Information files do not need this step.

# Author comment: Author response to Decision Letter 1

**DOI:** 10.1371/journal.pone.0351875.r002

3 Mar 2025

March 2, 2025

Weicong Li, PhD

Academic Editor

PLOS ONE

RE: “How do cultural appeal and social capital differ in influencing primary healthcare emergency capabilities in ordinary versus older communities?” Manuscript ID: PONE-D-24-36223

Dear Editors:

Thank you for the opportunity to further revise our manuscript “How do cultural appeal and social capital differ in influencing primary healthcare emergency capabilities in ordinary versus older communities?” The reviewers’ and editors’ comments were useful and helped to clarify and improve several aspects of the manuscript.

Please find attached the revised version of the manuscript; the changes made are indicated using red font. Below is a point-by-point response to the issues raised by the reviewers and editors. We hope that this version is suitable for publication in PLOS ONE.

We look forward to hearing from you.

Dr. Xiaoting Zhang

School of Public Health, Hangzhou Normal University

Hangzhou 311121, China

zxtbing@hznu.edu.cn

Point-by-point response.

Editorial

1. The abstract is too long and contains repetitive content, failing to effectively summarize the research objectives and key findings.

Response: Thank you for your valuable comments on our manuscript. In the revised version, we have eliminated redundant and repetitive content, ensuring that the abstract is clearer and more concise. Additionally, we have summarized the research findings, presenting the direct and indirect effects of cultural influence and social capital in the results section based on the structural equation model and emphasizing the path differences between old and ordinary communities.

[Abstract] (page 2, lines 33–47): “A questionnaire survey was conducted among healthcare professionals, with 983 valid responses collected for analysis. Structural equation modeling was employed for analysis, which revealed that cultural appeal, structural social capital, relational social capital, and cognitive social capital directly influence the health emergency capacity of grassroots healthcare institutions. All three types of social capital moderate the relationship between cultural appeal and health emergency capacity. Furthermore, multi-group structural equation modeling analyses by community type showed that, compared to the general community group, the direct effect of cultural appeal on health emergency capacity in the old community group was larger than the indirect effect. These findings highlight the crucial role of organizational culture development and the social capital elements embedded in social structures—such as social relationships, network participation, trust, and norms—in the prevention, preparation, response, and recovery phases of emergency management in grassroots healthcare institutions.”

2. There is insufficient justification explaining the selection of the four cities as samples, the rationale behind this choice, and whether these cities adequately 3. represent the development level of elderly communities in the region.

Response: We appreciate your valuable feedback, and we apologize for the lack of clarity in our original manuscript. We have now provided a clearer explanation of the selection criteria of the four cities, as outlined below:

First, the selection was based on the most recent gross domestic product rankings of the 11 cities in Zhejiang Province for 2022, alongside regional economic and public finance indicators. We categorized the 11 cities into four tiers according to their economic levels. Using random sampling, we selected Hangzhou, Jiaxing, Huzhou, and Lishui. These cities represent a spectrum of development levels, from high to low, ensuring that our study captures a broad range of spatial and economic contexts and thus enhancing the diversity and representativeness of the findings.

Second, we considered whether these cities adequately reflect the development levels of old communities in the region. In the [Methods] section, we added a detailed explanation regarding the development levels of old communities in the selected cities to strengthen the justification for our sample. For instance, Hangzhou, as the most economically advanced city in Zhejiang Province, boasts well-developed infrastructure in its old communities, ample healthcare resources, and strong emergency-management capabilities in primary healthcare institutions. These differences allow the selected cities to effectively represent the varying development levels of old communities across Zhejiang, thereby ensuring that our results are both broadly applicable and representative. Furthermore, we have included statistical data obtained from the Housing and Urban-Rural Development Bureau of each city in Zhejiang Province to support the basis for our city selection. Through these clarifications, we aim to more clearly highlight the representativeness of the chosen cities and enhance the overall credibility of our study.

[Methods] (page 11-13, lines 258–284): “The cities were categorized into four levels according to their economic status considering regional economic indicators and public finance data. The first level includes Hangzhou and Ningbo, the two cities with a total GDP exceeding one trillion RMB. The second level comprises Wenzhou, Shaoxing, Jiaxing, and Taizhou, which have a GDP exceeding 500 billion RMB. The third level includes Jinhua and Huzhou with a GDP between 300 billion and 500 billion RMB. The fourth level comprises Quzhou, Lishui, and Zhoushan, which have a GDP not exceeding 200 billion RMB. Random sampling was used to select one city from each level: Hangzhou, Jiaxing, Huzhou, and Lishui. These cities exhibit considerable differences in the development of old neighborhoods and can provide a comprehensive reflection of the overall situation in Zhejiang Province. As the most economically developed city in Zhejiang Province, Hangzhou has relatively well-developed infrastructure in its old neighborhoods, ample medical resources, and strong emergency-management capabilities within grassroots healthcare institutions. Since the launch of the old neighborhood renovation program in 2019, Hangzhou has been ranked first for three consecutive years. The city focuses on integrated planning of public service facilities and community healthcare service networks, significantly enhancing the emergency response capabilities of grassroots communities and healthcare institutions. Jiaxing and Huzhou, while economically more developed, still face some gaps in infrastructure and public service resource allocation compared to Hangzhou. As of September 2022, Jiaxing had completed the renovation of 491 old neighborhoods, constructed 651,200 square meters of urban community service centers, and established 30 community health service centers [43]. By November 2022, Huzhou had included 89 neighborhoods in its renovation plan [44]. Lishui faces significant challenges in its old neighborhoods, including aging infrastructure and insufficient medical resources. The progress of its old neighborhood renovation has been relatively slow compared to the other three cities.”

3. Please provide definitions of the terms in the title as per the reviewers' suggestions.

Response: We appreciate your comments as well as the reviewers' suggestions. Regarding the definitions of the terms in the title, we have provided a comprehensive explanation in response to the reviewers' feedback and have included precise definitions of the relevant terms within the manuscript. We trust that these definitions will effectively clarify the terms used in the title and enhance readers' understanding of the study's content and context.

4. Add a section to present the research hypotheses and explain the relationships between the variables.

Response: We appreciate your valuable feedback on the paper. We apologize for the previous lack of clarity in presenting the hypotheses, which may have made it difficult for readers to understand them. We have now revised the research hypotheses section and provided a detailed explanation of the relationships between the variables [Literature review and hypotheses] (page 6, lines 143–251).

5. Clarify the validity of the structural equation modeling.

Response: We greatly appreciate your insightful comments and suggestions. In response, we have revised the manuscript to better address your concerns. Specifically, we have provided a more detailed discussion of the theoretical foundation of structural equation modeling (SEM) and highlighted its advantages in addressing complex causal relationships. We also explain why SEM was chosen as the most appropriate method for this study. SEM is a multivariate statistical technique based on the covariance matrix of variables. It is widely used in factor and path analysis, as it allows for the exploration of relationships between multiple variables and provides a comprehensive understanding of their interactions. SEM is commonly applied in fields such as psychology and sociology, making it particularly well-suited for our research.

Furthermore, the SEM framework consists of two components: the measurement model and the structural model. The measurement model defines how latent variables (unobserved constructs) are represented by observed indicators (manifest variables), while the structural model assesses the relationships between these latent variables. In this study, the latent variables (represented by ellipses) include cultural influence, structural social capital, cognitive social capital, and relational social capital. These are measured by the observed variables (represented by rectangles), which are detailed in the manuscript.

We used a sample of 983 responses and conducted reliability and validity tests on the scales. We then employed AMOS 26 software to build and analyze the SEM to test the research hypotheses. For the validity analysis, we used confirmatory factor analysis to assess the appropriateness of the measurement model. The results showed that the model fit was satisfactory, and we confirmed the composite reliability, convergent validity, and discriminant validity. No issues were found with the composite reliability or average variance extracted. Finally, we provided the fit indices for the structural model; goodness of fit indices (goodness of fit index, adjusted goodness of fit index, normal fit index, comparative fit index, Tucker Lewis index) exceeded 0.9, and the root mean square of approximation and its 90% confidence interval were less than 0.08, indicating that the model exhibits good overall fit.

[Statistical analyses] (page 17, lines 385–390): “SEM is a multivariate statistical technique that utilizes the covariance matrix of variables for both factor and path analysis. It is designed to investigate the latent relationships among multiple variables and to provide a comprehensive analysis of their interactive mechanisms. SEM is extensively used in disciplines such as psychology and sociology, making it an appropriate choice for the present study.”

6. In the author information section, identical institutional affiliations should appear only once

Response: We appreciate your suggestion. In response, we have revised the author information section to consolidate identical institutional affiliations, ensuring that each institution is listed only once, as per the journal’s guidelines. The repetition of “Department of Health Policy and Management, School of Public Health, Hangzhou Normal University” has been removed with only one mention retained, while the other distinct institutions remain unchanged.

Reviewer #2:

None

Reviewer #3:

1. Please define the concepts of 'cultural appeal' and 'social capital' at the beginning of the 'introduction' section, Please define the concepts of' cultural appeal' and 'social capital at the beginning of the Introduction' section to narrow the scope. Introduce what they consist of.

Response: Thank you for your valuable suggestions. Based on your feedback, we have clarified the concepts of “cultural attractiveness” and “social capital” at the beginning of the [Introduction] section and provided a more detailed description of their components. Through these definitions and explanations, we aim to better define the scope of the study and clearly present the core meanings of “cultural attractiveness” and “social capital.”

[Introduction] (page 4, lines 84–86): “Cultural appeal refers to the ability of societal mainstream values and a rational spirit to influence and inspire individuals. It encompasses the traditions, values, organizational goals, and cultural norms passed down across generations.”

[Introduction] (page 4-5, lines 95–101): “social capital comprises resources embedded in social networks, which enhance social efficiency and generate added value by facilitating coordination and action among participants. Social networks, participation, trust, reciprocity, and norms are widely recognized as key elements of social capital. Regarding the classification of social capital, the discussion of a framework encompassing three dimensions—structural, cognitive, and relational—has gained popularity since 2004, and this framework has become the most used and widely accepted approach.”

2. Similarly, what is meant by 'primary health care emergency capabilities'? This should also be described in detail here.

Response: We greatly appreciate your valuable suggestions. In response to your feedback, we reviewed the literature on emergency management in public health emergencies and found that the Prevention, Preparation, Response, and Recovery framework and the crisis lifecycle theory are widely applied in conceptualizing emergency management capacity and in developing related indicators. Drawing upon the findings from various studies, we have clarified the concept of “health emergency capacity in grassroots healthcare institutions” at the beginning of the [Introduction] section and further elaborated on its components.

[Introduction] (page 3, lines 56–61): “The health emergency capacity of primary healthcare institutions refers to their comprehensive ability to mobilize resources and implement effective responses related to emergency preparedness, monitoring and early warning, emergency response, and post-event recovery to prevent and address sudden public health incidents. This includes capacities for emergency prevention, emergency preparedness, emergency response, and post-disaster recovery .”

3. An additional expert is needed to test the structural equations and give a professional opinion.

Response: We appreciate your insightful suggestion regarding the validation of our structural equation model. In response, we have engaged an independent expert in statistical modeling to conduct a comprehensive evaluation of our analytical framework. The expert's rigorous assessment corroborates the robustness and statistical validity of our model specifications.

Reviewer #4:

Please pay special attention to the research ethics issues we have mentioned. The approval of the Institutional Review Committee (approval number is provided) and the written informed consent process of the participants described in this study have strictly followed the relevant ethical guidelines. We ensured that the rights of the participants in the study were protected, including their right to withdraw at any time and the confidentiality of their information. We kindly ask you to verify the implementation of these ethical compliance measures during the review process to ensure the ethical and legal rigor of this study. Thank you for your careful review.

Response: We sincerely appreciate your valuable suggestion. Our research has been conducted in strict compliance with relevant ethical guidelines and has obtained formal approval from the Institutional Review Committee. We have uploaded our Chinese ethical approval document and its English translation in the system. Furthermore, we have implemented a comprehensive written informed consent procedure that fully adheres to established ethical standards and regulatory requirements.

Reviewer #5:

1. The article does not include a statement regarding conflicts of interest.

Response: Thank you for your careful review and for pointing out this oversight. We ha

# Peer review: Decision Letter 1

**DOI:** 10.1371/journal.pone.0351875.r003

**Authors:** Chunyu Zhang

16 Apr 2025

PONE-D-24-36223R1

How do cultural appeal and social capital differ in influencing primary health care emergency capabilities in ordinary versus older communities?

PLOS ONE

Dear Dr. Zhang,

Thank you for submitting your manuscript to PLOS ONE. After careful consideration, we feel that it has merit but does not fully meet PLOS ONE’s publication criteria as it currently stands. Therefore, we invite you to submit a revised version of the manuscript that addresses the points raised during the review process.

I'm very sorry for the late decision. When this manuscript was handed over to me, two peer reviewers decided to accept it. I have read it, and I think it is not yet of a quality that would publication. Therefore, I have invited two additional peer reviewers.

1. What is the practical contribution of the research?

2. The first chapter has too many paragraphs and the logic is confused.

3. All hypotheses need to be rewritten. The current research hypothesis reads less like an academic description than a news report.

4. The discussion section needs to distinguish between academic and practical.

5.  The references do not meet journal standards.

Please submit your revised manuscript by May 31 2025 11:59PM. If you will need more time than this to complete your revisions, please reply to this message or contact the journal office at plosone@plos.org. When you're ready to submit your revision, log on to https://www.editorialmanager.com/pone/ and select the 'Submissions Needing Revision' folder to locate your manuscript file.

Please include the following items when submitting your revised manuscript:

- A rebuttal letter that responds to each point raised by the academic editor and reviewer(s). You should upload this letter as a separate file labeled 'Response to Reviewers'.
- A marked-up copy of your manuscript that highlights changes made to the original version. You should upload this as a separate file labeled 'Revised Manuscript with Track Changes'.
- An unmarked version of your revised paper without tracked changes. You should upload this as a separate file labeled 'Manuscript'.

If you would like to make changes to your financial disclosure, please include your updated statement in your cover letter. Guidelines for resubmitting your figure files are available below the reviewer comments at the end of this letter.

If applicable, we recommend that you deposit your laboratory protocols in protocols.io to enhance the reproducibility of your results. Protocols.io assigns your protocol its own identifier (DOI) so that it can be cited independently in the future. For instructions see: https://journals.plos.org/plosone/s/submission-guidelines#loc-laboratory-protocols. Additionally, PLOS ONE offers an option for publishing peer-reviewed Lab Protocol articles, which describe protocols hosted on protocols.io. Read more information on sharing protocols at https://plos.org/protocols?utm_medium=editorial-email&utm_source=authorletters&utm_campaign=protocols.

We look forward to receiving your revised manuscript.

Kind regards,

Chunyu Zhang

Academic Editor

PLOS ONE

[Note: HTML markup is below. Please do not edit.]

Reviewers' comments:

Reviewer's Responses to Questions

**Comments to the Author**

1. If the authors have adequately addressed your comments raised in a previous round of review and you feel that this manuscript is now acceptable for publication, you may indicate that here to bypass the “Comments to the Author” section, enter your conflict of interest statement in the “Confidential to Editor” section, and submit your "Accept" recommendation.

Reviewer #3: All comments have been addressed

Reviewer #4: All comments have been addressed

Reviewer #5: All comments have been addressed

Reviewer #6: All comments have been addressed

**********

2. Is the manuscript technically sound, and do the data support the conclusions?

The manuscript must describe a technically sound piece of scientific research with data that supports the conclusions. Experiments must have been conducted rigorously, with appropriate controls, replication, and sample sizes. The conclusions must be drawn appropriately based on the data presented.

Reviewer #3: Partly

Reviewer #4: Yes

Reviewer #5: Partly

Reviewer #6: Yes

**********

3. Has the statistical analysis been performed appropriately and rigorously?

Reviewer #3: N/A

Reviewer #4: Yes

Reviewer #5: Yes

Reviewer #6: Yes

**********

4. Have the authors made all data underlying the findings in their manuscript fully available?

The PLOS Data policy requires authors to make all data underlying the findings described in their manuscript fully available without restriction, with rare exception (please refer to the Data Availability Statement in the manuscript PDF file). The data should be provided as part of the manuscript or its supporting information, or deposited to a public repository. For example, in addition to summary statistics, the data points behind means, medians and variance measures should be available. If there are restrictions on publicly sharing data—e.g. participant privacy or use of data from a third party—those must be specified.

Reviewer #3: Yes

Reviewer #4: No

Reviewer #5: No

Reviewer #6: No

**********

5. Is the manuscript presented in an intelligible fashion and written in standard English?

PLOS ONE does not copyedit accepted manuscripts, so the language in submitted articles must be clear, correct, and unambiguous. Any typographical or grammatical errors should be corrected at revision, so please note any specific errors here.

Reviewer #3: Yes

Reviewer #4: Yes

Reviewer #5: Yes

Reviewer #6: Yes

**********

6. Review Comments to the Author

Please use the space provided to explain your answers to the questions above. You may also include additional comments for the author, including concerns about dual publication, research ethics, or publication ethics. (Please upload your review as an attachment if it exceeds 20,000 characters)

Reviewer #3: Compared to the previous manuscript, the author has made adjustments based on the provided comments. However, the following issues remain:

1.Line 305: The reference is unidentifiable. Please verify and correct it accordingly.

2.As the author has stated, cognitive capital is a crucial component of social capital. However, the analysis of 983 valid questionnaires does not account for variations among participants across different occupations, age groups, and genders. Have these factors influenced the results? This aspect is particularly critical for the cognitive capital variable. The author appears to have provided only statistical data on educational background without conducting an associative analysis. Additionally, how do individuals from different occupations and age groups perceive public health environments and emergency healthcare? What are the key differences in their understanding?

3.The author has invited a statistical modeling expert to validate the accuracy of the manuscript, which is commendable. It is recommended to acknowledge this contribution in the Acknowledgment section to highlight the researcher's role in model validation.

4.Figures 1-3: The resolution is insufficient. Please provide high-resolution files for better clarity.

Reviewer #4: (No Response)

Reviewer #5: (No Response)

Reviewer #6: 1. Research Content and Innovation

This study investigates the impact of cultural appeal and social capital on the emergency response capacity of primary healthcare institutions and explores the differences between general communities and aging communities. The topic holds practical significance in the context of relatively weak emergency governance capacity at the grassroots level in China. The structural equation modeling (SEM) approach adopted in the study is well-established; however, the research’s innovation needs to be further emphasized. Are there existing studies on similar topics, and in what aspects does this study make breakthroughs? It is recommended to supplement the introduction with a review of relevant literature to highlight the study’s marginal contribution.

2. Research Methodology

In the sample selection section, the study selects four cities in Zhejiang Province for the survey and provides the GDP classification and sampling principles. However, further clarification is required:

① What is the rationale for selecting these four cities? Can they represent the entire Zhejiang Province or even the whole country? Is there any potential regional bias?

② What is the distribution of the sampled healthcare institutions? Does the sample cover different levels of primary healthcare institutions, such as community health service centers and township health centers?

③ Regarding sample randomness: Does the specific sampling method ensure that the survey results are broadly applicable?

In the variable measurement and model construction section:

① The study utilizes multiple dimensions of cultural appeal and social capital but does not clearly explain whether the measurement tools for these variables have been tested for reliability and validity. It is recommended to provide detailed measurement indicators in the methodology section and specify the source of the questionnaire.

② The study applies SEM for analysis, which is appropriate for exploring complex variable relationships. However, it does not provide sufficient information on model fit. It is suggested to include model fit indices to enhance the credibility of the results.

3. Research Results

① Descriptive statistics: The study should provide a more comprehensive description of the sample characteristics, including respondents' age, occupational distribution, and educational background, to enhance research transparency.

② Multi-group SEM analysis: The study mentions that multi-group SEM analysis was conducted between general and aging communities, revealing that cultural appeal has a more significant direct effect in aging communities. However, was a significance test performed? If not, it is recommended to conduct additional significance tests to strengthen the robustness of the conclusions.

4. Conclusion and Discussion

The study should further integrate its findings to propose more actionable policy recommendations on improving the emergency response capacity of primary healthcare institutions. For example, how can community governance be leveraged to optimize social capital? How can the cultural appeal of aging communities be enhanced to improve healthcare response capacity?

5. Language and Formatting

The language of the paper is generally clear; however, some expressions are redundant and should be further refined. For instance, the abstract contains repetitive information that could be condensed to better highlight the research findings. Additionally, the reference format must strictly comply with the journal’s requirements, and citation formatting should be carefully checked for accuracy.

**********

7. PLOS authors have the option to publish the peer review history of their article (what does this mean?). If published, this will include your full peer review and any attached files.

If you choose “no”, your identity will remain anonymous but your review may still be made public.

**Do you want your identity to be public for this peer review?**  For information about this choice, including consent withdrawal, please see our Privacy Policy.

Reviewer #3: No

Reviewer #4: No

Reviewer #5: No

Reviewer #6: No

**********

[NOTE: If reviewer comments were submitted as an attachment file, they will be attached to this email and accessible via the submission site. Please log into your account, locate the manuscript record, and check for the action link "View Attachments". If this link does not appear, there are no attachment files.]

While revising your submission, please upload your figure files to the Preflight Analysis and Conversion Engine (PACE) digital diagnostic tool, https://pacev2.apexcovantage.com/. PACE helps ensure that figures meet PLOS requirements. To use PACE, you must first register as a user. Registration is free. Then, login and navigate to the UPLOAD tab, where you will find detailed instructions on how to use the tool. If you encounter any issues or have any questions when using PACE, please email PLOS at figures@plos.org. Please note that Supporting Information files do not need this step.

# Author comment: Author response to Decision Letter 2

**DOI:** 10.1371/journal.pone.0351875.r004

17 May 2025

May 17, 2025

Chunyu Zhang

Academic Editor

PLOS ONE

RE: “How do cultural appeal and social capital differ in influencing primary healthcare emergency capabilities in ordinary versus older communities?” Manuscript ID: PONE-D-24-36223

Dear Editor:

Thank you for the opportunity to further revise our manuscript “How do cultural appeal and social capital differ in influencing primary healthcare emergency capabilities in ordinary versus older communities?” The reviewers’ and editors’ comments were useful and helped to clarify and improve several aspects of the manuscript.

Please find attached the revised version of the manuscript; the changes made are indicated using red font. Below is a point-by-point response to the issues raised by the reviewers and editors.

In addition, we would like to inform you of an update regarding the authors’ institutional affiliations. Due to a recent organizational restructuring at Hangzhou Normal University, the Department of Health Policy and Management, formerly part of the School of Public Health, was officially merged with the School of Public Administration on March 2, 2025. Accordingly, the affiliations of five authors —Xianhong Huang, Jiamin Tang, Jie Jia, Kaidi Sun, and ZhengNan Meng — should be updated from the School of Public Health, Hangzhou Normal University to the School of Public Administration, Hangzhou Normal University. We respectfully submit this request for affiliation correction. Should any additional documentation or clarification be required, we are happy to provide it promptly.

We hope that this version is suitable for publication in PLOS ONE. We look forward to hearing from you.

Xiaoting Zhang

School of Public Administration, Hangzhou Normal University

Hangzhou 311121, China

zxtbing@hznu.edu.cn

Point-by-point response.

Editorial

1.What is the practical contribution of the research?

Response: Thank you for highlighting the need to clarify our study’s practical contributions. We have revised the manuscript to address this, particularly in the “Study implications and limitations” section. Our findings offer actionable contributions. These findings offer practical guidance for incorporating cultural appeal into the daily management practices of both communities and primary healthcare institutions, and further inform the development of targeted strategies tailored to different community types.

[Study implications and limitations] (page 34, lines 721–733): Regarding its practical contributions, this study empirically demonstrates the importance of cultural appeal, social capital, and healthcare emergency capacity, offering evidence to support the enhancement of emergency preparedness in primary healthcare institutions. Communities and grassroots medical institutions can integrate organizational culture training, value advocacy, and daily management to foster social capital and improve healthcare emergency capacity. An innovative aspect of this research is its use of multi-group analysis, which confirms the varying effects of cultural appeal and social capital on healthcare emergency capacity across various community types. This provides a solid foundation for developing tailored prevention and mobilization strategies. Policymakers at all levels can use these insights to design community-specific initiatives for cultural promotion and social capital development to improve the emergency resilience of primary healthcare institutions in diverse contexts.

2. The first chapter has too many paragraphs and the logic is confused.

Response: Thank you for your critical feedback on the logical structure of the Introduction. We have thoroughly restructured this section to enhance clarity and flow, as detailed below and in the revised manuscript (see revised manuscript, Introduction, pages 4–6, lines 81–84, lines 101–103, lines 111–120, lines 125–134).

First, the opening paragraph outlines the significance and current state of primary healthcare institutions and their emergency response capacity. It also highlights the challenges these institutions have faced during public health emergencies, which expose critical gaps in their emergency preparedness.

Second, we reviewed the existing literature to establish the theoretical foundation and practical relevance of the two core variables in our study—cultural appeal and social capital. We note that previous research has predominantly focused on managerial aspects such as resource allocation and professional training for medical staff, while comparatively less attention has been given to “awareness-level” factors, particularly those related to cultural dimensions. At the same time, there is growing scholarly interest in the role of social networks—such as participation, interaction, and trust—in promoting resource integration and information sharing. In this context, social capital theory provides a valuable perspective for understanding emergency response within grassroots healthcare settings.

Third, the literature review reveals a lack of comparative studies examining differences in emergency response capacity across various community types. To address this gap, our study incorporates the community type dimension and adopts a multi-group analysis approach.

Finally, we concluded with the study’s objectives, directly tied to the preceding gaps.

These revisions ensure a logical progression from problem identification → theoretical grounding → gap articulation → objective formulation, addressing the reviewer’s concerns about disjointed logic. We appreciate the opportunity to strengthen the manuscript’s rigor."

3. All hypotheses need to be rewritten. The current research hypothesis reads less like an academic description than a news report.

Response: Thank you for your valuable comments. Your suggestions provided clear direction for improving the overall quality of our research. We have thoroughly revised all research hypotheses to enhance academic rigor and logical coherence. In particular, we have eliminated colloquial expressions and avoided language resembling journalistic reporting, ensuring that the phrasing aligns to the conventions of scholarly writing (see revised manuscript, Literature review and hypotheses, pages 6–11, lines 137–247).

4.The discussion section needs to distinguish between academic and practical.

Response: Thank you for your insightful comments. In the revised Discussion section, we have provided an academically rigorous analysis of the relationships among cultural appeal, social capital, and healthcare emergency capacity. To support real-world application, we conclude each subsection with practical and actionable recommendations (see revised manuscript, pages 28–34, lines 565–571, lines 601–611, lines 621–629, lines 635–641, lines 667–708).

Furthermore, we have divided the significance of this study into two clearly defined components: theoretical implications and practical implications. This structural refinement is intended to help readers clearly distinguish the academic contributions from the practical relevance of the research.

[Study implications and limitations] (page 34, lines 709–732): “This study demonstrates the significant influence of cultural appeal and social capital on healthcare emergency preparedness, offering both theoretical and practical contributions. From a theoretical perspective, the study introduces the concept of cultural appeal into research on healthcare emergency capacity in grassroots medical institutions, addressing a gap in existing emergency management research that has predominantly focused on systems, resource allocation, or technical capabilities. It highlights the central role of cultural mobilization in emergency response. Furthermore, drawing on social capital theory, the study examines the relationships between cultural appeal, three types of social capital, and healthcare emergency capacity, thereby extending the application of social capital theory within the field of healthcare emergency management.

Regarding its practical contributions, this study empirically demonstrates the importance of cultural appeal, social capital, and healthcare emergency capacity, offering evidence to support the enhancement of emergency preparedness in primary healthcare institutions. Communities and grassroots medical institutions can integrate organizational culture training, value advocacy, and daily management to foster social capital and improve healthcare emergency capacity. An innovative aspect of this research is its use of multi-group analysis, which confirms the varying effects of cultural appeal and social capital on healthcare emergency capacity across various community types. This provides a solid foundation for developing tailored prevention and mobilization strategies. Policymakers at all levels can use these insights to design community-specific initiatives for cultural promotion and social capital development to improve the emergency resilience of primary healthcare institutions in diverse contexts.”

5. The references do not meet journal standards.

Response: Thank you for your valuable suggestion. We have conducted a thorough review and standardization of all references throughout the manuscript. Both the in-text citations and reference list entries have been revised to fully comply with the journal’s formatting requirements. We apologize for this oversight and thank you for your patience.

Reviewer #3

Compared to the previous manuscript, the author has made adjustments based on the provided comments. However, the following issues remain:

1.Line 305: The reference is unidentifiable. Please verify and correct it accordingly.

Response: Thank you for your thorough review. The reference cited in line 305 is from a Chinese-language journal article. In accordance with the journal’s formatting requirements, we have now included the publication year, volume, issue number, and page range to enhance its clarity and traceability.

2.As the author has stated, cognitive capital is a crucial component of social capital. However, the analysis of 983 valid questionnaires does not account for variations among participants across different occupations, age groups, and genders. Have these factors influenced the results? This aspect is particularly critical for the cognitive capital variable. The author appears to have provided only statistical data on educational background without conducting an associative analysis. Additionally, how do individuals from different occupations and age groups perceive public health environments and emergency healthcare? what are the key differences in their understanding?

Response: Thank you for your valuable comments. We fully acknowledge the potential influence of individual differences on the study’s outcomes. In the current study, we conducted preliminary examinations of key demographic variables—namely gender, age, and occupation—through descriptive statistics and correlation analyses. Additionally, we performed univariate analyses using public health emergency capability as the dependent variable, with gender, age, and occupation as independent variables. The results indicated a statistically significant difference for occupation (P < 0.05), while no significant differences were found for gender or age (P > 0.05). These results have been added to Supplementary Table 1 and are now detailed in the Methods section (Lines 309–319) of the revised manuscript.

Second, we appreciate your insightful observation regarding the potential influence of occupation, age, and gender, particularly in relation to cognitive social capital. We would like to clarify that in this study, cognitive social capital functions as a mediating variable within the proposed theoretical model. The central focus of our research is to examine differences between ordinary and older communities and to explore how cultural appeal influences the public health emergency capacity of primary healthcare institutions through social capital. Therefore, we did not include demographic variables such as occupation, age, and gender as primary explanatory or moderating variables in our model to assess their effects on cognitive social capital.

we conducted an analysis using cognitive social capital as the dependent variable and gender, age, occupation, and community type as independent variables. The results revealed significant differences across occupation and community type (p < 0.05). In addition to univariate tests, we conducted multivariate regression analyses to isolate the effects of occupation and community type on cognitive social capital while controlling for community type. Occupation remained significant, explaining 1.2% of the variance in cognitive capital.

Although these findings offer promising directions for future research, incorporating them into the main model would substantially expand the scope of this study. As such, we have not retained these preliminary results in the main manuscript. Instead, we have included a discussion of this point in the “Study implications and limitations” section (Lines 740–747), where we suggest that future studies may consider using interaction terms or multi-group SEM approaches to explore group heterogeneity in health emergency capacity and social capital within primary healthcare institutions.

Finally, in response to your question regarding how individuals of different occupations and age groups perceive public health environments and emergency medical services—and what key differences exist in their understanding—we focused primarily on occupational differences, given that occupation was the only demographic variable that showed statistical significance in our analysis. Based on a review of relevant literature, we offer the following preliminary interpretation: physicians may prioritize the coordination of medical resources and clinical response efficiency; nurses are more likely to focus on frontline workflows and patient communication; medical technicians tend to emphasize the availability and operational efficiency of diagnostic support systems; and administrative staff typically concentrate on emergency planning, policy implementation, and inter-organizational coordination. These differing perspectives are closely aligned with the distinct roles of occupational group within the public health emergency response system of primary healthcare institutions. We believe these insights provide a valuable foundation for future in-depth studies, which we plan to pursue in subsequent research.

[Methods] (page 14, lines 311–321): “Over half the participants were under the age of 40, and three-quarters held an associate degree or higher. The primary professional groups represented were physicians (41.1%) and nurses (32.0%). Approximately 75% of the respondents held a professional title, and 72.5% had more than six years of work experience. Regarding institutional context, 72.6% of the respondents were employed at primary healthcare institutions located in ordinary communities, while 27.4% worked in older communities. Statistical analyses revealed significant differences in the public health emergency response capacity of primary healthcare institutions based on profession, community type, presence of hazard identification and risk assessment systems, implementation of a public health emergency accountability system, and perceived usefulness of intelligent tools. (Supplementary Table 1).”

[Study implications and limitations] (page 36, lines 740–747): “Finally, although this study employs a multi-group structural equation modeling approach to analyze the impact of different community types on health emergency capacity, it does not examine the role of factors such as healthcare professionals' gender or age. Future research could consider incorporating interaction terms or conducting multi-group structural model analyses based on factors such as age, gender, and profession to further explore the differences in health emergency capacity among various groups, thereby providing a more comprehensive understanding.”

3.The author has invited a statistical modeling expert to validate the accuracy of the manuscript, which is commendable. It is recommended to acknowledge this contribution in the Acknowledgment section to highlight the r

# Peer review: Decision Letter 2

**DOI:** 10.1371/journal.pone.0351875.r005

**Authors:** Chunyu Zhang

22 Jul 2025

<div>PONE-D-24-36223R2

How do cultural appeal and social capital differ in influencing primary health care emergency capabilities in ordinary versus older communities?

PLOS ONE

Dear Dr. Zhang,

Thank you for submitting your manuscript to PLOS ONE. After careful consideration, we feel that it has merit but does not fully meet PLOS ONE’s publication criteria as it currently stands. Therefore, we invite you to submit a revised version of the manuscript that addresses the points raised during the review process.

I'm very sorry that I have to find new reviewers because the previous one was unwilling to continue reviewing this manuscript. Two reviewers raised doubts about the references. PLOS ONE is an international journal. All the references of the manuscripts must be retrievable; otherwise, I have reason to suspect that the content is generated by AI. Therefore, I suggest that the author cite famous English database literature.

Please submit your revised manuscript by Sep 05 2025 11:59PM. If you will need more time than this to complete your revisions, please reply to this message or contact the journal office at plosone@plos.org. When you're ready to submit your revision, log on to https://www.editorialmanager.com/pone/ and select the 'Submissions Needing Revision' folder to locate your manuscript file.

Please include the following items when submitting your revised manuscript:

- A rebuttal letter that responds to each point raised by the academic editor and reviewer(s). You should upload this letter as a separate file labeled 'Response to Reviewers'.
- A marked-up copy of your manuscript that highlights changes made to the original version. You should upload this as a separate file labeled 'Revised Manuscript with Track Changes'.
- An unmarked version of your revised paper without tracked changes. You should upload this as a separate file labeled 'Manuscript'.

If you would like to make changes to your financial disclosure, please include your updated statement in your cover letter. Guidelines for resubmitting your figure files are available below the reviewer comments at the end of this letter.

If applicable, we recommend that you deposit your laboratory protocols in protocols.io to enhance the reproducibility of your results. Protocols.io assigns your protocol its own identifier (DOI) so that it can be cited independently in the future. For instructions see: https://journals.plos.org/plosone/s/submission-guidelines#loc-laboratory-protocols. Additionally, PLOS ONE offers an option for publishing peer-reviewed Lab Protocol articles, which describe protocols hosted on protocols.io. Read more information on sharing protocols at https://plos.org/protocols?utm_medium=editorial-email&utm_source=authorletters&utm_campaign=protocols.

We look forward to receiving your revised manuscript.

Kind regards,

Chunyu Zhang

Academic Editor

PLOS ONE

Journal Requirements:

If the reviewer comments include a recommendation to cite specific previously published works, please review and evaluate these publications to determine whether they are relevant and should be cited. There is no requirement to cite these works unless the editor has indicated otherwise.

[Note: HTML markup is below. Please do not edit.]

Reviewers' comments:

Reviewer's Responses to Questions

**Comments to the Author**

1. If the authors have adequately addressed your comments raised in a previous round of review and you feel that this manuscript is now acceptable for publication, you may indicate that here to bypass the “Comments to the Author” section, enter your conflict of interest statement in the “Confidential to Editor” section, and submit your "Accept" recommendation.

Reviewer #7: (No Response)

Reviewer #8: All comments have been addressed

**********

2. Is the manuscript technically sound, and do the data support the conclusions?

The manuscript must describe a technically sound piece of scientific research with data that supports the conclusions. Experiments must have been conducted rigorously, with appropriate controls, replication, and sample sizes. The conclusions must be drawn appropriately based on the data presented.

Reviewer #7: Yes

Reviewer #8: Yes

**********

3. Has the statistical analysis been performed appropriately and rigorously?

Reviewer #7: Yes

Reviewer #8: Yes

**********

4. Have the authors made all data underlying the findings in their manuscript fully available?

The PLOS Data policy requires authors to make all data underlying the findings described in their manuscript fully available without restriction, with rare exception (please refer to the Data Availability Statement in the manuscript PDF file). The data should be provided as part of the manuscript or its supporting information, or deposited to a public repository. For example, in addition to summary statistics, the data points behind means, medians and variance measures should be available. If there are restrictions on publicly sharing data—e.g. participant privacy or use of data from a third party—those must be specified.

Reviewer #7: Yes

Reviewer #8: Yes

**********

5. Is the manuscript presented in an intelligible fashion and written in standard English?

PLOS ONE does not copyedit accepted manuscripts, so the language in submitted articles must be clear, correct, and unambiguous. Any typographical or grammatical errors should be corrected at revision, so please note any specific errors here.

Reviewer #7: Yes

Reviewer #8: No

**********

6. Review Comments to the Author

Please use the space provided to explain your answers to the questions above. You may also include additional comments for the author, including concerns about dual publication, research ethics, or publication ethics. (Please upload your review as an attachment if it exceeds 20,000 characters)

Reviewer #7: The research topic is relevant to scientists and public health organisations. Emergency response capacity in primary health care is essential for the resilience of health systems and for the safety of populations in critical contexts.

However, I will identify the areas for improvement below.

1. Methodological limitations and representativeness.

The article is based on a sample of four cities in Zhejiang Province, China, one of the most developed in China. This limits the generalisation of the results to less favourable regions or other countries. Therefore, these factors should be considered in the limitations of the study.

2. The impact of individual variables such as age and gender is not measured, focusing only on profession. The literature indicates that these dimensions can affect perceptions about social capital and response capacity. It is suggested that these variables could be part of the suggestions for future research.

3. Measurements and validation of instruments. The authors should provide more explicit sources for the scales since they are not obtained from the entire international literature. The authors should explain the translation processes and the rationale behind their selection.

4. Discussion of innovation and marginal contributions.

The authors should refer to recent international literature on social capital, cultural appeal and emergency management, to specify the advances of this study in relation to existing approaches.

5. Argumentative redundancy and structuring.

I suggest simplifying the text in the discussion of results and conclusions. The authors should rewrite these sections by prioritising a structure that includes contributions to theory, management of organisations, limitations, and suggestions for future research. Currently, the text exhibits a degree of repetition, leading the reader to become disoriented among numerous sections.

6. Inconsistency and updating of literature references

The authors should ensure that the references are available internationally. Some references are difficult to identify, particularly from articles in Chinese without translation or complete data. I recommend incorporating more recent and relevant international literature.

Reviewer #8: The authors have provided detailed and appropriate responses to the previous reviewers' comments and have made corresponding revisions to the manuscript accordingly. However, the following issues remain:

1.The reference list requires a thorough and meticulous revision to ensure it fully complies with the journal's formatting guidelines. There are systemic inconsistencies and errors in punctuation and author names. Some Chinese references are not searchable, please attach the correct reference links. This lack of attention to detail detracts from the scholarly quality of the manuscript.

2. The core independent variable, "cultural appeal," remains conceptually ambiguous and lacks a solid theoretical foundation in organizational studies. Combined with previous modified versions, the measurement of this concept is based on what appears to be a fabricated or non-existent source. It is necessary to further demonstrate and elaborate on the connotation of cultural appeal.

**********

7. PLOS authors have the option to publish the peer review history of their article (what does this mean?). If published, this will include your full peer review and any attached files.

If you choose “no”, your identity will remain anonymous but your review may still be made public.

**Do you want your identity to be public for this peer review?**  For information about this choice, including consent withdrawal, please see our Privacy Policy.

Reviewer #7: No

Reviewer #8: No

**********

[NOTE: If reviewer comments were submitted as an attachment file, they will be attached to this email and accessible via the submission site. Please log into your account, locate the manuscript record, and check for the action link "View Attachments". If this link does not appear, there are no attachment files.]

While revising your submission, please upload your figure files to the Preflight Analysis and Conversion Engine (PACE) digital diagnostic tool, https://pacev2.apexcovantage.com/. PACE helps ensure that figures meet PLOS requirements. To use PACE, you must first register as a user. Registration is free. Then, login and navigate to the UPLOAD tab, where you will find detailed instructions on how to use the tool. If you encounter any issues or have any questions when using PACE, please email PLOS at figures@plos.org. Please note that Supporting Information files do not need this step.

# Author comment: Author response to Decision Letter 3

**DOI:** 10.1371/journal.pone.0351875.r006

23 Aug 2025

Point-by-point responses to the Reviewers’ comments

Reviewer #7

The research topic is relevant to scientists and public health organisations. Emergency response capacity in primary health care is essential for the resilience of health systems and for the safety of populations in critical contexts.

1. Methodological limitations and representativeness

The article is based on a sample of four cities in Zhejiang province, China, one of the most developed in China. This limits the generalisation of the results to less favourable regions or other countries, Therefore, these factors should be considered in the limitations of the study.

Response：Thank you for raising this issue. In response to your concern about geographical sampling bias¬¬—specifically, the concentration of samples in relatively developed areas of Zhejiang Province—we have revised the “Research Limitations and Prospects” section to address this issue, as follows:

（1） We explicitly acknowledge the geographical limitation of our sample—namely, its concentration in the developed eastern region of China—and note that this socioeconomic context represents a key methodological constraint.

（2） We clearly state that, due to the absence of samples from less-developed regions, our findings may not be directly generalizable to underdeveloped areas such as central and western China.

（3）We recommend that future research incorporate samples from regions across different levels of development in China and include cross-regional comparative analyses to explore the potential influence of socioeconomic differences on the study’s conclusions. We also suggest conducting cross-cultural comparative studies with samples from similar research contexts internationally to validate the robustness and generalizability of the findings.

For details of the specific revisions, please see the revised manuscript [Research limitations and prospects] (pages 35-36, lines 715–736).

2. The impact of individual variables such as age and gender is not measured, focusing only on profession.

The literature indicates that these dimensions can affect perceptions about social capital and response capacity. It is suggested that these variables could be part of the suggestions for future research.

Response：We thank the reviewer for their insightful comments and for highlighting an important methodological limitation in our study’s design. As noted, demographic factors—such as gender and age—may act as potential confounders in estimating the relationships between occupation, social capital, and healthcare emergency preparedness, which could affect the internal validity and inferential strength of our findings.

To address this methodological limitation more thoroughly, we have added a discussion in the [Research Limitations and Prospects, lines 715–736] section regarding the limitations of the demographic variables (e.g., occupation, age, gender). Specifically, we acknowledge that future research should consider gender, age, and similar variables either as primary explanatory variables or as moderating factors, and utilize interaction models or multi-group structural equation modeling to examine their potential impact on the relationship between social capital and healthcare emergency preparedness.

Although our preliminary analyses indicated that demographic characteristics such as occupation and community type significantly influenced emergency preparedness, gender and age did not reach statistical significance, this does not eliminate the possibility that these factors could influence outcomes under different societal or cultural contexts (Table1). Accordingly, we recommend that future studies validate these demographic influences across diverse regions and populations. Moreover, using more precise model specifications to estimate the independent effects of occupation and other demographic factors can help control for potential confounding, thereby enhancing both the internal and external validity—and generalizability—of the results.

Table 1. Univariate Analysis of Health Emergency Capacity(The table is only used to reply to the reviewer 's suggestion, not as the text or annex content.)

Variable Categorization Frequency (N) Composition（%） t/F P

Gender Male 216 22 2.241 0.349

Female 767 78

Age 18-30 years 304 30.9 0.721 0.577

31-40 years 369 37.5

18-30 years 225 22.9

42-50 years 73 7.4

51-60 years 12 1.2

61 years and above 304 30.9

Educational level Junior high school or below 17 1.7 0.527 0.716

High school 47 4.8

Associate degree 251 25.5

Bachelor’s degree or above 668 68

Marital status Single 181 18.4 0.050 0.985

Married 784 79.8

Divorced and other 18 1.8

Position Doctor 404 41.1 3.111 0.009

Nurse 315 32

Medical technician 102 10.4

Prevention 88 9

Administration 30 3.1

Other 44 4.5

Professional title None 125 12.7 0.165 0.920

Junior 484 49.2

Intermediate 286 29.1

Senior 88 9

Years of service Less than 1 year 28 2.8 0.540 0.655

1-3 years 117 11.9

4-6 years 125 12.7

More than 6 years 713 72.5

The type of community you work in Ordinary community 714 72.6 0.789 0.013

old community 269 27.4

The presence of hazard identification and risk assessment management systems Yes 788 80.2 24.334 <0.001

No 74 7.5

Don't know 121 12.3

Accountability systems for public health emergencies Yes 865 88 30.239 <0.001

No 47 4.8

Don't know 71 7.2

The extent of the usefulness of smart tools very helpful 763 77.6 43787 <0.001

Helpful 199 20.2

Average 19 1.9

3. Measurements and validation of instruments

The authors should provide more explicit sources for the scales since they are not obtained from the entire international literature. The authors should explain the translation processes and the rationale behind their selection.

Response：Thank you for your suggestion. It was correctly noted that the primary measurement instrument for cultural cohesion—or “cultural appeal”—used in our study is based on Chinese-language literature. We provide the following clarifications:

The measurement scale for cultural cohesion was principally adapted from the theoretical framework and definitions presented by Chen Li in the Chinese core journal Journalism Lover. Our research team then contextualized and refined these items to fit the realities of primary healthcare emergency responses in China xwahz.com.

It should be noted that there is presently no internationally established scale explicitly measuring “cultural cohesion of Chinese grassroots healthcare organizations during emergency conditions.” Thus, Chen Li’s work offers essential theoretical grounding and strong contextual relevance for constructing a culturally appropriate instrument.

Accordingly, we localized and streamlined the scale to align closely with our study context, ensuring the measurement items are highly relevant and accurate for our research setting. Since Chen Li’s study is available only in Chinese and is not indexed in major international databases, we will include the full Chinese PDF version along with an English translation of the key content in the revised submission for the reviewer’s convenience. The questionnaire was administered in Chinese to the local sample.

Reliability and validity metrics for our adapted instrument in our sample are strong: Cronbach’s α = 0.969, Composite Reliability (CR) = 0.970, and Average Variance Extracted (AVE) = 0.915, which attest to the measure’s excellent internal consistency and construct validity.

To ensure conceptual clarity and semantic equivalence in English, we followed a standard cross-cultural translation procedure: two bilingual experts with public health management backgrounds independently translated the Chinese questionnaire into English; discrepancies were resolved through discussion between the translators and the lead researcher to produce a unified English draft; a third bilingual expert then back-translated this into Chinese, and item-by-item comparisons with the original confirmed high conceptual equivalence between Chinese and English versions.

The Chinese version and English translation of Chen Li’s study are selected:

国际传播能力建设的“五力”，“确定的是新时代国际传播能力建设的效能指标，隐含着全媒体传播、文化传播、形象传播、知识传播与舆论引导五个方面的内容，涵盖了传统意义上国际传播的各个领域，展现了对国际传播效能全方位提升的总体要求”[3]。“五力”建设是一项系统工程，“五力”互为表里，相互促进。经过近几年的努力，我们在国际传播能力建设上已经初具规模，但国际传播能力在内涵上还需进一步深化。“传播中华文化，讲好中国故事”是国际传播的核心内涵和重要内容。意大利共产党创始人之一的葛兰西曾提出“文化领导权”理论，认为优秀文化在多元文化竞争中会居于主导地位，自然就会获得“知识”和“道德领导权”。而在约瑟夫·奈那里，文化是一个国家最为重要的“软实力”，软实力靠的是自身的吸引力，而不是强迫别人去信服。[4]中华文化吸引力就是中华文化的吸引力、感染力、影响力、引领力，它既是国际传播能力建设的核心构成，是国际传播能力建设的灵魂和基础，也是最能表达和突出中国特色的一部分。因此，提升中华文化吸引力是我国国际传播能力建设的关键和重要内容，也是提高国际传播效能的重要内容。我们在文化自信的基础上，不断提升中华文化感召力，在文化的内涵挖掘、表达方式创新、传播方式提升等方面不断努力，将会显著推进我国国际传播能力的转型升级，增强中华文化的全球影响力，彰显中华文化的深刻内涵、独特魅力和优秀品质，进而取得良好的国际传播效果。

二、中华文化感召力的思想内涵与特征

（一）中华文化感召力的思想内涵

何谓文化？我国文化学家梁漱溟先生给文化下了一个无所不包的定义：文化，就是吾人生活所依靠之一切……盖人生诸于世界，无非做三件事：活着、动（工作）着、觉（思考并言说或者书写）着。人做这三件事又必须依靠三种方式：生活方式、行为方式、思维方式。人因三件事和一种方式而生活起来就是文化。[5]所以中华文化就是几千年来的文明进程中，中国人形成的生活方式、行为方式和思维方式。英国文化学者雷蒙德·威廉斯在《文化与社会》中提出，文化即“全部的生活方式，包括物质的、知识的和精神的”[6]。而马克思认为文化是“自然的人化”。文化是人类改造世界中一种巨大力量，可以称之为“文化力”或“文化生产力”。[7]因此可以说，文化感召力就是文化的一种内在表现。

中华文化感召力是文化软实力社会生活的本质，要具有吸引力、召唤力、感染力、凝聚力，能够教育人、鼓舞人、激励人、凝聚人、团结人。[8]文化感召力是文化软实力的一个重要体现，文化软实力的核心是文化所具有的感召力，正如孔子所认为的“近者悦，远者来”，中华文化感召力是中国人的生活方式、思维方式和行为方式所具有的感染力、号召力、引导力。

Translated version:

The "Five Powers" framework for international communication capacity building establishes performance indicators for the new era, encompassing five dimensions: omni-media communication, cultural dissemination, image cultivation, knowledge sharing, and public opinion guidance. It covers all aspects of traditional international communication and cultural exchanges, presenting comprehensive requirements for holistic improvement in communication effectiveness [2]. As a systematic project, the Five Powers complement each other and reinforce one another. While initial progress has been made in recent years, deeper development is needed in the substantive aspects of international communication capabilities. "Promoting China's outstanding culture and showcasing its evolving development" form the core essence of international communication. As Gramsci, co-founder of the Italian Communist Party, proposed the theory of "cultural hegemony," where superior cultures naturally gain "knowledge and moral leadership" in multicultural competition [3]. Joseph Nye further emphasized that culture constitutes a nation's most vital "soft power"—a strength derived from intrinsic appeal rather than coercion [4]. The cultural magnetism of Chinese heritage—its attractiveness, influence, and guiding role—serves as the cornerstone of international communication capacity building. This element embodies Chinese characteristics most vividly. Therefore, enhancing this cultural magnetism is both the key focus and essential component of international communication capacity development, significantly improving global communication effectiveness. Building on cultural confidence, we are continuously enhancing the appeal of Chinese culture through in-depth exploration of its essence, innovative approaches to expression, and improved dissemination effectiveness. These efforts will significantly advance the transformation and upgrading of China's international communication capabilities, amplify the global influence of Chinese culture, and highlight its profound depth, unique charm, and outstanding qualities—ultimately achieving remarkable international communication outcomes.

The Ideological Content and Characteristics of the Appeal to Chinese Culture

(1) The Ideological Connotation of the Appeal of Chinese Culture

What is culture? Chinese cultural scholar Liang Shuming provided an all-encompassing definition: Culture is everything we rely on for living... As humans exist in the world, we essentially engage in three fundamental activities: living, working, and thinking (expressing thoughts through speech or writing). To perform these activities, we must adopt three modes of existence: lifestyle, behavior, and thought. The combination of these three aspects and their corresponding modes constitutes culture. [5] Thus, Chinese culture represents the lifestyle, behavioral patterns, and cognitive frameworks developed by the Chinese people over millennia of civilizational development. British cultural scholar Raymond W. Kwan proposed in "Culture and Society" that culture encompasses "the totality of life—material, intellectual, and spiritual" [6]. Marx viewed culture as "the humanization of nature." Culture serves as humanity's immense power to transform the world, often termed "cultural force" or "cultural productivity." [7] Therefore, cultural appeal can be seen as an inherent manifestation of this cultural force.

The cultural appeal of Chinese civilization embodies the essence of culture reflecting real social life. It should possess attractiveness, appeal, influence, and cohesion, capable of educating, inspiring, motivating, urging, and uniting people. [8] Cultural appeal serves as a crucial manifestation of cultural soft power, whose core lies in the inherent appeal of culture. As Confucius observed, "Those nearby are pleased, while those afar come." The cultural appeal of Chinese civilization refers to the infectiousness, rallying power, and guiding influence inherent in the lifestyle, thinking patterns, and behavioral norms of the Chinese people.

4. Discussion of innovation and marginal contributions

The authors should refer to recent international literature on social capital, cultural appeal and emergency management, to specify the advances of this study in relation to existing approaches.

Response：We reviewed recent international literature and, based on both the literature review and a comparative analysis, supplemented the Conclusion and Theoretical Implications sections to emphasize the threefold innovation of this study’s research perspective and methodology:

(1) Regarding the research focus, we selected primary healthcare institutions as the core analytic unit, unlike most international studies that concentrate on large hospitals or macro-level emergency systems. Primary healthcare institutions act as the “nerve endings” of the health emergency system and play a direct role in community-level crisis response. However, the international literature places limited emphasis on this domain. Our study elucidates the mechanisms shaping emergency preparedness in grassroots settings, thereby offering empirical, context-specific contributions to the global discourse on health emergency management.

(2) In terms of theoretical variable integration, this study systematically incorporates cultural appeal as a key antecedent variable within the theoretical framework of emergency preparedness. This extends beyond existing models, which largely hinge on structural or cognitive social capital and technological or resource-based factors. Our empirical analysis confirms a positive effect of cultural appeal on grassroots emergency preparedness, thereby offering a theoretical anchor for understanding the critical influence of informal institutions and cultural soft power in crisis response.

(3) On methodology and contextualized comparison, we innovatively employ multi-group structural equation modeling based on community types—specifically, old communities versus ordinary community—to systematically test for contextual differences in causal paths. This method reveals both universal and unique mechanisms driving emergency capability and provides concrete process evidence aligning with Shi et al., who propose that “resilience of healthcare organizations is influenced more broadly by community and healthcare systems.” Our findings clarify how community type shapes the influence and relative importance of key driving factors.

The source referenced is [Shi W, Chen R, Wang K, Wang Y, Gui L. Exploring hospital resilience protective or risk factors: lessons for future disaster response efforts. Front Public Health. 2024 Mar 27;12:1378257. doi: 10.3389/fpubh.2024.1378257.]

For details of the specific revisions, please see the revised manuscript [Conclusion] (pages 32-33, lines 642–662), [Theoretical contribution] (pages 33-34, lines 670–680) .

5. Argumentative redundancy and structuring

I suggest simplifying the text in t

Attachment Submitted filename: Response to Reviewers(8.23).docx

# Peer review: Decision Letter 3

**DOI:** 10.1371/journal.pone.0351875.r007

**Authors:** Chunyu Zhang

12 Oct 2025

PONE-D-24-36223R3

How do cultural appeal and social capital differ in influencing primary health care emergency capabilities in ordinary versus older communities?

PLOS ONE

Dear Dr. Zhang,

Thank you for submitting your manuscript to PLOS ONE. After careful consideration, we feel that it has merit but does not fully meet PLOS ONE’s publication criteria as it currently stands. Therefore, we invite you to submit a revised version of the manuscript that addresses the points raised during the review process.

While the manuscript addresses an important topic concerning social capital and emergency capacities in primary healthcare institutions, it still falls short of *PLOS ONE* ’s publication standards in several aspects. In particular, (1) **the presence of Chinese-language content**  in both the response letter and supplementary sections is unacceptable for an international English journal, and (2) **the inclusion of Chinese-language sources translated into English**  does not meet the requirement for internationally retrievable references. In addition, the theoretical construct of *“cultural appeal”*  remains conceptually vague and lacks a clear grounding in established international literature. The manuscript would benefit from reframing this construct within recognized theories of organizational culture or cultural competence, replacing all non-English citations with recent international studies, and ensuring full linguistic and stylistic consistency across the text. A thorough language revision by a professional English editor is strongly recommended before resubmission.

Please submit your revised manuscript by Nov 26 2025 11:59PM. If you will need more time than this to complete your revisions, please reply to this message or contact the journal office at plosone@plos.org. When you're ready to submit your revision, log on to https://www.editorialmanager.com/pone/ and select the 'Submissions Needing Revision' folder to locate your manuscript file.

Please include the following items when submitting your revised manuscript:

- A rebuttal letter that responds to each point raised by the academic editor and reviewer(s). You should upload this letter as a separate file labeled 'Response to Reviewers'.
- A marked-up copy of your manuscript that highlights changes made to the original version. You should upload this as a separate file labeled 'Revised Manuscript with Track Changes'.
- An unmarked version of your revised paper without tracked changes. You should upload this as a separate file labeled 'Manuscript'.

If you would like to make changes to your financial disclosure, please include your updated statement in your cover letter. Guidelines for resubmitting your figure files are available below the reviewer comments at the end of this letter.

If applicable, we recommend that you deposit your laboratory protocols in protocols.io to enhance the reproducibility of your results. Protocols.io assigns your protocol its own identifier (DOI) so that it can be cited independently in the future. For instructions see: https://journals.plos.org/plosone/s/submission-guidelines#loc-laboratory-protocols. Additionally, PLOS ONE offers an option for publishing peer-reviewed Lab Protocol articles, which describe protocols hosted on protocols.io. Read more information on sharing protocols at https://plos.org/protocols?utm_medium=editorial-email&utm_source=authorletters&utm_campaign=protocols.

We look forward to receiving your revised manuscript.

Kind regards,

Chunyu Zhang

Academic Editor

PLOS ONE

Journal Requirements:

If the reviewer comments include a recommendation to cite specific previously published works, please review and evaluate these publications to determine whether they are relevant and should be cited. There is no requirement to cite these works unless the editor has indicated otherwise.

[Note: HTML markup is below. Please do not edit.]

Reviewers' comments:

Reviewer's Responses to Questions

**Comments to the Author**

1. If the authors have adequately addressed your comments raised in a previous round of review and you feel that this manuscript is now acceptable for publication, you may indicate that here to bypass the “Comments to the Author” section, enter your conflict of interest statement in the “Confidential to Editor” section, and submit your "Accept" recommendation.

Reviewer #9: All comments have been addressed

Reviewer #10: (No Response)

**********

2. Is the manuscript technically sound, and do the data support the conclusions?

The manuscript must describe a technically sound piece of scientific research with data that supports the conclusions. Experiments must have been conducted rigorously, with appropriate controls, replication, and sample sizes. The conclusions must be drawn appropriately based on the data presented.

Reviewer #9: Yes

Reviewer #10: No

**********

3. Has the statistical analysis been performed appropriately and rigorously?

Reviewer #9: Yes

Reviewer #10: N/A

**********

4. Have the authors made all data underlying the findings in their manuscript fully available?

The PLOS Data policy requires authors to make all data underlying the findings described in their manuscript fully available without restriction, with rare exception (please refer to the Data Availability Statement in the manuscript PDF file). The data should be provided as part of the manuscript or its supporting information, or deposited to a public repository. For example, in addition to summary statistics, the data points behind means, medians and variance measures should be available. If there are restrictions on publicly sharing data—e.g. participant privacy or use of data from a third party—those must be specified.

Reviewer #9: Yes

Reviewer #10: No

**********

5. Is the manuscript presented in an intelligible fashion and written in standard English?

PLOS ONE does not copyedit accepted manuscripts, so the language in submitted articles must be clear, correct, and unambiguous. Any typographical or grammatical errors should be corrected at revision, so please note any specific errors here.

Reviewer #9: Yes

Reviewer #10: No

**********

6. Review Comments to the Author

Please use the space provided to explain your answers to the questions above. You may also include additional comments for the author, including concerns about dual publication, research ethics, or publication ethics. (Please upload your review as an attachment if it exceeds 20,000 characters)

Reviewer #9: The authors have done an excellent job of thoroughly and thoughtfully addressing the concerns raised in the previous round of reviews. The manuscript is now significantly improved and has reached a standard suitable for publication.

I was particularly impressed with the detailed clarification of the core concept of 'cultural appeal,' which was a major point of ambiguity. The authors have now successfully grounded this culturally-specific construct in established international organizational theory (e.g., Schein) and have been transparent about its measurement development process, including a rigorous forward-backward translation. This effort has made the central argument of the paper much more accessible and convincing for an international audience.

Furthermore, the restructuring of the Discussion and Conclusion sections has greatly improved the manuscript's clarity and logical flow, effectively eliminating the previous issues of redundancy. The candid discussion of the study's limitations, particularly the geographical sampling bias and the role of demographic variables, also strengthens the paper's scientific rigor.

In conclusion, all my previous major concerns have been satisfactorily addressed. The manuscript now presents a methodologically sound and insightful study on an important topic. I am pleased to recommend this revised manuscript for acceptance.

Reviewer #10: This paper explores the impacts of cultural appeal and social capital on health care’s emergency capacities.

- I don’t understand why the authors introduce the term ‘cultural appeal,’ and I cannot find any relevant theories or articles on cultural appeal. I am not sure how such research focusing on cultural appeal would benefit the international audience of PLOS One.

- I highly suggest that authors present their survey questions in the main text so that the readers can understand how they constructed variables.

- The survey data mainly came from healthcare providers; therefore, lack of an objective measure of health institutions’ real emergency capacity. Based on Table S2, it seems the dependent variable, health emergency capabilities (e.g., prevention, preparedness, response, and recovery), is health providers’ perceptions of their organizations’ emergency capacities. In considering the wider social impacts of a health emergency, the authors should use more objective measures of emergency capacity.

- The authors found that cultural appeal has positive impacts on emergency management. Based on the S2 Table, I wonder how specific measurement items of cultural appeal, including “having a corresponding culture wall to reflect the organization’s vision and cultural characteristics”, and “implementing socialist core values,” would impact emergency capacities. The authors should explicitly discuss and explain the intrinsic relationship between cultural appeals (e.g., cultural walls, socialist core values) on emergency capacities and their implications to the international audience.

**********

7. PLOS authors have the option to publish the peer review history of their article (what does this mean?). If published, this will include your full peer review and any attached files.

If you choose “no”, your identity will remain anonymous but your review may still be made public.

**Do you want your identity to be public for this peer review?**  For information about this choice, including consent withdrawal, please see our Privacy Policy.

Reviewer #9: **Yes:**  Wang Chunning

Reviewer #10: No

**********

[NOTE: If reviewer comments were submitted as an attachment file, they will be attached to this email and accessible via the submission site. Please log into your account, locate the manuscript record, and check for the action link "View Attachments". If this link does not appear, there are no attachment files.]

While revising your submission, please upload your figure files to the Preflight Analysis and Conversion Engine (PACE) digital diagnostic tool, https://pacev2.apexcovantage.com/. PACE helps ensure that figures meet PLOS requirements. To use PACE, you must first register as a user. Registration is free. Then, login and navigate to the UPLOAD tab, where you will find detailed instructions on how to use the tool. If you encounter any issues or have any questions when using PACE, please email PLOS at figures@plos.org. Please note that Supporting Information files do not need this step.

# Author comment: Author response to Decision Letter 4

**DOI:** 10.1371/journal.pone.0351875.r008

12 Nov 2025

Point-by-point responses to the Reviewers’ comments

Academic Editor

While the manuscript addresses an important topic concerning social capital and emergency capacities in primary healthcare institutions, it still falls short of PLOS ONE’s publication standards in several aspects. In particular, (1) the presence of Chinese-language content in both the response letter and supplementary sections is unacceptable for an international English journal, and (2) the inclusion of Chinese-language sources translated into English does not meet the requirement for internationally retrievable references. In addition, the theoretical construct of “cultural appeal” remains conceptually vague and lacks a clear grounding in established international literature. The manuscript would benefit from reframing this construct within recognized theories of organizational culture or cultural competence, replacing all non-English citations with recent international studies, and ensuring full linguistic and stylistic consistency across the text. A thorough language revision by a professional English editor is strongly recommended before resubmission.

Response（1）: We sincerely thank the reviewer for their insightful comments. We fully understand PLOS ONE’s requirements regarding the use of English for international readership. We have carefully reviewed the entire manuscript and have removed all Chinese-language content from both the response letter and supplementary sections.

Response（2）: We appreciate the reviewer’s suggestion to replace non-English references with internationally retrievable sources. In compliance with this, we have replaced all Chinese-language references with internationally recognized English-language literature. We consulted the latest international studies in the relevant fields and updated the reference list to ensure traceability and international relevance. We have also engaged a professional English editor to perform a thorough language revision, ensuring stylistic consistency and academic quality.

Regarding the theoretical construct of “cultural appeal,” we recognize that this concept may be less familiar in international literature. In this study, we framed “cultural appeal” within the well-established theories of organizational culture and cultural capital. Specifically, cultural appeal refers to the capacity of cultural values and beliefs to elicit emotional resonance and collective identification within a group, which in turn shapes individual behavioral choices in contexts such as emergency management and crisis response.

To further clarify this construct, we draw on Schein’s theory of organizational culture, which emphasizes that shared values and beliefs, embedded in an organization’s strategic goals and ideology, profoundly influence members’ attitudes, motivations, and behaviors. Additionally, we integrate Bourdieu’s theory of cultural capital, which underscores the role of intangible cultural resources (such as values, beliefs, and traditions) in mobilizing collective action. In the context of emergency management, we argue that cultural appeal functions as a form of cultural capital that fosters collective identification and cooperation among members, thus enhancing the emergency response capacity of primary healthcare institutions.

For international readers, this theoretical framing connects the concept of cultural appeal with both organizational culture and cultural capital theories, which have been widely applied in studies of emergency response, crisis management, and organizational behavior. We have revised the manuscript to reflect these theoretical foundations and incorporated relevant international research to enhance its scholarly value.

Cultural appeal is a concept drawn from the Chinese managerial context, referring to the capacity of cultural frames to elicit affective resonance and collective identification within a group, thereby shaping individual behavioral choices. This concept is grounded in Schein’s theory of organizational culture, which posits that shared values and beliefs embedded in an organization’s culture influence members’ attitudes and behaviors. In this study, cultural appeal is framed as a form of intangible cultural capital, drawing from Bourdieu’s theory of cultural capital. Bourdieu emphasizes that cultural capital—particularly intangible forms like values, beliefs, and traditions—plays a crucial role in mobilizing collective action. In the context of emergency management, cultural appeal can enhance cooperation and rapid response, fostering a strong sense of collective identity and shared purpose that improves institutional emergency capacity.

The sources referenced are

Gevorgyan G, & Manucharova, N. The effects of cultural appeal, product involvement, and ethnic identity on attitudes and recall in online advertising. Chinese Journal of Communication, 2015,8(2):196-220.

Michaels L, & Rettig E. A renewable light unto the nations? Modelling the limits of culturally appealing climate frames: a case study from Israel. Journal of Environmental Policy & Planning,2025,27(4):376-389.

Schein, E. H. (2010). Organizational Culture and Leadership. Jossey-Bass.

Bourdieu, P. (1986). The Forms of Capital. In J. G. Richardson (Ed.), Handbook of Theory and Research for the Sociology of Education. Greenwood Press.

[Literature review and hypotheses] (pages 6-7, lines 138–153): “The concept of cultural appeal originates in the Chinese managerial context and refers to the transmission of cultural forces that elicit affective resonance and collective identification within a group, thereby shaping individual behavioral choices. Schein’s organizational culture theory provides theoretical support for this construct: shared beliefs and cultural values are reflected in an organization’s strategic goals and ideology, become embedded in members’ cognitions, and consequently influence their attitudes, motivation, and collaborative behavior [24]. In this sense, cultural appeal strengthens members’ cultural identification and facilitates behavioral mobilization and collective responses. The core idea of cultural appeal also aligns with Bourdieu’s theory of cultural capital. Bourdieu emphasizes that the distribution of cultural capital—especially intangible forms such as traditions, values, and beliefs—can drive collective mobilization and social change [25]. Within this theoretical framework, cultural appeal can be viewed as a form of intangible cultural capital that, by fostering collective identification, promotes active participation and cooperation among organizational members, thereby enhancing overall emergency response capacity.”

Reviewer #10

This paper explores the impacts of cultural appeal and social capital on health care’s emergency capacities.

（1）I don’t understand why the authors introduce the term ‘cultural appeal,’ and I cannot find any relevant theories or articles on cultural appeal. I am not sure how such research focusing on cultural appeal would benefit the international audience of PLOS One.

Response: Thank you for your thoughtful review and valuable feedback. We appreciate the opportunity to clarify the introduction of the term “cultural appeal” and its relevance to our study.

First, cultural appeal is a concept we propose to describe the influence of cultural values and organizational goals in evoking emotional resonance and motivating behavioral change. Drawing from organizational culture theory, we explore how cultural norms and shared values within organizations, particularly in emergency management, can strengthen cooperation and enhance response capacity. While the concept may not be widely established in emergency management, we believe it offers a novel perspective for improving the operational efficiency of primary care institutions, especially during crises. By reinforcing cultural appeal, organizations can foster a sense of responsibility and engagement among their members, leading to more effective emergency responses.

Regarding the absence of relevant theories or studies, we have cited related research that draws from established international theories such as organizational culture and cultural capital. While “cultural appeal” may be more common in Chinese discourse, its core principles align with internationally recognized concepts. For example, research by Gennadi and Naira on cultural identification shows how collective values influence individual behaviors, especially in culturally congruent contexts. Similarly, Michaels and Rettig’s work on culturally appealing climate frames demonstrates how cultural values can dominate public discourse and drive action.

Additionally, Bourdieu’s cultural capital theory provides further support, particularly regarding intangible cultural assets like values and traditions that help mobilize collective action. In our study, we view cultural appeal as a form of cultural capital that enhances emergency response by fostering collective identification and shared organizational values. This framing aligns with Bourdieu’s ideas, where cultural capital can generate emotional energy and social mobilization within organizations.

As for the relevance of this work to the international readership of PLOS ONE, we highlight that the influence of culture on emergency management is gaining global attention, especially in the wake of public health emergencies. By focusing on cultural appeal within Chinese primary healthcare institutions, our study provides insights that are applicable to multicultural contexts worldwide. Understanding how cultural forces impact emergency management can offer valuable lessons for improving public health response capacities across different national and regional settings.

In the revised manuscript, we have further elaborated on the theoretical foundations of cultural appeal and integrated additional international literature to better contextualize the concept and strengthen its relevance for global readers. We believe this conceptual framework can enrich the understanding of emergency management in diverse cultural environments. We greatly appreciate your insightful comments and look forward to your feedback. [Literature review and hypotheses] (pages 6-7, lines 138–153)

The sources referenced are

Gevorgyan G, & Manucharova, N. The effects of cultural appeal, product involvement, and ethnic identity on attitudes and recall in online advertising. Chinese Journal of Communication, 2015,8(2):196-220.

Michaels L, & Rettig E. A renewable light unto the nations? Modelling the limits of culturally appealing climate frames: a case study from Israel. Journal of Environmental Policy & Planning, 2025,27(4):376-389.

（2）I highly suggest that authors present their survey questions in the main text so that the readers can understand how they constructed variables.

Response: Thank you for your valuable suggestion. We agree that presenting the survey questions directly in the main text would help readers better understand the construction of variables. However, due to space limitations, we have opted to include a brief description of the instrument design and variable operationalization in the Methods section (pages 15-17, lines 344-345, 358-359, 361-362,373-376). These descriptions include the number of items, variable dimensions, and representative items, which help clarify the construction of each variable. The full wording of all survey items is provided in the Appendix for those who wish to examine the complete instrument. We believe this approach balances clarity and space constraints while maintaining transparency in our research methodology. We appreciate your thoughtful suggestion, and we trust that these revisions enhance the manuscript’s comprehensiveness and readability.

（3）The survey data mainly came from healthcare providers; therefore, lack of an objective measure of health institutions’ real emergency capacity. Based on Table S2, it seems the dependent variable, health emergency capabilities (e.g., prevention, preparedness, response, and recovery), is health providers’ perceptions of their organizations’ emergency capacities. In considering the wider social impacts of a health emergency, the authors should use more objective measures of emergency capacity.

Response: We understand your point and agree that objective indicators are ideal. In the revision, we explicitly acknowledge this limitation and discuss how future research can incorporate a broader set of objective measures of emergency capacity—particularly response data from actual events and hard preparedness indicators (e.g., stockpiles of emergency supplies, training frequency). Comparative analyses using such data would enable a more comprehensive evaluation.

Regarding our choice to measure primary healthcare institutions’ emergency capacity via healthcare providers’ perceptions of their organizations—rather than purely objective indicators—we believe this approach also has merit. Providers’ assessments can effectively reflect day-to-day readiness and response capacity in routine operations. While objective indicators such as response times, and materials reserves offer direct measures, gathering such data can be hindered by availability, collection burden, and timing. Moreover, objective data that can be compared across regions and institutions may be lacking. Using providers’ subjective evaluations not only captures institutions’ practical emergency capacity but also illuminates cultural and social factors that influence emergency management—factors that are difficult to gauge using traditional objective metrics.

For measurement, we adopted the four widely used dimensions of prevention, preparedness, response, and recovery. This framework has been validated in multiple international studies as an effective standard for assessing health emergency capacity. Specifically, these four dimensions reflect the comprehensive capabilities of health institutions when facing public health emergencies, covering the full cycle from pre-event planning to post-event recovery and thereby ensuring a holistic assessment (Smith).

The reference sources are

Gevorgyan G, & Manucharova N .The effects of cultural appeal, product involvement, and ethnic identity on attitudes and recall in online advertising. Chinese Journal of Communication, 2015,8(2):196-220.

Michaels L ,Rettig E .A renewable light unto the nations? Modelling the limits of culturally appealing climate frames: a case study from Israel. Journal of Environmental Policy & Planning, 2025, 27(4):376-389.

[Research limitations and prospects] (page 37, lines 758–766): “Furthermore, this study assesses health emergency capacity by surveying healthcare providers’ perceptions of their organizations’ capabilities, which relies on subjective perceptions rather than objective measurement. Although such perceptions can reflect providers’ understanding of institutional readiness, they may also be influenced by factors such as cultural appeal and social capital. Accordingly, future research could incorporate a broad set of objective indicators—for example, actual performance in emergency drills, allocation of emergency resources, and outcomes of responses to public health incidents—to provide a more comprehensive assessment of health emergency capacity.”

（4）The authors found that cultural appeal has positive impacts on emergency management. Based on the S2 Table, I wonder how specific measurement items of cultural appeal, including “having a corresponding culture wall to reflect the organization’s vision and cultural characteristics”, and “implementing socialist core values,” would impact emergency capacities. The authors should explicitly discuss and explain the intrinsic relationship between cultural appeals (e.g., cultural walls, socialist core values) on emergency capacities and their implications to the international audience.

Response: Thank you for your valuable comments. In response to your question about the relationship between cultural appeal and emergency management, we have revised the Discussion and Managerial implications sections.

1) Intrinsic relationship between cultural appeal and emergency response capacity.

In line with Schein’s model, we now conceptualize a “culture wall” as a visible cultural arti

Attachment Submitted filename: Response to Reviewers.docx

# Peer review: Decision Letter 4

**DOI:** 10.1371/journal.pone.0351875.r009

**Authors:** Chunyu Zhang

15 Jan 2026

PONE-D-24-36223R4

How do cultural appeal and social capital differ in influencing primary healthcare emergency capabilities in ordinary versus older communities?

PLOS One

Dear Dr. Zhang,

Thank you for submitting your manuscript to PLOS ONE. After careful consideration, we feel that it has merit but does not fully meet PLOS ONE’s publication criteria as it currently stands. Therefore, we invite you to submit a revised version of the manuscript that addresses the points raised during the review process.

After carefully considering the revised manuscript and the reviewers’ latest comments, I find that the paper has improved in presentation and responsiveness. However, substantial concerns remain regarding the conceptualization and theoretical grounding of the core construct, “Cultural Appeal.”

Multiple reviewers independently note that the current theoretical justification relies on literature drawn from marketing, advertising, and political communication, which raises issues of disciplinary mismatch and construct validity. In particular, the way “cultural appeal” is operationalized and theorized does not yet align convincingly with established frameworks in organizational studies, healthcare management, or emergency governance. As a result, the added citations do not sufficiently strengthen the theoretical argument and, in some cases, introduce internal inconsistencies.

Relatedly, the use of Terror Management Theory in the discussion is viewed as insufficiently supported by the study design, as key psychological mechanisms central to the theory were not directly measured. This creates a risk of theoretical overextension.

In addition, as noted by Reviewer 11, the term “culture” may be misleading for an international readership, as the study focuses on organizational or work-related culture rather than ethnic or national culture. The manuscript would benefit from clearer early-stage clarification and a more explicit articulation of why this study matters, including clearer policy and practical implications and identification of intended beneficiaries.

Taken together, these concerns indicate that the manuscript requires substantial theoretical revision, particularly with respect to construct definition, theoretical anchoring, and disciplinary coherence. Further revision would need to go beyond adding or substituting references and instead involve a careful reconsideration of the core conceptual framework.

Please submit your revised manuscript by Mar 01 2026 11:59PM. If you will need more time than this to complete your revisions, please reply to this message or contact the journal office at plosone@plos.org. When you're ready to submit your revision, log on to https://www.editorialmanager.com/pone/ and select the 'Submissions Needing Revision' folder to locate your manuscript file.

Please include the following items when submitting your revised manuscript:

- A letter that responds to each point raised by the academic editor and reviewer(s). You should upload this letter as a separate file labeled 'Response to Reviewers'.
- A marked-up copy of your manuscript that highlights changes made to the original version. You should upload this as a separate file labeled 'Revised Manuscript with Track Changes'.
- An unmarked version of your revised paper without tracked changes. You should upload this as a separate file labeled 'Manuscript'.

If you would like to make changes to your financial disclosure, please include your updated statement in your cover letter. Guidelines for resubmitting your figure files are available below the reviewer comments at the end of this letter.

If applicable, we recommend that you deposit your laboratory protocols in protocols.io to enhance the reproducibility of your results. Protocols.io assigns your protocol its own identifier (DOI) so that it can be cited independently in the future. For instructions see: https://journals.plos.org/plosone/s/submission-guidelines#loc-laboratory-protocols. Additionally, PLOS ONE offers an option for publishing peer-reviewed Lab Protocol articles, which describe protocols hosted on protocols.io. Read more information on sharing protocols at https://plos.org/protocols?utm_medium=editorial-email&utm_source=authorletters&utm_campaign=protocols.

We look forward to receiving your revised manuscript.

Kind regards,

Chunyu Zhang

Academic Editor

PLOS One

Journal Requirements:

If the reviewer comments include a recommendation to cite specific previously published works, please review and evaluate these publications to determine whether they are relevant and should be cited. There is no requirement to cite these works unless the editor has indicated otherwise.

[Note: HTML markup is below. Please do not edit.]

Reviewers' comments:

Reviewer's Responses to Questions

**Comments to the Author**

1. If the authors have adequately addressed your comments raised in a previous round of review and you feel that this manuscript is now acceptable for publication, you may indicate that here to bypass the “Comments to the Author” section, enter your conflict of interest statement in the “Confidential to Editor” section, and submit your "Accept" recommendation.

Reviewer #11: All comments have been addressed

Reviewer #12: (No Response)

**********

2. Is the manuscript technically sound, and do the data support the conclusions?

The manuscript must describe a technically sound piece of scientific research with data that supports the conclusions. Experiments must have been conducted rigorously, with appropriate controls, replication, and sample sizes. The conclusions must be drawn appropriately based on the data presented.

Reviewer #11: Yes

Reviewer #12: Partly

**********

3. Has the statistical analysis been performed appropriately and rigorously?

Reviewer #11: Yes

Reviewer #12: I Don't Know

**********

4. Have the authors made all data underlying the findings in their manuscript fully available?

The PLOS Data policy requires authors to make all data underlying the findings described in their manuscript fully available without restriction, with rare exception (please refer to the Data Availability Statement in the manuscript PDF file). The data should be provided as part of the manuscript or its supporting information, or deposited to a public repository. For example, in addition to summary statistics, the data points behind means, medians and variance measures should be available. If there are restrictions on publicly sharing data—e.g. participant privacy or use of data from a third party—those must be specified.

Reviewer #11: Yes

Reviewer #12: Yes

**********

5. Is the manuscript presented in an intelligible fashion and written in standard English?

PLOS ONE does not copyedit accepted manuscripts, so the language in submitted articles must be clear, correct, and unambiguous. Any typographical or grammatical errors should be corrected at revision, so please note any specific errors here.

Reviewer #11: Yes

Reviewer #12: Yes

**********

6. Review Comments to the Author

Please use the space provided to explain your answers to the questions above. You may also include additional comments for the author, including concerns about dual publication, research ethics, or publication ethics. (Please upload your review as an attachment if it exceeds 20,000 characters)

Reviewer #11: I am looking at the revision already, seems to have undergone multiple rounds. Just a few comments

The word culture and cultural appeal seem a bit misleading. I first thought the paper is about culture in a sense of ethnicity ect., but itis about organization's or work culture. maybe worth to clarify what culture means here early on.

there is no real justification for the study. maybe better highlight that. i was thinking a bit So what? why was it really done. that could also be better reflected at the end in the section about practical considerations. There is not enough about policies and who could benefit from this study.

Reviewer #12: In response to the previous comments, the authors have introduced new English-language references in this revision, attempting to support the theoretical foundation of the core variable "Cultural Appeal" from an international perspective. This demonstrates the authors' willingness to improve the quality of the manuscript.

However, a detailed forensic review and content verification of the newly cited key literature—specifically Gevorgyan & Manucharova (2015) and Michaels & Rettig (2025)—reveals significant issues regarding Disciplinary Mismatch and Construct Validity. The current method of citation fails to strengthen the theoretical persuasiveness; instead, it exposes logical flaws in the theoretical construction, thereby weakening the academic rigor of the study.

1.Irrelevance of Gevorgyan & Manucharova (2015): This reference is a study within the fields of marketing and advertising. Its core independent variable, "Cultural Appeal," refers to rhetorical strategies (e.g., collectivism vs. individualism) used in advertising copy, and its dependent variables are consumer "brand recall" and "purchase attitudes." Equating a consumer's willingness to click on an advertisement in a low-stakes environment with a medical professional's emergency response behavior in a life-or-death situation presents a fundamental logical fallacy. Gevorgyan’s study focuses on Persuasion, whereas your study focuses on Mobilization. Advertising appeal primarily influences transient psychological preferences, whereas organizational cultural appeal operates on enduring professional responsibility. Citing an advertising paper to support a healthcare management theory is far-fetched and conceptually inappropriate.

2.Misapplication of Michaels & Rettig (2025): This article belongs to the fields of environmental policy and political communication. The "culturally appealing climate frames" discussed in the text refer to how politicians utilize grand narratives (e.g., "Start-up Nation") to garner public support. You have cited this work to prove that cultural appeal acts as a positive driver for collective action. However, Michaels' research actually discusses the limitations and fragility of such drivers. Consequently, you have cited literature that may fundamentally contradict your own hypothesis.

3. Issues with the "Cultural Appeal" Construct: "Cultural Appeal" is not a standard construct in international management literature. In the context of Western management science, the activities described in your manuscript—such as establishing "culture walls" and practicing "Socialist Core Values"—would more accurately map to concepts such as Organizational Culture, Safety Climate, or Institutional Logic. Persisting in the use of the non-standard term "Cultural Appeal" has forced you to cite irrelevant literature from advertising and political science, resulting in the aforementioned theoretical inconsistencies.

4.Critique of Terror Management Theory (TMT): The core premise of TMT is that "Mortality Salience" triggers "Worldview Defense." This defense often manifests as cognitive rigidity, exclusion, and irrational behavior, rather than the rational and efficient collaboration required in emergencies. Furthermore, since this study did not measure "death anxiety" or "defense mechanisms," applying TMT appears to be an over-interpretation unsupported by your data. It would be more appropriate to frame major public health events as a "Mortality Salience Context," where the psychological defense mechanisms of medical staff—as the group closest to death—are critical to maintaining their operational capacity.

**********

7. PLOS authors have the option to publish the peer review history of their article (what does this mean?). If published, this will include your full peer review and any attached files.

If you choose “no”, your identity will remain anonymous but your review may still be made public.

**Do you want your identity to be public for this peer review?**  For information about this choice, including consent withdrawal, please see our Privacy Policy.

Reviewer #11: No

Reviewer #12: No

**********

[NOTE: If reviewer comments were submitted as an attachment file, they will be attached to this email and accessible via the submission site. Please log into your account, locate the manuscript record, and check for the action link "View Attachments". If this link does not appear, there are no attachment files.]

To ensure your figures meet our technical requirements, please review our figure guidelines: https://journals.plos.org/plosone/s/figures

You may also use PLOS’s free figure tool, NAAS, to help you prepare publication quality figures: https://journals.plos.org/plosone/s/figures#loc-tools-for-figure-preparation.

NAAS will assess whether your figures meet our technical requirements by comparing each figure against our figure specifications.

Attachment Submitted filename: suggestions on revision.pdf

# Author comment: Author response to Decision Letter 5

**DOI:** 10.1371/journal.pone.0351875.r010

26 Feb 2026

Point-by-point responses to the Reviewers’ comments

Academic Editor

After carefully considering the revised manuscript and the reviewers’ latest comments, I find that the paper has improved in presentation and responsiveness. However, substantial concerns remain regarding the conceptualization and theoretical grounding of the core construct, “Cultural Appeal.”

Multiple reviewers independently note that the current theoretical justification relies on literature drawn from marketing, advertising, and political communication, which raises issues of disciplinary mismatch and construct validity. In particular, the way “cultural appeal” is operationalized and theorized does not yet align convincingly with established frameworks in organizational studies, healthcare management, or emergency governance. As a result, the added citations do not sufficiently strengthen the theoretical argument and, in some cases, introduce internal inconsistencies.

Relatedly, the use of Terror Management Theory in the discussion is viewed as insufficiently supported by the study design, as key psychological mechanisms central to the theory were not directly measured. This creates a risk of theoretical overextension.

In addition, as noted by Reviewer 11, the term “culture” may be misleading for an international readership, as the study focuses on organizational or work-related culture rather than ethnic or national culture. The manuscript would benefit from clearer early-stage clarification and a more explicit articulation of why this study matters, including clearer policy and practical implications and identification of intended beneficiaries.

Taken together, these concerns indicate that the manuscript requires substantial theoretical revision, particularly with respect to construct definition, theoretical anchoring, and disciplinary coherence. Further revision would need to go beyond adding or substituting references and instead involve a careful reconsideration of the core conceptual framework.

Response(1): Thank you very much for your valuable suggestions. In the original manuscript, the construct labeled “Cultural Appeal” was drawn from a China-specific context and is not a standard term in international management or organizational research. Moreover, because its conceptual boundaries were insufficiently specified, our theoretical argument relied on interdisciplinary literature grounded in divergent construct systems, which resulted in dispersed theoretical anchoring and internal inconsistencies. Following the editor’s and reviewers’ recommendations, we therefore corrected this variable and renamed the core construct throughout the manuscript as “Organizational Culture.” This revision is not a mere terminological substitution but a conceptual re-grounding of the focal construct. Specifically, we have consistently replaced the original label with “Organizational Culture,” and, on this basis, re-specified its definitional scope, theoretical positioning, and measurement interpretation to align with established frameworks in organizational studies, healthcare management, and emergency governance. This re-grounding addresses the "construct validity" issues by ensuring that our independent variable is mapped to internationally recognized concept systems. Specifically, we now theorize that the organizational culture in primary healthcare institutions—manifested through artifacts like "culture walls" and espoused "core values"—is internalized by staff, thereby directly influencing their emergency response capabilities.

Response(2):The reviewers noted that the added citations in the previous revision introduced internal inconsistencies. We acknowledge that attempting to "bridge" marketing literature with healthcare management created a dispersed theoretical focus. In the current revision, we have removed all references to advertising and political communication. Instead, we have integrated literature that explores the role of organizational culture in "health system resilience"—defined as the system's ability to prepare, manage, and learn from shocks. We now argue that organizational culture serves as the "social glue" that impacts communication patterns, decision-making, and employee engagement during public health crises.

We rebuilt the theoretical rationale and hypothesis development within organizational studies/healthcare management and used Schein’s organizational culture perspective as a guiding theoretical lens (not as a claim of fully operationalizing all cultural layers). This lens supports the mechanism that shared values and mission consensus can facilitate coordination and collective action in high-stakes response settings. We adopted Edgar Schein’s organizational culture theory as the central theoretical anchor and rebuilt the logic of hypothesis development and discussion accordingly. The specific changes can be found in the revised manuscript: Introduction (page 4, lines 77–88), Literature review and hypotheses (pages 6-7, lines 145–156; page 11, lines 240–246)).

Response(3): We agree with the reviewers that because we did not directly measure death anxiety or specific psychological defense mechanisms, using TMT constituted a "theoretical overextension". Attempting to infer "mortality salience" without direct measurement is speculative and outside the scope of our cross-sectional SEM design. We also specify the deletion locations and the substituted explanations in our point-by-point response to Reviewer 11. The specific changes can be found in the revised manuscript: Discussion (page 29, lines 565–580).

[Introduction] (page 4, lines 77–88) : “Organizational culture” refers to the collective consciousness, shared values, ethical principles, and normative expectations that organizational members develop and uphold through organizational practices under specific socio-economic conditions [14,15]. A constructive organizational culture can subtly motivate members to engage proactively in emergency-related tasks and can strengthen organizational cohesion and response capability. Building on this understanding, organizational culture theory emphasizes that cultural attributes within organizations shape members’ behaviors and decision-making processes[16]. Although organizational culture is largely tacit and intangible, it constitutes one of the most enduring and foundational characteristics of an organization. To date, however, empirical research that treats organizational culture as a key explanatory variable for enhancing public health emergency capacity in primary healthcare institutions remains limited.

[Literature review and hypotheses] (pages 6-7, lines 145–156): The functions of organizational culture include fostering internal integration and coordination by sustaining organizational stability and cultivating a sense of belonging and altruism, all of which guide and shape employees’ behaviors . Schein’s theory of organizational culture further emphasizes that shared beliefs and cultural values are embedded in strategic goals and organizational ideology, becoming internalized in members’ cognitions and thereby influencing their attitudes, motivation, and collaborative behaviors [24].Previous research has demonstrated that shared cultural identity and a positive organizational climate can strengthen organizational cohesion and willingness to cooperate, thereby enhancing risk response and crisis management capabilities [25,26]. An analysis of tertiary hospital organizational culture by Reem et al. revealed a positive correlation between cultural norms, organizational goals, and emergency response capacity [27].

[Literature review and hypotheses] (page 11, lines 240–246): Organizational culture fosters internal cohesion and a shared public mission, which in turn catalyzes and mobilizes the social capital embedded within the community ecosystems of primary healthcare institutions (PHIs). Extant literature (e.g., [42]) suggests that the synergy between cultural orientation and institutional support fortifies both structural and relational social capital in marginalized regions. This reinforcement significantly enhances the "adaptive resilience" of PHIs, enabling more robust recovery and response mechanisms in the aftermath of natural disasters.

Reviewer #11

I am looking at the revision already, seems to have undergone multiple rounds. Just a few comments

The word culture and cultural appeal seem a bit misleading. I first thought the paper is about culture in a sense of ethnicity ect., but itis about organization's or work culture. maybe worth to clarify what culture means here early on.

there is no real justification for the study. maybe better highlight that. i was thinking a bit So what? why was it really done. that could also be better reflected at the end in the section about practical considerations. There is not enough about policies and who could benefit from this study.

Response(1): We are deeply grateful for the constructive feedback provided in the previous rounds. In this final revision, we have meticulously refined the theoretical grounding and practical implications of our study to ensure disciplinary coherence and clarity for an international readership. We have consistently revised the focal construct throughout the manuscript to “Organizational Culture” and removed the term “cultural appeal” to prevent international readers from associating it with ethnic or national culture. Our study focuses exclusively on the “Organizational Culture”. While national culture changes slowly over generations, organizational culture is dynamic and can be shifted by leadership and management over shorter periods to meet strategic goals. By defining organizational culture as "how members relate to each other, to their work, and to the outside world," we clarify that our research is about institutional governance rather than ethnic characteristics. This distinction is critical for the generalizability of the findings to international contexts where different national cultures might still share similar organizational management challenges.

In terms of theoretical grounding, we introduced Edgar Schein’s organizational culture theory as the primary framework and incorporated seminal studies on healthcare organizational culture and emergency preparedness/response, thereby situating our theoretical development firmly within the domains of organizational behavior and health services management. The specific changes can be found in the revised manuscript: Introduction (page 4, lines 77–88).

Response(2): Regarding the study’s purpose, we rewrote the research motivation in the Introduction. We note that prior work on health emergency preparedness has predominantly emphasized the “hard” determinants of capacity—such as large hospitals, material infrastructure, and formalized procedures. In contrast, primary healthcare institutions function as frontline and terminal implementation units within the emergency system, yet they frequently operate under conditions of constrained resources, workforce shortages, and incomplete routines. Under such constraints, variation in emergency capacity may depend more heavily on organizational “soft infrastructure.” Accordingly, we argue that organizational culture constitutes a critical intangible resource in grassroots settings, shaping coordination efficiency, rule compliance, and mobilization capability when material resources are insufficient. Building on this premise, our study examines how organizational culture can partially compensate for limited infrastructure and thereby strengthen emergency capacity in resource-constrained primary healthcare contexts. The specific changes can be found in the revised manuscript: Introduction (page 6, lines 131–141).

Response(3): In response to the reviewer’s “So what?” concern, we revised the concluding synthesis based on our multi-group analysis and articulated the study’s central practical contribution more explicitly. Specifically, we advance an integrated perspective of “cultural empowerment and social-capital synergy” to guide the development of emergency capacity in primary healthcare institutions, directly addressing why the study matters. We also expanded the “Managerial Implications” section to clarify policy relevance and intended beneficiaries. We highlight that strengthening organizational culture provides a concrete pathway for translating the Healthy China strategy and professional ethos into grassroots practice. A "culture wall" in a primary healthcare center is a physical manifestation of "espoused beliefs" that fosters internal integration and a sense of shared purpose. Similarly, "Socialist Core Values" serve as a "soft constraint" and a carrier of specific cultural values that promote collaboration and discipline. By framing these through the lens of informal institutions and organizational behavior, we show how they contribute to a "safety-conscious work environment" and enhance the "disciplined atmosphere" necessary for effective emergency response. Then, for resource-constrained, aged communities, reinforcing structural social capital represents a key leverage point for addressing emergency-capacity shortfalls; for general communities, priority should be placed on cultivating relational ties. These measures are expected to benefit healthcare workers directly and, ultimately, improve outcomes for community residents by supporting evidence-informed “culture–emergency” integration strategies for primary healthcare institutions. The specific changes can be found in the revised manuscript: [Conclusion] (pages 32-33, lines 660–671) and [Managerial implications] (pages 35-37, lines 697–712, lines 722–732, lines 742–749).

[Introduction] (page 6, lines 131–141) : Consequently, focusing on primary healthcare institutions (PHIs), this study integrates organizational culture theory and social capital theory to achieve a threefold objective: (1) to evaluate the direct impacts of organizational culture and social capital on the health emergency capacity of PHIs; (2) to elucidate the mediating mechanism of social capital in the nexus between organizational culture and emergency capacity; and (3) to scrutinize whether these relationships exhibit heterogeneity across diverse community contexts. By addressing these objectives, this research contributes to the extant literature by offering theoretical insights and evidence-based strategies for augmenting the systemic resilience and emergency responsiveness of primary healthcare systems.

[Conclusion] (pages 32-33, lines 669–681) : Multi-group analyses further reveal community-level heterogeneity in these pathways: in general communities, relational and cognitive social capital exert significant effects, whereas in resource-constrained, older communities, structural social capital emerges as the pivotal mediator. The central practical contribution of this study is to demonstrate that strengthening primary healthcare institutions’ emergency capacity is not merely a matter of increasing resource inputs; rather, it is a systemic endeavor that hinges on the synergy between cultural enablement and social-capital mobilization. This perspective offers health authorities and institutional managers an alternative to conventional “hard-capacity” approaches by emphasizing the cultivation of shared values and internal cohesion, together with the strategically targeted development of external community networks. Such efforts can activate and amplify limited emergency resources, thereby translating them into substantive improvements in emergency response effectiveness.

Reviewer #12

1.Irrelevance of Gevorgyan & Manucharova (2015): This reference is a study within the fields of marketing and advertising. Its core independent variable, "Cultural Appeal," refers to rhetorical strategies (e.g., collectivism vs. individualism) used in advertising copy, and its dependent variables are consumer "brand recall" and "purchase attitudes." Equating a consumer's willingness to click on an advertisement in a low-stakes environment with a medical professional's emergency response behavior in a life-or-death situation presents a fundamental logical fallacy. Gevorgyan’s study focuses on Persuasion, whereas your study focuses on Mobilization. Adverti

Attachment Submitted filename: Response to Reviewers.doc

# Peer review: Decision Letter 5

**DOI:** 10.1371/journal.pone.0351875.r011

**Authors:** Chunyu Zhang

19 Apr 2026

PONE-D-24-36223R5

How do organizational culture and social capital differ in influencing primary healthcare emergency capabilities in ordinary versus older  communities？

PLOS One

Dear Dr. Zhang,

Thank you for submitting your manuscript to PLOS ONE. After careful consideration, we feel that it has merit but does not fully meet PLOS ONE’s publication criteria as it currently stands. Therefore, we invite you to submit a revised version of the manuscript that addresses the points raised during the review process.

-->The theoretical framing would benefit from greater conceptual coherence and restraint, avoiding claims that go beyond what is directly supported by the empirical design.

Some interpretations appear to overextend the findings, especially given the cross-sectional and self-reported nature of the data.

The manuscript should more clearly distinguish between empirical associations and theoretical mechanisms, ensuring that conclusions remain closely aligned with the evidence presented.

These issues are resolvable, but they require focused revision and careful recalibration of the manuscript’s positioning, rather than incremental adjustments.

I would therefore like to invite you to submit a revised version that:

Refines and streamlines the theoretical positioning, avoiding overstated claims of mechanism or contribution

Revises the discussion to ensure that interpretations remain proportionate to the data

Clearly acknowledges design limitations and avoids causal language

Please note that this will be considered a final round of revision, and the revised manuscript should fully address these concerns.

Please submit your revised manuscript by Jun 03 2026 11:59PM. If you will need more time than this to complete your revisions, please reply to this message or contact the journal office at plosone@plos.org. When you're ready to submit your revision, log on to https://www.editorialmanager.com/pone/ and select the 'Submissions Needing Revision' folder to locate your manuscript file.

Please include the following items when submitting your revised manuscript:

- A letter that responds to each point raised by the academic editor and reviewer(s). You should upload this letter as a separate file labeled 'Response to Reviewers'.
- A marked-up copy of your manuscript that highlights changes made to the original version. You should upload this as a separate file labeled 'Revised Manuscript with Track Changes'.
- An unmarked version of your revised paper without tracked changes. You should upload this as a separate file labeled 'Manuscript'.

If you would like to make changes to your financial disclosure, please include your updated statement in your cover letter. Guidelines for resubmitting your figure files are available below the reviewer comments at the end of this letter.

If applicable, we recommend that you deposit your laboratory protocols in protocols.io to enhance the reproducibility of your results. Protocols.io assigns your protocol its own identifier (DOI) so that it can be cited independently in the future. For instructions see: https://journals.plos.org/plosone/s/submission-guidelines#loc-laboratory-protocols. Additionally, PLOS ONE offers an option for publishing peer-reviewed Lab Protocol articles, which describe protocols hosted on protocols.io. Read more information on sharing protocols at https://plos.org/protocols?utm_medium=editorial-email&utm_source=authorletters&utm_campaign=protocols.

As the corresponding author, your ORCID iD is verified in the submission system and will appear in the published article. PLOS supports the use of ORCID, and we encourage all coauthors to register for an ORCID iD and use it as well. Please encourage your coauthors to verify their ORCID iD within the submission system before final acceptance, as unverified ORCID iDs will not appear in the published article. *Only*  the individual author can complete the verification step; PLOS staff *cannot*  verify ORCID iDs on behalf of authors.

We look forward to receiving your revised manuscript.

Kind regards,

Chunyu Zhang

Academic Editor

PLOS One

Journal Requirements:

If the reviewer comments include a recommendation to cite specific previously published works, please review and evaluate these publications to determine whether they are relevant and should be cited. There is no requirement to cite these works unless the editor has indicated otherwise.

[Note: HTML markup is below. Please do not edit.]

Reviewers' comments:

Reviewer's Responses to Questions

**Comments to the Author**

1. If the authors have adequately addressed your comments raised in a previous round of review and you feel that this manuscript is now acceptable for publication, you may indicate that here to bypass the “Comments to the Author” section, enter your conflict of interest statement in the “Confidential to Editor” section, and submit your "Accept" recommendation.

Reviewer #13: All comments have been addressed

Reviewer #14: All comments have been addressed

Reviewer #15: All comments have been addressed

**********

2. Is the manuscript technically sound, and do the data support the conclusions?

The manuscript must describe a technically sound piece of scientific research with data that supports the conclusions. Experiments must have been conducted rigorously, with appropriate controls, replication, and sample sizes. The conclusions must be drawn appropriately based on the data presented.

Reviewer #13: No

Reviewer #14: Yes

Reviewer #15: Yes

**********

3. Has the statistical analysis been performed appropriately and rigorously?

Reviewer #13: Yes

Reviewer #14: Yes

Reviewer #15: Yes

**********

4. Have the authors made all data underlying the findings in their manuscript fully available?

The PLOS Data policy requires authors to make all data underlying the findings described in their manuscript fully available without restriction, with rare exception (please refer to the Data Availability Statement in the manuscript PDF file). The data should be provided as part of the manuscript or its supporting information, or deposited to a public repository. For example, in addition to summary statistics, the data points behind means, medians and variance measures should be available. If there are restrictions on publicly sharing data—e.g. participant privacy or use of data from a third party—those must be specified.

Reviewer #13: Yes

Reviewer #14: Yes

Reviewer #15: Yes

**********

5. Is the manuscript presented in an intelligible fashion and written in standard English?

PLOS ONE does not copyedit accepted manuscripts, so the language in submitted articles must be clear, correct, and unambiguous. Any typographical or grammatical errors should be corrected at revision, so please note any specific errors here.

Reviewer #13: No

Reviewer #14: Yes

Reviewer #15: Yes

**********

6. Review Comments to the Author

Please use the space provided to explain your answers to the questions above. You may also include additional comments for the author, including concerns about dual publication, research ethics, or publication ethics. (Please upload your review as an attachment if it exceeds 20,000 characters)

Reviewer #13: The manuscript presents a study examining the relationship between organizational culture, social capital, and emergency response performance using SEM on a relatively strong sample size (N=983), and while the title and abstract are reasonably clear and informative, they remain somewhat overloaded with variables and read more like a technical summary than a compelling research narrative; the introduction has improved compared to earlier versions by clarifying the motivation and focusing on healthcare systems, yet it still lacks a strong and naturally convincing research gap, giving the impression of a constructed rather than inherent contribution; the literature review and theoretical framework represent the weakest component of the study, as the initial conceptual confusion (particularly the use of “cultural appeal”) and inconsistent theoretical grounding were only partially resolved by shifting to organizational culture, resulting in a framework that feels retrofitted rather than conceptually robust; the methodology is generally acceptable, supported by an adequate sample size, appropriate use of SEM, and clear ethical considerations, but it is limited by its cross-sectional design, reliance on self-reported data, and insufficient depth in capturing underlying psychological or organizational mechanisms; the results are statistically sound and clearly presented, with some added value from multi-group analysis, yet they remain predictable and offer limited theoretical insight; the discussion has improved by removing inappropriate theoretical applications such as Terror Management Theory and aligning interpretations more closely with the data, though some degree of over-interpretation persists; the conclusion and implications provide practical relevance for healthcare management and policy, but tend to overstate the contribution given the modest originality of the findings; overall, despite strengths in data size, methodological execution, and applied relevance, the manuscript suffers from fundamental conceptual instability, limited novelty, and a theoretical foundation that appears reactive rather than well-developed, leading to the final recommendation of rejection (borderline major revision), as the core issues are too significant to justify further rounds of revision at this stage.

Reviewer #14: Thank you for submitting the updated version of the manuscript. I think that the paper is well-written and addresses a valuable and relevant gap. You have also addressed the comments that you were given in a proper manner.

Minor issue: Please make sure to fix the referencing error in line 336.

Reviewer #15: After several rounds of revisions, I believe that the authors have effectively addressed the shortcomings pointed out by the reviewers.

**********

7. PLOS authors have the option to publish the peer review history of their article (what does this mean?). If published, this will include your full peer review and any attached files.

If you choose “no”, your identity will remain anonymous but your review may still be made public.

**Do you want your identity to be public for this peer review?**  For information about this choice, including consent withdrawal, please see our Privacy Policy.

Reviewer #13: **Yes:**  The manuscript presents a study examining the relationship between organizational culture, social capital, and emergency response performance using SEM on a relatively strong sample size (N=983), and while the title and abstract are reasonably clear and informative, they remain somewhat overloaded with variables and read more like a technical summary than a compelling research narrative; the introduction has improved compared to earlier versions by clarifying the motivation and focusing on healthcare systems, yet it still lacks a strong and naturally convincing research gap, giving the impression of a constructed rather than inherent contribution; the literature review and theoretical framework represent the weakest component of the study, as the initial conceptual confusion (particularly the use of “cultural appeal”) and inconsistent theoretical grounding were only partially resolved by shifting to organizational culture, resulting in a framework that feels retrofitted rather than conceptually robust; the methodology is generally acceptable, supported by an adequate sample size, appropriate use of SEM, and clear ethical considerations, but it is limited by its cross-sectional design, reliance on self-reported data, and insufficient depth in capturing underlying psychological or organizational mechanisms; the results are statistically sound and clearly presented, with some added value from multi-group analysis, yet they remain predictable and offer limited theoretical insight; the discussion has improved by removing inappropriate theoretical applications such as Terror Management Theory and aligning interpretations more closely with the data, though some degree of over-interpretation persists; the conclusion and implications provide practical relevance for healthcare management and policy, but tend to overstate the contribution given the modest originality of the findings; overall, despite strengths in data size, methodological execution, and applied relevance, the manuscript suffers from fundamental conceptual instability, limited novelty, and a theoretical foundation that appears reactive rather than well-developed, leading to the final recommendation of rejection (borderline major revision), as the core issues are too significant to justify further rounds of revision at this stage.

Reviewer #14: No

Reviewer #15: No

**********

[NOTE: If reviewer comments were submitted as an attachment file, they will be attached to this email and accessible via the submission site. Please log into your account, locate the manuscript record, and check for the action link "View Attachments". If this link does not appear, there are no attachment files.]

To ensure your figures meet our technical requirements, please review our figure guidelines: https://journals.plos.org/plosone/s/figures

You may also use PLOS’s free figure tool, NAAS, to help you prepare publication quality figures: https://journals.plos.org/plosone/s/figures#loc-tools-for-figure-preparation.

NAAS will assess whether your figures meet our technical requirements by comparing each figure against our figure specifications.

# Author comment: Author response to Decision Letter 6

**DOI:** 10.1371/journal.pone.0351875.r012

30 May 2026

Point-by-point responses to the Reviewers’ comments

Academic Editor

1.Refines and streamlines the theoretical positioning, avoiding overstated claims of mechanism or contribution

Response: Thank you for this important comment. We agree that the manuscript should not be positioned as establishing a definitive mechanism or making a broad theoretical breakthrough. In the revised manuscript, we have further reframed the study as a context-specific empirical examination of associations among organizational culture, social capital, and perceived health emergency capacity in primary healthcare institutions.

In the abstract and introduction, we have reformulated the research objective by shifting the emphasis away from “mechanism explanation” and toward testing the path relationships among organizational culture, social capital, and public health emergency capacity using a structural equation modeling approach. The revised manuscript now makes it clear that the focus of this study is to examine the structural relationships among variables and their statistical significance, rather than to offer a definitive explanation of causal mechanisms. The specific changes can be found in the revised manuscript: Abstract (page 2, lines 24-40), Introduction (pages 4-6, lines 83-86; page 11, lines 98-101).

In the hypothesis development sections, we have condensed and integrated the relevant content, removed some repetitive or overly extended theoretical descriptions, and strengthened the direct logical link between organizational culture and social capital. As a result, the theoretical framework is now more focused on the core variables and their relational pathways, thereby improving the overall conciseness and coherence of the manuscript. The specific changes can be found in the revised manuscript: Literature review and hypotheses (page 7, lines 144-152; page 8, lines 174-191；page 9, lines 203-207; page10, lines 214-217; page11, lines 233-240).

At the same time, we have toned down the wording of the theoretical contribution, revising stronger expressions such as “overcoming” and “innovation” to more measured formulations such as “complement existing literature” and “extend prior research.” Overall, the study is now positioned as providing empirical evidence rather than establishing a definitive mechanism. We hope these revisions enhance the rigor of the theoretical presentation and ensure better alignment with the research design. The specific changes can be found in the revised manuscript: Theoretical contribution (page 33, lines 660-667), Managerial implications (page 33, lines 674-680).

[Introduction] (page 2, lines 24-40) : The emergency capacity of primary healthcare institutions is critical to the effectiveness of grassroots emergency management. This study examines the relationships among organizational culture, social capital, and the emergency capacity of primary healthcare institutions using a structural equation modeling approach. A questionnaire survey was conducted among healthcare professionals, yielding 983 valid responses for analysis. The results indicate that organizational culture, as well as structural, relational, and cognitive dimensions of social capital, are significantly associated with the emergency capacity of primary healthcare institutions within the model. In addition, social capital demonstrates mediating roles in the relationship between organizational culture and emergency capacity. Multi-group structural equation modeling further reveals variations across community types: relational social capital shows stronger associations with emergency capacity in ordinary communities, whereas structural social capital is more prominent in older communities. These findings provide empirical evidence on how organizational culture and social capital are linked to emergency capacity in primary healthcare settings, highlighting the importance of both internal cultural development and external social resources in different phases of emergency management.

[Theoretical contribution] (page33, lines 658-673) :This study demonstrated the significant influence of organizational culture and social capital on health emergency capacity, offering several theoretical contributions. By incorporating organizational culture into the analytical framework of emergency preparedness in primary healthcare institutions, this study examines its relationship with health emergency capacity, thereby extending existing perspectives that have predominantly focused on institutional arrangements, resources, and technical capabilities. Moreover, by comparing the differential roles of various types of social capital within the pathways linking organizational culture and emergency capacity, this study further extends the empirical research on the relationship between organizational culture and social capital. A further innovation lies in accounting for heterogeneity at the community level: we systematically tested how organizational culture and the three forms of social capital differentially affect health emergency capacity across community contexts. This community-type perspective—seldom employed in previous research—offers a novel lens for cultivating organizational culture and social capital in primary healthcare institutions according to local community characteristics.

2. Revises the discussion to ensure that interpretations remain proportionate to the data

Response: Thank you for the editor’s valuable suggestion. We have reorganized the structure of the Discussion section to ensure that each subsection is strictly aligned with the results of the structural equation modeling, thereby avoiding speculative interpretations that go beyond the empirical findings.

Specifically, Section 5.1 focuses on interpreting the results of the direct effects, including the path coefficients of organizational culture and different dimensions of social capital on public health emergency capacity. Section 5.2 discusses the differences in indirect effects among various forms of social capital based on the Bootstrap mediation analysis results. Section 5.3 provides a comparative analysis of the differences in path coefficients between general communities and aging communities, based on the multigroup SEM results.

During the revision process, we removed statements related to psychological mechanisms (e.g., mortality salience context and emotional buffering), as these constructs were not directly measured in this study and could otherwise lead to over-interpretation. In addition, we revised the interpretation of the multigroup SEM results by shifting from mechanism-oriented explanations to a more data-driven description of path differences based on Bootstrap results, emphasizing the varying strengths of indirect effects of social capital across different community types. We also refined the discussion of the implications derived from the multigroup analysis to avoid excessive inference beyond the empirical evidence. The specific changes can be found in the revised manuscript: Discussion (page 28, lines 555-559; page29, lines 571-576,580-598; page 31, lines 626-633).

3. Clearly acknowledges design limitations and avoids causal language

Response: Thank you for the editor’s valuable comments. We have further strengthened the description of the study’s design limitations and made consistent revisions throughout the manuscript to avoid presenting path results derived from cross-sectional structural equation modeling (SEM) as strict causal relationships. Specifically, the revisions are as follows:

(1) In the limitations section, we explicitly acknowledge the constraints of the cross-sectional design. Structural equation modeling is primarily used to test theoretical path relationships and assess model fit; therefore, it can provide evidence of associations between variables but does not support strict causal inference. The specific changes can be found in the revised manuscript: Research limitations and prospects (page 36, lines 727-734).

(2) In the discussion section, we have toned down expressions that may imply causality. Stronger terms such as “influence” “effect” and “promote” have been revised to more appropriate formulations consistent with cross-sectional SEM, such as “path relationship” “statistical association” “model-implied relationship” or “significant path relationship within the model”. The specific changes can be found in the revised manuscript: Discussion (page 28, lines 555-559; page29, lines 571-576,580-598; page 31, lines 626-633).

(3) In the conclusion section, we further avoided causal language that exceeds the scope of the research design. We emphasize that the findings primarily reflect the path relationships and variations specified in the theoretical model within the sample, rather than providing definitive confirmation of causal effects. The specific changes can be found in the revised manuscript: Research limitations and prospects (page 32, lines 636-656).

[Research limitations and prospects] (page 36, lines 727-734) : This study presents the following limitations. First, this study adopts a cross-sectional design, which captures data at a single point in time and limits the ability to establish temporal ordering or infer causal pathways among variables. Therefore, although structural equation modeling was employed to examine the path relationships among variables, the results should be interpreted as reflecting statistical associations and model-implied pathways rather than definitive causal relationships. Future research could employ longitudinal or intervention-based designs to further investigate temporal dynamics and potential causal pathways among these variables.

[Conclusion] (page 32, lines 636-656) : This study focuses on primary healthcare institutions and differs from much of the existing literature that emphasizes large hospitals or macro-level emergency systems. From the perspective of community-based, grassroots healthcare service settings, it examines the path relationships among variables associated with health emergency capacity. This perspective provides additional empirical evidence that contributes to the literature on emergency preparedness at the primary healthcare level. The results indicate that organizational culture in primary healthcare institutions is positively associated with health emergency capacity both directly and indirectly through different forms of social capital, with the pathway via relational social capital exhibiting the strongest effect. Multi-group analysis further reveals heterogeneity across community contexts: in ordinary communities, relational and cognitive social capital show significant associations with health emergency capacity, whereas in resource-constrained older communities, the mediating role of structural social capital is more pronounced. These findings suggest that improvements in the emergency capacity of primary healthcare institutions are not only related to resource inputs but are also associated with organizational culture and social capital. From a practical perspective, the results provide a complementary “soft-capacity” lens for health authorities and institutional managers, highlighting the potential value of fostering shared values and internal cohesion, alongside the targeted development of social capital, to support the effective utilization of emergency resources and improve emergency capacity at the primary healthcare level.

Reviewer #13:

The manuscript presents a study examining the relationship between organizational culture, social capital, and emergency response performance using SEM on a relatively strong sample size (N=983), and while the title and abstract are reasonably clear and informative, they remain somewhat overloaded with variables and read more like a technical summary than a compelling research narrative; the introduction has improved compared to earlier versions by clarifying the motivation and focusing on healthcare systems, yet it still lacks a strong and naturally convincing research gap, giving the impression of a constructed rather than inherent contribution; the literature review and theoretical framework represent the weakest component of the study, as the initial conceptual confusion (particularly the use of “cultural appeal”) and inconsistent theoretical grounding were only partially resolved by shifting to organizational culture, resulting in a framework that feels retrofitted rather than conceptually robust; the methodology is generally acceptable, supported by an adequate sample size, appropriate use of SEM, and clear ethical considerations, but it is limited by its cross-sectional design, reliance on self-reported data, and insufficient depth in capturing underlying psychological or organizational mechanisms; the results are statistically sound and clearly presented, with some added value from multi-group analysis, yet they remain predictable and offer limited theoretical insight; the discussion has improved by removing inappropriate theoretical applications such as Terror Management Theory and aligning interpretations more closely with the data, though some degree of over-interpretation persists; the conclusion and implications provide practical relevance for healthcare management and policy, but tend to overstate the contribution given the modest originality of the findings; overall, despite strengths in data size, methodological execution, and applied relevance, the manuscript suffers from fundamental conceptual instability, limited novelty, and a theoretical foundation that appears reactive rather than well-developed, leading to the final recommendation of rejection (borderline major revision), as the core issues are too significant to justify further rounds of revision at this stage.

Response: Thank you for this careful and critical assessment. We have taken your concerns seriously and have revised the manuscript to better match the actual strength and scope of the study. We agree that the manuscript should not be presented as a theory-building paper or as establishing a deep causal mechanism. Accordingly, we have repositioned the manuscript as a context-specific empirical study of associations among organizational culture, social capital, and perceived emergency capacity in primary healthcare institutions. Our revisions address your concerns in the following ways:

(1) We revised the title and abstract to reduce variable overload and to make the study design clearer. The revised title now explicitly identifies the study as a cross-sectional SEM study comparing ordinary and older communities. The abstract now emphasizes the empirical setting, the cross-sectional design, and the interpretation of the results as model-implied associations. The specific changes can be found in the revised manuscript: Abstract (page 2, lines 24-40).

(2) In the introduction, we refined the presentation of the research gap to make it more natural and focused. The revised text now emphasizes that prior research on PHI emergency capacity has primarily concentrated on formal resources and managerial arrangements, while paying less attention to internal organizational culture and community-embedded social capital, and rarely examining their roles within a unified analytical framework. The specific changes can be found in the revised manuscript: Introduction (pages 4-6, lines 83-86; page 11, lines 98-101).

(3) Regarding the literature review, we agree that this section required a stronger conceptual logic. We therefore simplified the hypothesis development section and concentrated on a narrower and more coherent set of arguments. Overly broad or weakly connected theoretical claims were removed, and the transition to the hypotheses was rewritten to clarify that the framework serves as a basis for examining associative relationships, rather than claiming to validate deep causal mechanisms. The specific changes can be found in the revised manuscript: Literature review and hypotheses (page 7, lines 144-152; page 8, lines 174-191；page 9, lines 203-207; page10, lines 214-217; page11, lines 233-240).

(4) In response to your concern regarding conceptual ambiguity, we further simplified the conceptualization of organizational culture. We removed language that could imply conceptual dr

Attachment Submitted filename: Response to Reviewers(1).doc

# Editor report: Decision Letter 6

**DOI:** 10.1371/journal.pone.0351875.r013

**Authors:** Chunyu Zhang

2 Jun 2026

Organizational culture, social capital, and emergency capacity in primary healthcare institutions: A cross-sectional structural equation modeling study comparing ordinary and older communities

PONE-D-24-36223R6

Dear Dr. Zhang,

We’re pleased to inform you that your manuscript has been judged scientifically suitable for publication and will be formally accepted for publication once it meets all outstanding technical requirements.

Within one week, you’ll receive an e-mail detailing the required amendments. When these have been addressed, you’ll receive a formal acceptance letter and your manuscript will be scheduled for publication.

An invoice will be generated when your article is formally accepted. Please note, if your institution has a publishing partnership with PLOS and your article meets the relevant criteria, all or part of your publication costs will be covered. Please make sure your user information is up-to-date by logging into Editorial Manager at Editorial Manager® and clicking the ‘Update My Information' link at the top of the page. For questions related to billing, please contact billing support.

If your institution or institutions have a press office, please notify them about your upcoming paper to help maximize its impact. If they’ll be preparing press materials, please inform our press team as soon as possible -- no later than 48 hours after receiving the formal acceptance. Your manuscript will remain under strict press embargo until 2 pm Eastern Time on the date of publication. For more information, please contact onepress@plos.org.

Kind regards,

Chunyu Zhang

Academic Editor

PLOS One

Additional Editor Comments (optional):

Reviewers' comments:

# Editor report: Acceptance letter

**DOI:** 10.1371/journal.pone.0351875.r014

**Authors:** Chunyu Zhang

PONE-D-24-36223R6

PLOS One

Dear Dr. Zhang,

I'm pleased to inform you that your manuscript has been deemed suitable for publication in PLOS One. Congratulations! Your manuscript is now being handed over to our production team.

At this stage, our production department will prepare your paper for publication. This includes ensuring the following:

* All references, tables, and figures are properly cited

* All relevant supporting information is included in the manuscript submission,

* There are no issues that prevent the paper from being properly typeset

You will receive further instructions from the production team, including instructions on how to review your proof when it is ready. Please keep in mind that we are working through a large volume of accepted articles, so please give us a few days to review your paper and let you know the next and final steps.

Lastly, if your institution or institutions have a press office, please let them know about your upcoming paper now to help maximize its impact. If they'll be preparing press materials, please inform our press team within the next 48 hours. Your manuscript will remain under strict press embargo until 2 pm Eastern Time on the date of publication. For more information, please contact onepress@plos.org.

You will receive an invoice from PLOS for your publication fee after your manuscript has reached the completed accept phase. If you receive an email requesting payment before acceptance or for any other service, this may be a phishing scheme. Learn how to identify phishing emails and protect your accounts at https://explore.plos.org/phishing.

If we can help with anything else, please email us at customercare@plos.org.

Thank you for submitting your work to PLOS ONE and supporting open access.

Kind regards,

PLOS ONE Editorial Office Staff

on behalf of

Dr. Chunyu Zhang

Academic Editor

PLOS One
